# Unravelling the transcriptomic landscape of primary lymphocytic scarring alopecias: systematic review and meta-analysis

**DOI:** 10.3389/fimmu.2025.1651019

**Published:** 2025-08-11

**Authors:** Irene Rivera-Ruiz, Benjamin Ungar, Viviana Dávila-Flores, Jesús Gay-Mimbrera, Pedro J. Gómez-Arias, Miguel Juan-Cencerrado, Carmen Mochón-Jiménez, Esmeralda Parra-Peralbo, Beatriz Isla-Tejera, Teresa López-Viñau López, Emma Guttman-Yassky, Juan Ruano

**Affiliations:** ^1^ Inflammatory Immune-Mediated Chronic Skin Diseases Laboratory, Instituto Maimónides de Investigación Biomédica de Córdoba (IMIBIC), Córdoba, Spain; ^2^ Department of Dermatology, Reina Sofía University Hospital, Córdoba, Spain; ^3^ Department of Dermatology, Icahn School of Medicine at Mount Sinai, New York, NY, United States; ^4^ Department of Pathology, Reina Sofía University Hospital, Córdoba, Spain; ^5^ Department of Pharmacy and Nutrition, Faculty of Biomedical Science and Health, Universidad Europea, Madrid, Spain; ^6^ Department of Pharmacy, Reina Sofía University Hospital, Córdoba, Spain

**Keywords:** scarring alopecia, frontal fibrosing alopecia, lichen planopilaris, central centrifugal cicatricial alopecia, transcriptome meta-analysis, interferon-gamma, JAK-STAT signaling, gene expression profiling

## Abstract

Primary lymphocytic scarring alopecias (PLSAs)—including frontal fibrosing alopecia (FFA), lichen planopilaris (LPP), and central centrifugal cicatricial alopecia (CCCA)—are chronic inflammatory scalp disorders leading to irreversible follicular destruction. Despite overlapping histopathology, their molecular differences remain poorly defined. We performed the first systematic review and transcriptomic meta-analysis of human scalp biopsies in PLSAs (PROSPERO: CRD42024559969), following PRISMA 2020 guidelines. Of 1,080 records screened, eight studies met inclusion criteria; six were eligible for meta-analysis, and two were qualitatively reviewed. The batch-corrected meta-analysis identified shared and subtype-specific transcriptomic alterations. Common features included Th1/IFNγ and JAK/STAT activation, cytotoxic lymphocyte infiltration, and downregulation of epithelial keratins. FFA and LPP showed strong immune activation, while CCCA exhibited lower inflammation but increased mitochondrial stress, lipid metabolism disruption, and fibroblast-associated remodeling. Protein–protein interaction network analysis revealed convergent and divergent molecular modules spanning immune, fibrotic, metabolic, and epigenetic pathways. LPP was uniquely enriched for gene signatures linked to cardiovascular traits, suggesting novel systemic associations. Drug repurposing analyses identified candidate compounds modulating inflammation and metabolism, some reversing inflammatory signatures in brepocitinib-treated samples. This integrated molecular analysis refines our understanding of PLSA subtypes and proposes candidate biomarkers and therapeutic targets, supporting a shift toward biomarker-driven classification and personalized treatment strategies.

**Systematic review registration:**

https://www.crd.york.ac.uk/PROSPERO, identifier CRD42024559969.

## Graphical Abstract

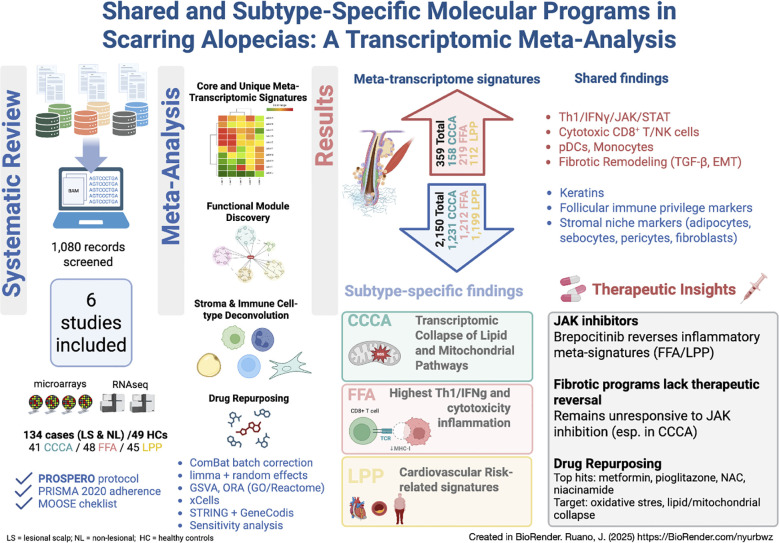

## 1 Introduction

Primary Lymphocytic Scarring Alopecias (PLSAs) are chronic inflammatory disorders marked by irreversible follicular destruction and fibrosis. Although clinical signs such as follicular dropout and scalp atrophy raise suspicion, histopathological confirmation remains essential due to substantial clinical overlap between subtypes (1). The NAHRS classification system (2) remains the standard for distinguishing lymphocytic forms, including lichen planopilaris (LPP), frontal fibrosing alopecia (FFA), and central centrifugal cicatricial alopecia (CCCA).

These disorders primarily affect the follicular infundibulum and isthmus—regions rich in epithelial stem cells—leading to permanent scarring (3). While PLSAs share histologic features, transcriptomic analyses suggest molecular divergence involving immune, fibrotic, and metabolic pathways (4–8).

Unlike alopecia areata (AA), which spares epithelial stem cells and often involves systemic immune activation, PLSAs damage upper follicle structures through local inflammation mediated by MHC upregulation and CD8 ^+^ T-cell cytotoxicity (9, 10). IFN-γ–driven JAK/STAT signaling is a recurrent feature in LPP and FFA (11), leading to interest in JAK inhibitors (JAKi). However, evidence for their efficacy in PLSAs is limited to case reports and small series (12–20).

Despite growing interest, systematic integration of transcriptomic datasets remains lacking. A recent narrative review highlighted metabolic dysregulation in PLSAs (21), but no meta-analysis has yet addressed their molecular underpinnings.

Here, we present the first systematic transcriptome meta-analysis of scalp biopsies in PLSAs, registered in PROSPERO (CRD42024559969), following PRISMA 2020. Our aims were to identify shared and subtype-specific signatures and inform biomarker-driven therapy. We also explored convergence with transcriptomic shifts from a recent phase 2a trial of brepocitinib in scarring alopecia (22).

## 2 Results

### 2.1 Study selection, dataset characteristics, and integration workflow

**Figure 1:**
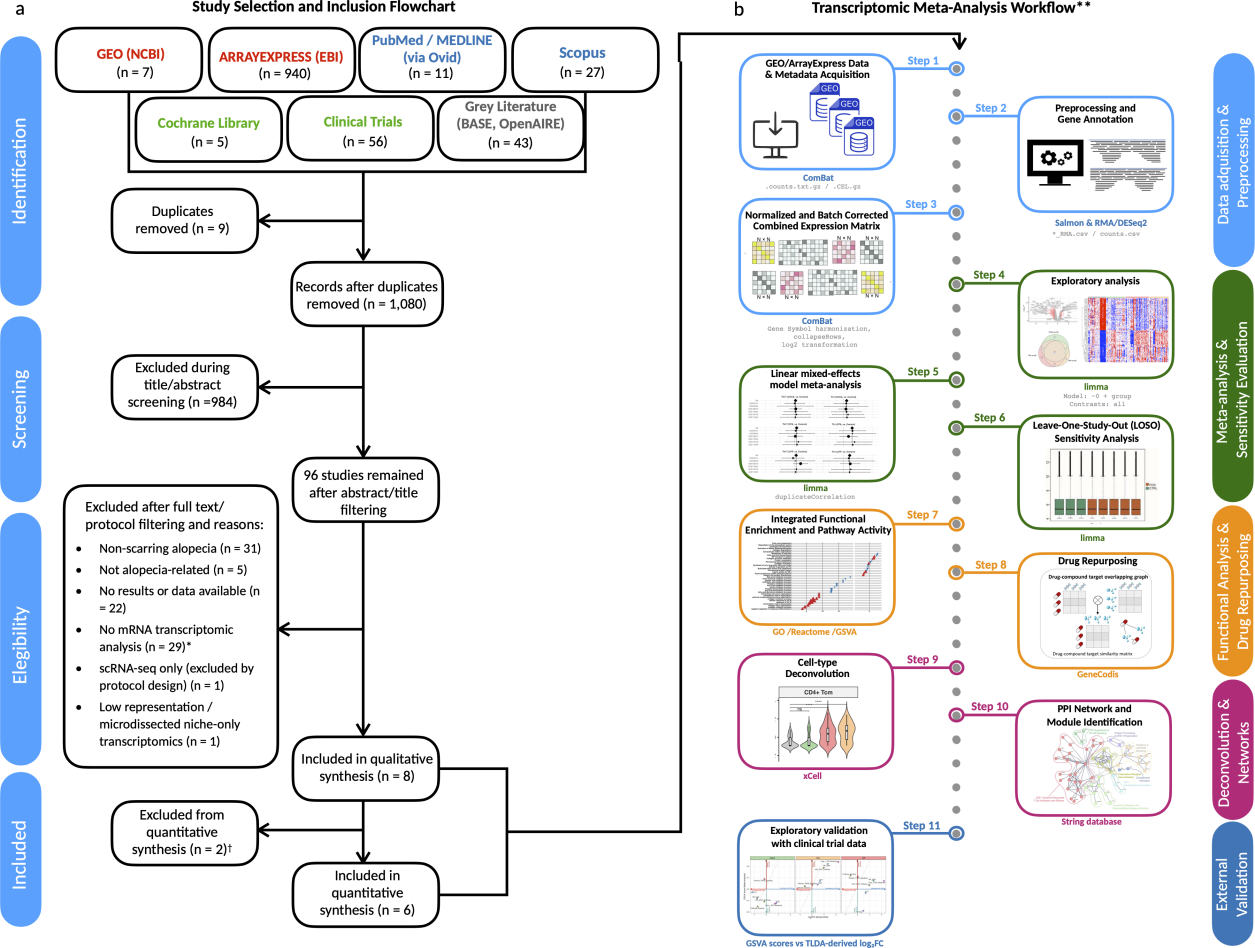
Study selection and transcriptomic meta-analysis workflow. **(a)** PRISMA 2020 flow diagram illustrating the systematic screening and selection of public transcriptomic datasets. From an initial pool of 1,108 records, six datasets met the inclusion criteria and were retained for meta-analysis. *Excluded records included animal models, proteomics-only datasets, genetic/miRNA studies, and clinical reviews lacking gene expression data. **(b)** Overview of the analytical pipeline used for the transcriptomic meta-analysis. All analyses were conducted using R/Bioconductor packages unless otherwise noted. †One additional dataset (David et al., *J Am Acad Dermatol*, 2025) was used for exploratory validation but excluded from meta-analysis due to platform incompatibility (RT-qPCR vs RNA-seq/microarray). PPI network and drug repurposing modules were implemented outside R/Bioconductor using STRING and GeneCodis, respectively. **Workflow steps are explained in detail in the Methods section.

We identified transcriptomic studies from GEO, ArrayExpress, and additional repositories through a structured multi-step screening workflow, as depicted in **Figure 1a**. After removing duplicates (n = 9) and screening 1,080 records by title and abstract, 96 records were retained for full-text or protocol assessment. Of these, 88 were excluded for reasons such as non-scarring alopecia (n = 31), absence of mRNA expression data (n = 29), lack of transcriptomic results (n = 22), or other design limitations (see full criteria in Information).

A total of eight datasets were included in the qualitative synthesis, and six were deemed eligible for meta-analysis based on platform compatibility and data availability (**Supplementary Tables S3**-**S10**). These comprised 134 patients with primary lymphocytic scarring alopecias (PLSAs)—including lesional and non-lesional samples—and 49 healthy controls (HCs), analyzed using bulk RNA-seq (Illumina HiSeq) or Affymetrix microarrays (U133 Plus 2.0 or Clariom S) (**Table 1**). Control representation included 19 individual samples and three pooled samples, each composed of RNA from 10 individuals. Disease subtype distribution included FFA (n = 48), LPP (n = 45), and CCCA (n = 41). PsPB samples were reserved for exploratory analyses and excluded from the meta-analysis due to platform incompatibility (Operon v2 21k). 

**Table 1:** Summary of transcriptomic datasets included in the meta-analysis of primary lymphocytic cicatricial alopecias.

Gene set ID	Disease	PMID	Design	Source	Analysis	Platform	Cases vs controls
GSE186075	CCCA, LPP, FFA	38314944	Observational	Scalp (LS, NL, HC)	Bulk RNA-seq	Illumina HiSeq 3000	LPP (n=30), FFA (n=36), CCCA (n=9); normal controls (n=12)
GSE59131	CCCA, LPP	-*	Observational	Scalp (LS, NL, HC -pool)	Microarray	Affymetrix Human Genome U133 Plus 2.0 Array	LPP affected (n=7), unaffected (n=7); CCCA affected (n=3), unaffected (n=3); normal control pooled (n=1 from 10 samples)
GSE58934	FFA	-*	Observational	Scalp (LS, NL, HC-pool)	Microarray	Affymetrix Human Genome U133 Plus 2.0 Array	FFA affected (n=3), unaffected (n=2); normal control pooled (n=10)
GSE179054	CCCA	35007355 35024684	Observational	Scalp (LS: focal, extensive, severe)	Microarray	Affymetrix Clariom S Assay, Human	CCAA patients (n=16): focal (n=6), limited (n=7), extensive (n=3); no external controls
GSE113052	CCCA	29913259	Observational	Scalp (LS, NL)	Microarray	Affymetrix Clariom S Assay, Human	5 CCCA patients: lesional (n=5), non-lesional (n=5); no external controls
GSE125733	FFA	3589906930850646	Observational	Scalp (LS, HC)	Bulk RNA-seq	Illumina HiSeq 2000	FFA (n=7); normal controls (n=7)
GSE11905	LPP, PPB	19932600	Observational	Scalp (LS, NL)	Microarray	PC Human Operon v2 21k	LPP affected (n=4), unaffected (n=4); PsPB affected (n=4), unaffected (n=4); no external controls

Datasets were retrieved from the NCBI Gene Expression Omnibus (GEO) and include both microarray and bulk RNA-sequencing platforms. Sample sources were exclusively scalp skin biopsies and included lesional (LS), non-lesional (NL), and healthy control (HC) tissues, with some control samples derived from pooled specimens. Platforms used include Affymetrix and Illumina technologies. Where available, PubMed identifiers (PMIDs) for associated publications are provided. The dataset GSE179054 includes lesional samples of varying clinical severity (focal, limited, and extensive) but lacks non-lesional or external control samples. Unpublished datasets were retained in the analysis, and study authors were contacted to clarify the status of their associated publications.

*Unpublished results. CCCA, Central Centrifugal Cicatricial Alopecia; LPP, Lichen Planopilaris; FFA, Frontal Fibrosing Alopecia; PPB, Pseudopelade of Brocq; LS, lesional skin; NL, non-lesional skin; HC, healthy controls; HC-pool, pooled healthy controls; RNA-seq, RNA sequencing; PMID, PubMed Identifier.

All samples underwent harmonized preprocessing, including log_2_ transformation, gene symbol mapping, and batch correction to ensure consistency across studies (**Figure 1b**, **Supplementary Figure S1**). Detailed reasons for exclusion of specific datasets and repository-level breakdowns are reported in **Supplementary Tables S3**-**S8**.

### 2.2 Differential gene expression reveals shared and subtype-specific molecular signatures in PLSAs

**Figure 2:**
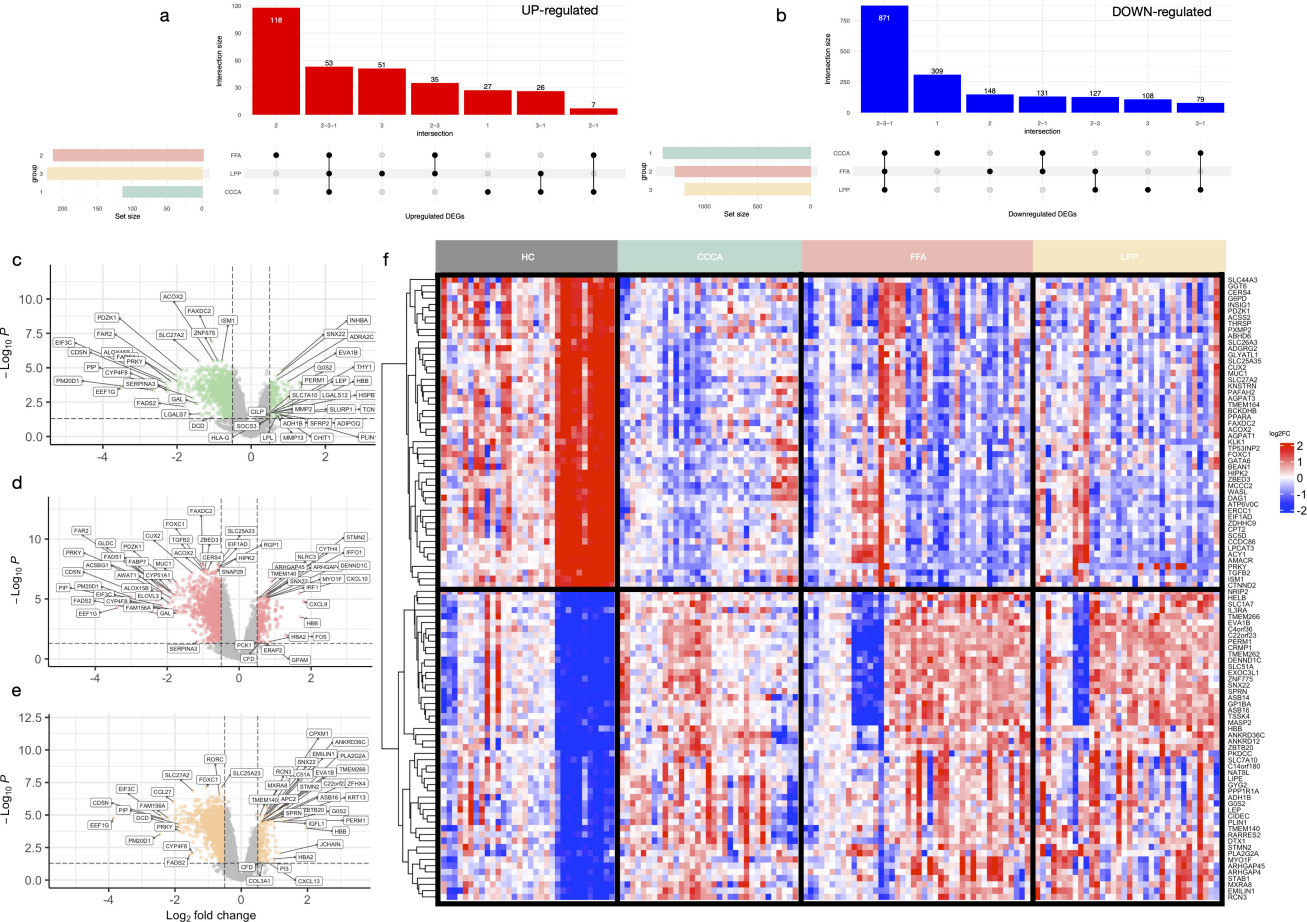
Shared and subtype-specific differentially expressed genes across scarring alopecias. UpSet plots showing the intersections of significantly upregulated **(a)** and downregulated **(b)** genes across the three subtypes of scarring alopecia—frontal fibrosing alopecia (FFA), lichen planopilaris (LPP), and central centrifugal cicatricial alopecia (CCCA)—based on meta-analysis results (adj. p < 0.05; |log_2_FC| > 0.5). Volcano plots of differentially expressed genes in CCCA **(c)**, FFA **(d)**, and LPP **(e)**, comparing lesional samples to healthy controls. Genes meeting the significance thresholds (adj. p < 0.05 and |log_2_FC| > 0.5) are highlighted. **(f)** Heatmap of the top 100 shared DEGs (50 up, 50 down) following ComBat batch correction. Genes are hierarchically clustered; samples are grouped by disease subtype and severity.

A total of 2,509 differentially expressed genes (DEGs) were identified across PLSAs following batch correction and meta-analysis, including 359 upregulated and 2,150 downregulated transcripts (**Figure 2f**, **Supplementary Figures S1**, **S2**). A robust core transcriptomic signature was shared by all three subtypes, comprising 53 consistently upregulated and 871 downregulated genes (**Figures 2a, b**, **Supplementary Table S11**). Subtype-specific DEG counts were: FFA (219 up, 1,212 down), LPP (112 up, 1,199 down), and CCCA (158 up, 1,931 down) (**Figures 2c-e**). Notably, FFA exhibited the highest number of uniquely upregulated genes (n=27), whereas CCCA displayed the greatest number of uniquely downregulated genes (n=309), underscoring both shared and distinct transcriptional programs.

#### 2.2.1 Upregulated genes reflect inflammatory and stress-related programs

The shared upregulated core was dominated by an IFN-γ–driven inflammatory axis, including CXCL10, CXCL9, IFIT1, STAT1, and HLA-DRB1 (**Supplementary Table S11**). Subtype-specific patterns revealed further nuance: CCCA showed induction of neurovascular and epithelial stress-related genes (CLDN5, CYGB, PCDH17), transcriptional regulators (ZNF775, PWWP2B), and oxidative stress mediators (SOD3). FFA was enriched in cytotoxic T cell and interferon-associated transcripts (IRF1, GBP5, GZMB, PRF1, CD8A), along with macrophage activation markers (SIGLEC1, BIRC3). LPP demonstrated selective upregulation of inflammatory lipid mediators (PLA2G2A, PTGDS), fibrotic drivers (ADAMTS12, FSTL3), and neural-epithelial regulators (ZFHX4, ZIC1).

#### 2.2.2 Downregulated genes indicate barrier dysfunction and metabolic collapse

The shared downregulated signature encompassed key regulators of follicular immune privilege (FOXP3, IL10RB, RORC), stemness (LGR5, LHX2), and sebaceous/lipid metabolism (PLIN1, LIPE) (**Supplementary Table S11**). Broad repression of epithelial adhesion genes (CDH1, CLDN1, KRT5) suggested impaired barrier integrity. FFA-specific downregulation involved metabolic and follicular regulators (TGFB2, THRSP, DAG1). LPP exhibited decreased expression of genes involved in lipid metabolism and neuronal signaling (CYP39A1, SYN2, IQCK). CCCA displayed profound suppression of peroxisomal function, detoxification pathways, and epithelial structural genes (PLIN2, MGST1, TRIM24), consistent with a unique metabolic and structural vulnerability.

### 2.3 Functional module analysis reveals mitochondrial and homeostatic collapse across subtypes with divergent inflammatory and epithelial programs

All scarring alopecia subtypes exhibited both shared and distinct patterns of functional dysregulation.

#### 2.3.1 Shared functional modules

**Figure 3:**
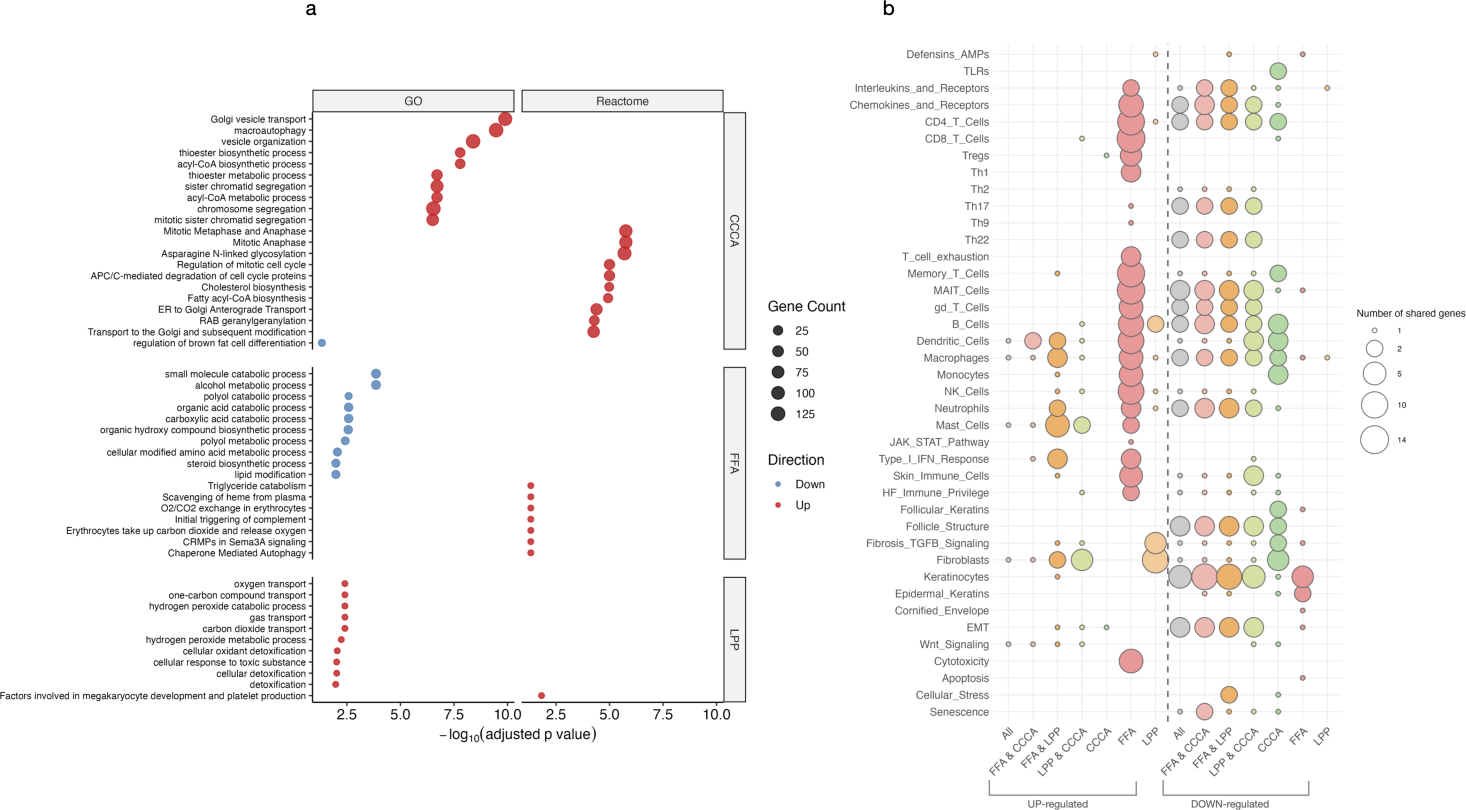
Functional enrichment of upregulated and downregulated genes. **(a)** Bubble plot showing significantly enriched Gene Ontology (GO) and Reactome pathways among upregulated and downregulated genes across scarring alopecia subtypes. **(b)** Bubble plot displaying enrichment of curated custom gene sets representing immune, metabolic, and epithelial programs in the same contrasts. Dot size represents the proportion of genes contributing to each pathway (gene ratio), and colour indicates statistical significance (–log_10_ adjusted p-value).

While shared upregulated genes showed only modest inflammatory activation (**Figure 3a**), downregulated genes revealed a coordinated collapse of essential cellular programs. Early events included suppression of phospholipid remodeling and peroxisomal lipid metabolism, compromising fatty acid β-oxidation, mitochondrial catabolism, and cholesterol biosynthesis (**Figure 3b**). These alterations extended to mitochondrial translation, nucleotide metabolism, and stress response pathways, indicating a pervasive mitochondrial insufficiency. Repression of vesicle trafficking, autophagy, DNA repair, proteasome activity, and desmosomal adhesion further reflected progressive epithelial degeneration and cytoskeletal disintegration.

#### 2.3.2 Subtype-specific modules

FFA was characterized by pronounced upregulation of cytotoxic and interferon-responsive genes, reflecting a strong inflammatory axis. In contrast, CCCA lacked defined inflammatory modules but displayed extensive repression of pathways involved in mitosis, mitochondrial stress responses, DNA repair, transcriptional regulation, and proteostasis—suggesting global failure of cellular homeostasis. LPP exhibited an intermediate profile, with moderate inflammatory enrichment and downregulation of mitochondrial translation and chromatin-associated programs (**Supplementary Figure S3**). Additionally, FFA uniquely showed downregulation of immune privilege mechanisms (e.g., BMP signaling), epidermal differentiation, melanosome biology, and RNA processing pathways, indicating selective disruption of epithelial structure and transcriptional regulation.

### 2.4 Pathway-level analysis revealed common enrichment of fibrotic and epithelial remodeling programs across subtypes

**Figure 4:**
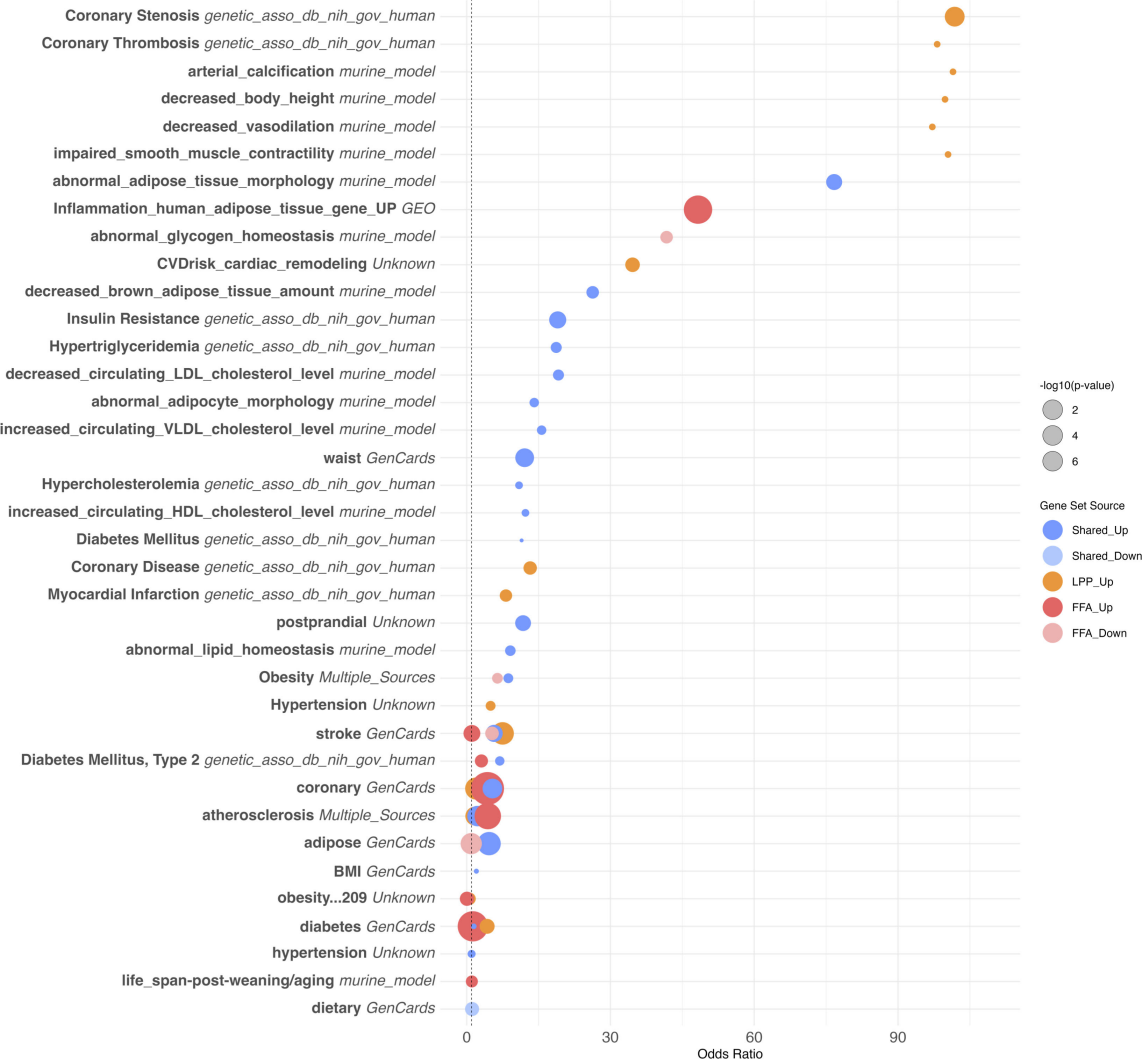
Overrepresentation analysis of cardiovascular and metabolic disease–associated gene sets. Bubble plot showing the top 40 overrepresented gene sets related to cardiovascular, metabolic, and lipid-related phenotypes among differentially expressed genes in scarring alopecias. Gene sets were curated from multiple sources, including human GWAS (e.g. NIH dbGaP), murine models, and public databases such as GeneCards and NCBI GEO. Gene sets are ranked by odds ratio (OR) and adjusted p-value (FDR). Dot size reflects the number of overlapping genes; colour indicates the source of annotation. Notable enrichments include traits associated with coronary artery disease, obesity, diabetes, dyslipidemia, and adipose tissue dysfunction.

Over-representation analysis (ORA) of GO and Reactome terms consistently highlighted activation of TGF-β signaling, fibroblast–stroma interactions, and epithelial–mesenchymal transition (EMT) pathways (**Figure 4a**). Gene set variation analysis (GSVA) confirmed significant downregulation of sebocyte-specific transcriptional programs, particularly in FFA and CCCA, underscoring sebaceous gland loss as a shared pathological feature (**Figure 4b**).

Subtype-specific signatures were also apparent. CCCA showed upregulation of fatty acid metabolism and leukocyte migration pathways, along with repression of negative immune regulators, suggestive of active stromal remodeling with limited immune diversity. In FFA, we observed increased activation of keratinocyte and stromal pathways, elevated macrophage and stromal GSVA scores, but minimal lymphocytic enrichment, pointing to a predominantly epithelial–stromal crosstalk. Conversely, LPP exhibited marked enrichment of keratinocyte, fibroblast, and T cell (CD4 ^+^/CD8 ^+^) signatures, consistent with a T cell–mediated inflammatory phenotype.

### 2.5 Shared stromal expansion with divergent immune landscapes in PLSAs

**Figure 5:**
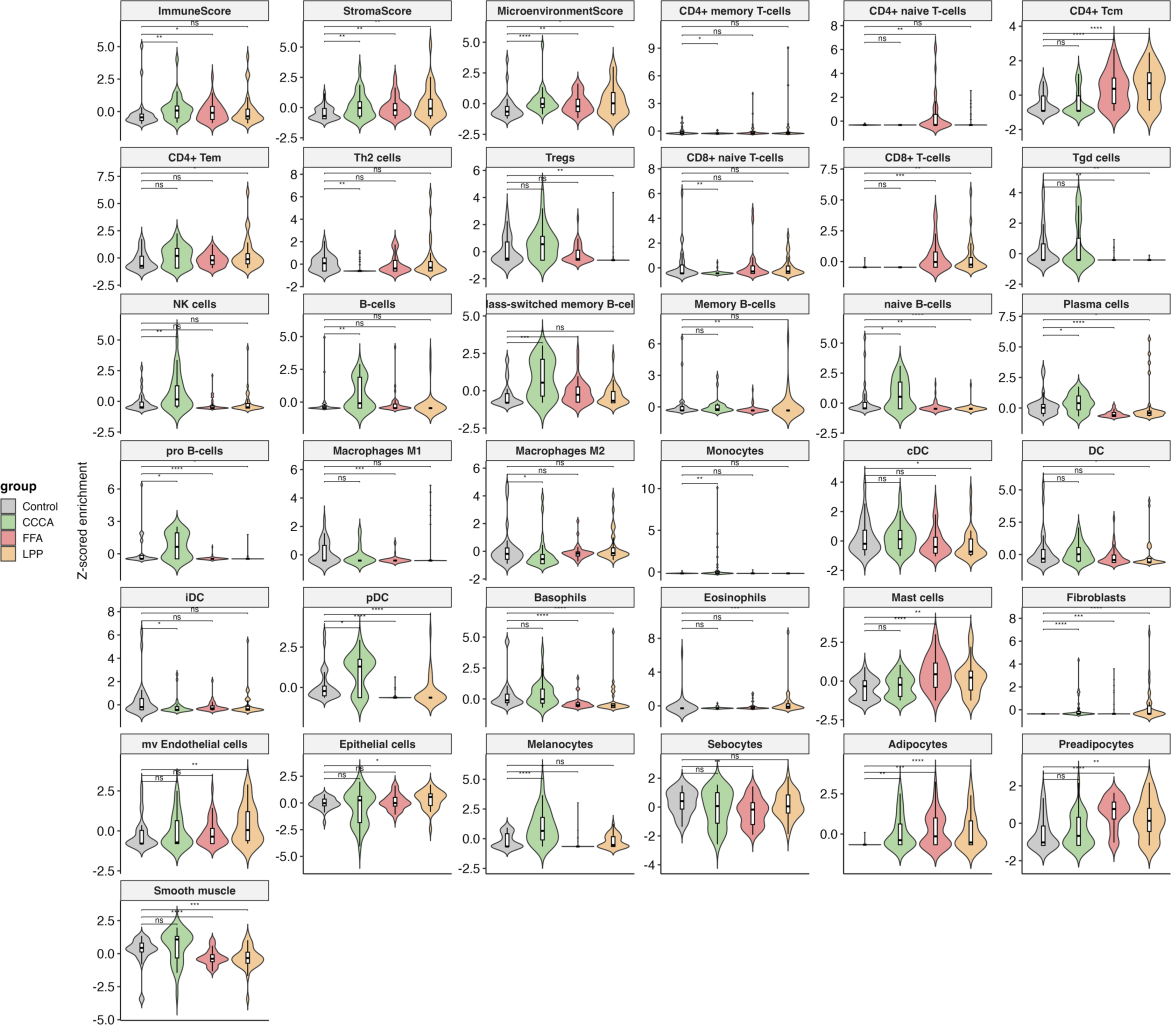
Immune and stromal cell-type deconvolution in scarring alopecia subtypes. Violin plots displaying cell-type enrichment scores (Z-score normalized) obtained via xCell deconvolution analysis across healthy controls, CCCA, FFA, and LPP lesional samples. The analysis includes 36 immune and stromal cell types, as well as composite scores (ImmuneScore, StromaScore, MicroenvironmentScore). Key subtype-specific alterations include increased Tregs and Th2 cells in CCCA; elevated CD8 ^+^ T cells and sebocyte loss in FFA; and strong enrichment of CD4 ^+^ T memory and myeloid dendritic cells (cDC) in LPP. Statistical comparisons were performed using Wilcoxon rank-sum tests with FDR correction; significance is indicated as follows: *p* *< 0.05, **< 0.01, ***< 0.001, **** p < 0.0001, and ns, not significant.

All three PLSA subtypes exhibited a conserved stromal remodeling program, marked by increased fibroblast and adipocyte signatures, alongside elevated StromaScore and MicroenvironmentScore, consistent with a shared fibrotic axis. Sebocyte depletion was most pronounced in FFA, aligning with its characteristic glandular atrophy (**Figure 5**). Despite this shared stromal expansion, immune profiles diverged markedly across subtypes. CCCA displayed robust fibroblast expansion and enrichment of plasmacytoid dendritic cells (pDCs), monocytes, and basophils, but minimal involvement of adaptive immune cells. This innate-skewed profile suggests limited antigen presentation and may explain the lack of overt lymphocytic infiltration. In contrast, LPP exhibited a chronic adaptive immune signature, including CD4 ^+^ central and effector memory T cells, CD8 ^+^ T cells, and loss of naive B and plasma cells, indicating persistent epithelial–immune crosstalk. FFA featured M2 macrophage and keratinocyte enrichment, Treg accumulation, and reduced sebocyte and memory B-cell signatures, suggesting fibrosis driven by innate responses within a disrupted epithelial barrier. Additional distinctions included opposite trends in granulocyte lineages, such as increased basophils in CCCA vs depletion in LPP, and progenitor cell expansion (e.g., pro-B, CLP, CMP), which differed by subtype. Together, these data define a core stromal program shared across scarring alopecias, coupled with disease-specific immune microenvironments that may drive divergent clinical phenotypes and therapeutic responses.

### 2.6 Brepocitinib-induced modulation suggests partial reversibility of inflammatory pathways

**Supplementary Figure S6** explores the alignment between GSVA enrichment scores from the meta-analysis and transcriptomic responses to brepocitinib at week 24 in a phase 2a trial. Inflammatory pathways enriched in LPP and FFA—such as Th1/IFNγ, JAK-STAT, Th17, Th22, and NK cell activation—were downregulated following treatment, indicating potential pharmacologic reversibility. This modulation was most pronounced in FFA, consistent with its strong baseline inflammatory signature. In contrast, CCCA exhibited minimal modulation across these pathways. Notably, follicular keratin programs—suppressed across all subtypes, particularly in CCCA—showed upregulation post-treatment, suggesting partial restoration of follicular gene expression. Fibrotic and extracellular matrix pathways remained unchanged or were further elevated, implying limited efficacy of JAK inhibition on fibrotic remodeling.

### 2.7 Cardiovascular risk factor enrichment suggests immunometabolic intersection in PLSAs

**Figure 6:**
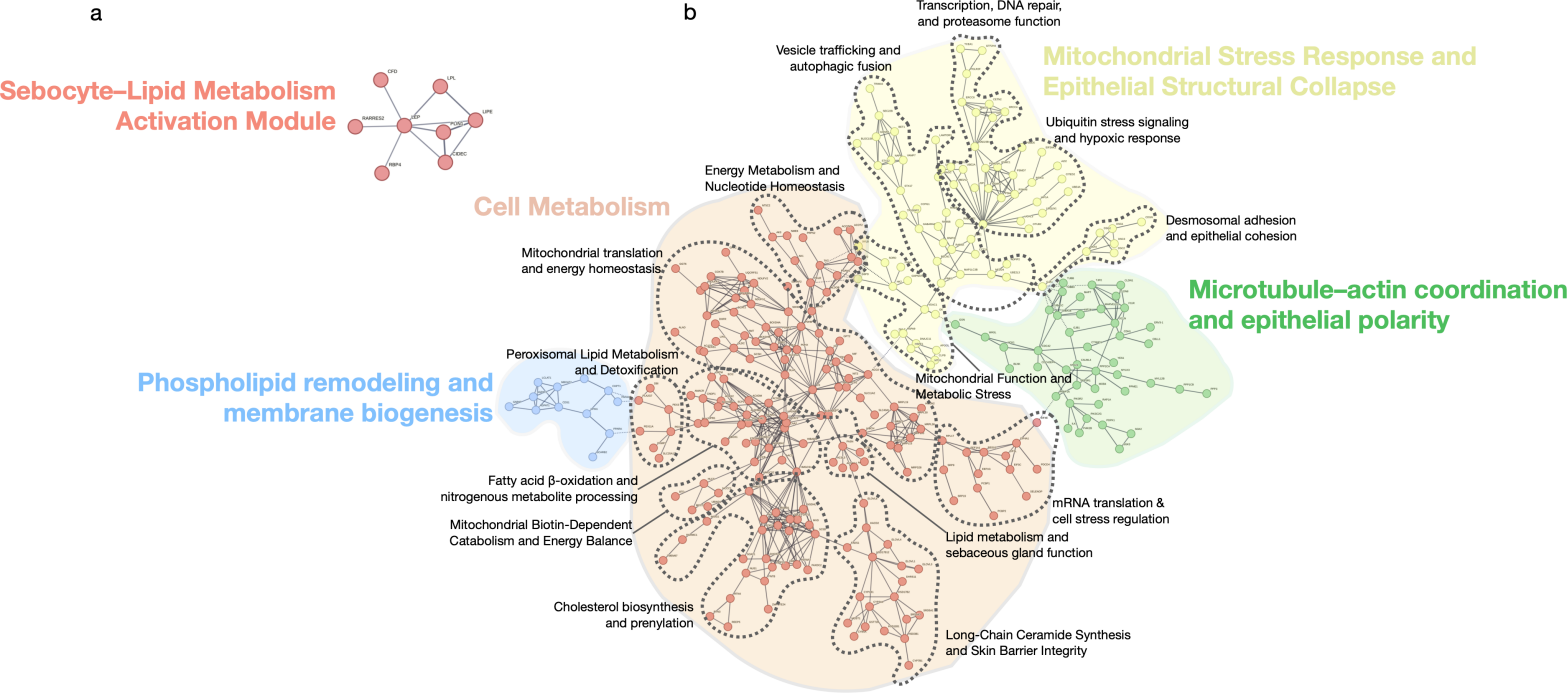
Functional modules of shared differentially expressed genes in scarring alopecias. STRING network showing functional modules derived from the set of shared upregulated and downregulated genes across all scarring alopecia subtypes. Only genes with known protein–protein interactions are included. The network reveals five major clusters: (1) a Sebocyte–Lipid Metabolism Activation Module, (2) a Cell Metabolism hub encompassing lipid biosynthesis and energy regulation, (3) Phospholipid remodeling and membrane biogenesis, (4) Mitochondrial stress response and epithelial structural collapse, and (5) Microtubule–actin coordination and epithelial polarity. Edges indicate confidence-weighted interactions; clusters are annotated based on functional coherence and pathway enrichment. Each cluster contains nested secondary modules, delineated by dashed contour lines that highlight distinct topological and functional substructures within each major domain.

Downregulated genes across PLSA subtypes—particularly in FFA and CCCA—were significantly enriched for cardiometabolic pathways, including adipogenesis, lipid storage, insulin signaling, and brown adipose tissue regulation (**Figure 6**). These transcriptional alterations encompassed reduced expression of key regulators of cholesterol metabolism (e.g., decreased HDL/LDL ratio, triglyceride biosynthesis), adipokine signaling, and glucose homeostasis. In FFA, strong associations were observed with markers of insulin resistance, type 2 diabetes, and impaired adipose morphology, aligning with reported clinical comorbidities. CCCA exhibited similar trends with additional links to arterial stiffness, vascular tone regulation, and myocardial remodeling.

Conversely, upregulated genes were enriched for immune-mediated cardiovascular traits such as monocyte/macrophage activation, vascular inflammation, and coronary artery disease. Traits including coronary stenosis, myocardial infarction, and atherosclerosis were transcriptionally aligned with the inflammatory signatures of PLSAs. Collectively, these findings suggest that chronic scalp inflammation in scarring alopecias may engage systemic cardiovascular risk pathways at both metabolic and immunologic levels, supporting epidemiologic observations and underscoring the relevance of comorbidity screening in affected patients.

### 2.8 Drug repurposing highlights immunometabolic vulnerabilities with partial *in vivo* validation

#### 2.8.1 Transcriptomic reversal prioritizes candidate compounds

Drug repurposing analysis based on transcriptomic reversal identified anti-TNF agents, JAK inhibitors, and interferon modulators as top candidates for FFA, reflecting its interferon-rich inflammatory signature (**Supplementary Table S13**). LPP showed enrichment for immunosuppressants (e.g., methotrexate, azathioprine) and metabolic modulators such as L-arginine and nicotinamide, supporting a cytotoxic T cell–driven mechanism. Although no significant hits emerged for CCCA, fibrates and nicotinamide were enriched across subtypes, aligning with shared mitochondrial and lipid dysfunction, particularly in FFA. Categories lacking mechanistic plausibility—such as antibiotics, CNS drugs, or vaccines—were deprioritized. These findings highlight immune modulation and metabolic correction as convergent therapeutic strategies in PLSAs.

#### 2.8.2 Brepocitinib-treated samples show partial reversal of inflammatory programs

**Figure 7:**
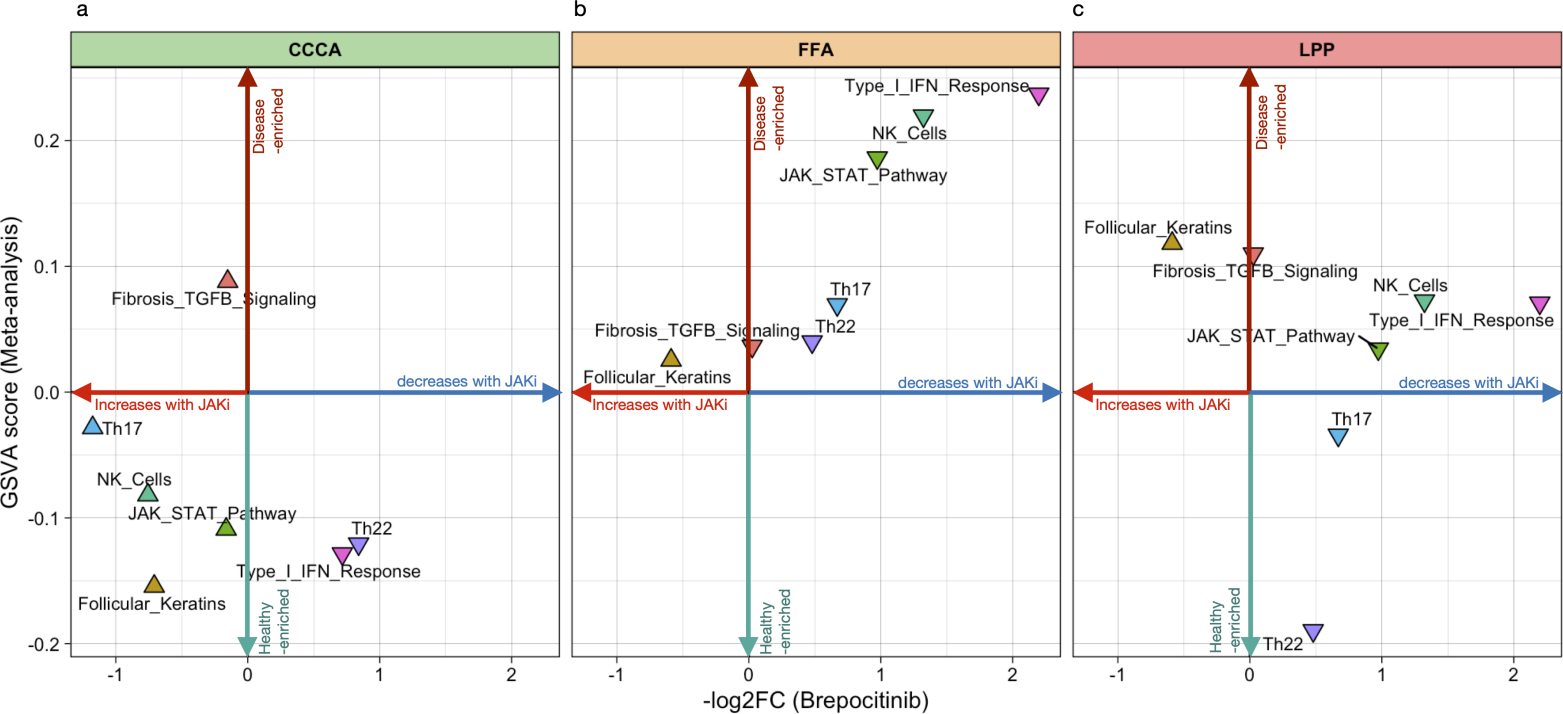
Directional plot of meta-signatures vs brepocitinib-induced transcriptomic effects across scarring alopecia subtypes. directional plots illustrating the relationship between pathway-level transcriptomic enrichment (GSVA meta-analysis scores, vertical axis) and the mean log_2_ fold change after brepocitinib treatment (horizontal axis) in Central Centrifugal Cicatricial Alopecia (CCCA), Lichen Planopilaris (LPP), and Frontal Fibrosing Alopecia (FFA). Each triangle represents a functional signature. Upward-pointing triangles reflect pathway-level changes consistent with a reversal of disease-associated activity, whereas downward-pointing triangles suggest further deviation from control-like expression. The plot is intended as an exploratory comparison to evaluate whether transcriptomic effects of JAK inhibition align with meta-analytic disease signatures across subtypes.

To assess real-world concordance, GSVA enrichment scores from our meta-analysis were compared with log_2_ fold changes after 24 weeks of brepocitinib treatment in a phase 2a trial (22). Proinflammatory signatures—including Th1/IFNγ, JAK-STAT, Th17/Th22, and NK cell activation—were consistently downregulated in FFA and LPP, supporting pharmacologic reversal of key disease pathways (**Figure 7**). CCCA showed minimal modulation. Follicular keratin expression improved in all subtypes, particularly CCCA, while fibrosis-related pathways remained unchanged, suggesting limited anti-fibrotic efficacy of JAK inhibition.

### 2.9 Transcriptomic findings are partially robust to study-level bias, but dataset quality impacts specific signals

#### 2.9.1 Risk of bias assessment identifies variability in study design and data completeness

To evaluate potential sources of heterogeneity, we assessed dataset quality using adapted ROBINS-I criteria, considering platform type, availability of raw data and metadata, control matching, and peer-review status (**Supplementary Table S14**). Low-risk datasets included GSE186075 and GSE125733, both RNA-seq studies with matched individual controls, complete metadata, and peer-reviewed protocols. Moderate to high-risk datasets included GSE59131 and GSE58934, which lacked raw data, used pooled controls, and were unpublished at the time of analysis. GSE113052 and GSE179054, although technically consistent, lacked matched external controls. GSE11905 was excluded entirely due to outdated platform, missing metadata, and poor coverage, resulting in no representation for the PsPB subtype.

#### 2.9.2 Leave-one-study-out sensitivity analysis highlights influential and unstable signals

To evaluate how individual datasets shaped the overall findings, we performed LOSO analyses for each included contrast (**Supplementary Table S15**). Exclusion of GSE186075 or GSE125733 led to marked reductions in the number and effect size of DEGs) notably: Loss of adaptive immune gene enrichment in FFA, and attenuation of epithelial pathways in CCCA. Conversely, exclusion of lower-quality datasets (e.g., GSE59131, GSE58934) sometimes clarified immune–epithelial signals, suggesting that their inclusion may introduce noise.

#### 2.9.3 Gene- and pathway-level robustness identifies core programs with variable sensitivity

At the gene level, several DEGs remained consistently detected across LOSO iterations, including ACO2 and PINK1, highlighting robust signatures of mitochondrial dysfunction. In contrast, genes such as BCKDHA and LPCAT3 (lipid metabolism), SRXN1 (oxidative stress), and FSTL3 (extracellular matrix remodeling) exhibited greater variability depending on dataset composition (**Supplementary Figure S4**). At the pathway level, core immune and stromal programs—such as type I interferon response, cytotoxicity, monocyte infiltration, fibroblast activation, and T cell exhaustion—remained consistently enriched in FFA and LPP, regardless of study exclusion (**Supplementary Figure S5.1** and **S5.2**). In contrast, epithelial and metabolic pathways demonstrated higher sensitivity to dataset variability, particularly in CCCA, underscoring the influence of study quality on the detection of certain transcriptional programs.

## 3 Discussion

This is the first systematic review and meta-analysis of transcriptomic data in PLSAs. By integrating six raw datasets with batch correction, risk of bias assessment, and sensitivity analyses, we identified more DEGs than individual studies, confirming and extending previous findings. Our multilayered approach—covering gene-, module-, pathway-, and cell-level analyses—recovered known signatures and revealed novel programs, including those linked to cardiovascular traits.

We confirmed a conserved fibrotic program across subtypes, marked by fibroblast and adipocyte expansion and increased stromal scores, reflecting structural collapse and fibrosis, consistent with previous studies (4–9). However, immune activation diverged: each subtype exhibited distinct inflammatory profiles not fully captured by prior single-cohort analyses. Beyond validation, we uncovered new pathogenic layers: mitochondrial dysfunction (e.g. impaired oxidative phosphorylation, mitoribosomal dysregulation) in CCCA and FFA; post-transcriptional dysregulation (e.g. spliceosome and RNA-binding modules); and epigenetic repression signatures in CCCA, suggesting transcriptional rigidity and stress adaptation.

Immune deconvolution revealed previously unreported cell states in PLSA. CCCA showed increased plasmacytoid dendritic cells (pDCs)—not detected in GSE186075 and absent from Bao et al.—suggesting type I interferon involvement in fibrotic priming (20). Conversely, mast cell depletion in FFA and LPP, contrary to earlier assumptions (4, 7), may reflect late-stage exhaustion. Divergent dynamics of early progenitors (CLP, CMP, pro-B) were also observed: elevated in CCCA but suppressed in LPP, pointing to subtype-specific recruitment patterns. Finally, Treg enrichment in FFA and LPP—missing in GSE125733 and GSE179054—may reflect inadequate resolution of chronic inflammation (4, 7).

Beyond follicular and immune alterations, we identified significant enrichment of gene sets linked to cardiovascular traits—such as vascular tone, endothelial biology, and atherosclerosis risk—mainly in FFA and LPP. These included PPAR signaling, eNOS activation, and adipokine pathways. While classical metabolic traits (e.g., dyslipidaemia, insulin resistance) showed weaker signals, recurrent lipid metabolism modules reinforce a possible cardiometabolic link. In contrast, CCCA lacked these enrichments, supporting subtype-specific systemic associations.

These findings require cautious interpretation. Over-representation analysis does not account for expression direction, and many enriched cardiometabolic pathways—particularly lipid and adipocyte modules—were downregulated, indicating repression or collapse rather than activation, especially in fibrotic stages. Moreover, the clinical significance of these signatures is unclear. While FFA and LPP showed strong transcriptomic links to cardiovascular traits, CCCA did not—contrasting with epidemiological reports of higher cardiometabolic comorbidity in CCCA (22, 23). This apparent discrepancy may reflect multiple non-exclusive explanations: (i) transcriptomic profiling of lesional scalp tissue may fail to capture systemic cardiometabolic alterations; (ii) clinical comorbidities may be mediated by distinct pathways not reflected at the skin level; (iii) differences in disease chronicity, stage, or severity between cohorts may affect metabolic signatures; and (iv) the lower baseline inflammation and reduced immune cell infiltration in CCCA may mask systemic signals. Prospective clinical-transcriptomic studies are needed to resolve these discrepancies.

Our drug repurposing analysis identified candidate compounds targeting key inflammatory, metabolic, and oxidative stress pathways, including JAK inhibitors, methotrexate, statins, metformin, pioglitazone, nicotinamide, and N-acetylcysteine. Several of these agents have shown preliminary efficacy in cicatricial alopecias or related conditions. For instance, metformin has attenuated profibrotic signatures in CCCA and shown benefit in topical application (24, 25); pioglitazone has demonstrated efficacy in LPP in clinical trials and case reports (26, 27); and N-acetylcysteine and pentoxifylline have yielded positive effects on symptoms and tolerability in controlled trials (27). β-Nicotinamide mononucleotide enhances hair growth by reducing oxidative stress in preclinical models (28), and additional targets related to EMT are being explored (29). A recent network meta-analysis also informs comparative efficacy in LPP (30). Given that many profibrotic pathways appear transcriptionally repressed in lesional scalp, these compounds may be most effective in early or transcriptionally active phases.

To further explore this pharmacological relevance, we assessed whether brepocitinib-induced transcriptomic shifts align with disease-associated GSVA meta-signatures (21). In LPP and FFA, immune pathways (Th1/IFNγ, JAK-STAT, NK cell activation) were downregulated post-treatment, mirroring their baseline enrichment and supporting pharmacological tractability. Th17 and Th22 axes exhibited milder changes. By contrast, brepocitinib had limited impact in CCCA, suggesting lower responsiveness. Fibrotic signatures were unaffected across all subtypes, indicating that anti-inflammatory agents alone may be insufficient to reverse established fibrosis. Increased follicular keratin expression, especially in CCCA, likely reflects partial epithelial restitution rather than true remodeling.

## 4 Conclusion

This meta-analysis defines a reproducible transcriptomic framework for PLSAs, revealing shared fibrotic signatures alongside distinct immune–metabolic programs. FFA, LPP, and CCCA emerge as biologically divergent entities, supporting a stratified therapeutic approach. While inflammatory pathways such as IFN and JAK-STAT are prominent in FFA/LPP and partially reversible with JAK inhibition, persistent fibrosis underscores the need for subtype-specific antifibrotic strategies.

Future studies using single-cell or spatial transcriptomics may help disentangle the contribution of distinct immune and epithelial cell populations to the pathogenesis of each PLSA subtype, validating key pathways uncovered in our analysis—such as JAK-STAT signaling, mitochondrial stress, and immune–metabolic crosstalk—within their cellular contexts. Spatially resolved analyses could also refine the topographical organization of immune–epithelial interactions within the follicular unit, providing a mechanistic map to guide therapeutic targeting.

### 4.1 Strengths and limitations

This systematic review and meta-analysis adheres to PRISMA 2020 guidelines and a pre-registered PROSPERO protocol, enhancing transparency and minimizing selection bias. We integrated six harmonized transcriptomic datasets—including two previously unpublished—across RNA-seq and microarray platforms. To reduce technical variability, we applied batch correction and linear mixed-effects modelling. Risk of bias was formally assessed, and robustness was further evaluated through leave-one-study-out (LOSO) sensitivity analyses. Our multilayered framework—spanning gene-level, modular, pathway-level, and cell-type deconvolution analyses—enabled the detection of conserved and subtype-specific disease signatures not apparent in individual studies.

In contrast to the recent narrative review by Bao et al. (61)—which emphasized dysregulated lipid metabolism and sebaceous gland atrophy in CCCA without applying quantitative synthesis—our meta-analysis offers a statistically rigorous framework that integrates data across multiple transcriptomic studies. While we confirmed key features noted by Bao et al., including sebocyte depletion and metabolic dysfunction, our analysis also revealed novel, subtype-specific insights: distinct immune cell infiltration profiles, epithelial–mitochondrial collapse, and transcriptomic links to cardiovascular traits, particularly pronounced in FFA and LPP. Furthermore, by incorporating unpublished datasets and conducting drug repurposing analyses, we identified tractable molecular targets with potential therapeutic relevance.

Nonetheless, several limitations should be acknowledged. Bulk transcriptomics inherently lacks single-cell resolution, limiting precise cellular attribution. Additionally, incomplete clinical metadata (e.g., disease stage, treatment exposure) in some studies restricted the possibility of subgroup stratification. To enhance statistical power and subtype coverage in this rare disease group, we included a subset of studies with moderate to high risk of bias (e.g., pooled controls, unavailability of raw data, or unpublished status). Their inclusion was justified by the scarcity of available data and supported by LOSO analyses, which confirmed that core disease signatures—particularly those involving immune–fibrotic programs—remained stable. In fact, the exclusion of lower-quality datasets occasionally sharpened signal detection, though we recognize that these studies may introduce analytical noise and should be interpreted cautiously.

Finally, the PsPB subtype was excluded from integrative meta-analysis due to platform incompatibility (Operon v2, 21k array). However, exploratory reanalysis of its data (GSE11905) revealed substantial transcriptomic overlap with LPP, including upregulation of interferon-stimulated genes (e.g., MX1, OASL, STAT1), CD8 ^+^ T cell cytotoxic markers (e.g., GZMB, PRF1), and downregulation of follicular keratins and desmosomal genes. These immune-epithelial signatures suggest that PsPB may lie within the LPP spectrum. Although these findings derive from a single, legacy microarray dataset and cannot support definitive conclusions, they highlight the need for renewed transcriptomic profiling of PsPB using modern RNA sequencing platforms to clarify its nosological status almost two decades after its initial description.

## 5 Materials and methods

For full materials and methods, please refer to the **Materials.**

### 5.1 Protocol registration

This systematic review and meta-analysis followed PRISMA 2020 guidelines and was prospectively registered in PROSPERO (ID: CRD42024559969) (31).

### 5.2 Data sources and eligibility criteria

Transcriptomic studies were systematically searched in GEO (32), ArrayExpress (33), MEDLINE, ClinicalTrials.gov, and grey literature sources up to March 23, 2024. Eligible studies included RNA-seq or microarray data from human scalp biopsies of patients with primary lymphocytic cicatricial alopecias (FFA, LPP, CCCA, PsPB) and matched healthy controls or baseline lesional samples. Exclusion criteria included non-human models, non-scarring alopecias, incompatible data formats, or non-mRNA profiling.

### 5.3 Study selection and data extraction

Two reviewers independently screened studies and extracted metadata including alopecia subtype, platform, sample type and size, control characteristics, and completeness of annotations.

### 5.4 Risk of bias and dataset quality

Dataset-level risk of bias was assessed using a modified ROBINS-I tool adapted for transcriptomic studies, covering six domains: sample selection, platform consistency, metadata completeness, case–control comparability, peer-review status, and conflict of interest disclosure (34, 35).

### 5.5 Data processing and differential expression analysis

Microarrays were normalized using RMA; RNA-seq data using variance-stabilizing transformation (VST). DEGs were identified using limma (with voom for RNA-seq), applying FDR-adjusted *p* < 0.05 and |log_2_FC| > 0.5. Intra-dataset correlation was addressed using *duplicateCorrelation*.

### 5.6 Meta-analysis, deconvolution and robustness

Batch correction across datasets was performed with ComBat. Meta-analysis was conducted using limma with random-effects modeling. Cell-type proportions were estimated with xCell, and LOSO sensitivity analysis was used to evaluate robustness and identify influential datasets.

### 5.7 Functional and cardiometabolic pathway enrichment

We applied Overrepresentation Analysis (GO, Reactome) and GSVA on R using curated and custom gene sets representing immune activation, epithelial remodeling, fibrosis, lipid metabolism, cellular stress, and cardiometabolic traits.

### 5.8 Protein–protein interaction and functional module discovery

PPI networks were built using STRING (score ≥ 0.7), limited to DEGs, and functionally clustered into disease-specific modules (36).

### 5.9 Drug repurposing analysis

Candidate compounds were identified through enrichment of DEG modules (from PPI networks) against LINCS L1000 and PharmGKB using GeneCodis 4 (37), prioritizing drugs predicted to reverse pathogenic expression patterns.

### 5.10 Comparison with therapeutic transcriptomic signatures

Transcriptomic profiles from our meta-analysis were directionally compared with TLDA-based brepocitinib trial results at week 24, using visual plots of GSVA and log_2_FC for exploratory interpretation.

### 5.11 Statistical analysis and threshold criteria

Differential gene expression was assessed using the *limma* package, applying empirical Bayes moderation and a random-effects model with the *duplicateCorrelation* function to account for within-study dependencies. Meta-analyses were conducted separately for each contrast (FFA vs. control, LPP vs. control, CCCA vs. control), and results were considered significant at false discovery rate (FDR) adjusted p < 0.05 and |log_2_FC| > 0.5.

For pathway-level analysis, Gene Set Variation Analysis (GSVA) was performed on batch-corrected expression data using both curated (MSigDB) and custom gene sets related to immunity, epithelial biology, fibrosis, metabolism, and cardiovascular traits. Differences between groups were assessed using Wilcoxon rank-sum tests, with FDR correction.

Overrepresentation analysis (ORA) was performed using clusterProfiler and GeneCodis, employing hypergeometric testing with Benjamini-Hochberg correction. Only pathways with adjusted *p* < 0.05 and minimum gene count ≥ 3 were retained.

In drug repurposing analyses, candidate compounds from LINCS L1000 and PharmGKB were ranked by enrichment scores and *p*-values derived from permutation-based gene set tests. Reversal of disease expression signatures was prioritized using negative connectivity scores and pathway concordance.

All analyses and plots were implemented in R version 4.3.2 and Bioconductor 3.17.

## Data Availability

Publicly available datasets were analyzed in this study. This data can be found here: https://www.ncbi.nlm.nih.gov/geo/.

## Glossary

AA: Alopecia areata
AP-1: Activator protein 1
BMP: Bone morphogenetic protein
CCCA: Central centrifugal cicatricial alopecia
CD: Cluster of differentiation
CLP: Common lymphoid progenitor
CMP: Common myeloid progenitor
DEGs: Differentially expressed genes
EMT: Epithelial–mesenchymal transition
FDR: False discovery rate
FFA: Frontal fibrosing alopecia
GEO: Gene Expression Omnibus
GO: Gene Ontology
GSVA: Gene Set Variation Analysis
GWAS: Genome-wide association study
HC: Healthy control
JAK: Janus kinase
JAKi: Janus kinase inhibitor
LIMMA: Linear Models for Microarray and RNA-Seq Data
LINCS: Library of Integrated Network-based Cellular Signatures
LOSO: Leave-one-study-out
LPP: Lichen planopilaris
MHC: Major histocompatibility complex
miRNA: MicroRNA
NK: Natural killer
NL: Non-lesional
OR: Odds ratio
ORA: OverRepresentation Analysis
PLSAs: Primary lymphocytic scarring alopecias
pDC: Plasmacytoid dendritic cell
PPI: Protein–protein interaction
PRISMA: Preferred Reporting Items for Systematic Reviews and Meta-Analyses
PsPB: Pseudopelade of Brocq
qPCR: Quantitative polymerase chain reaction
RMA: Robust multichip average
RNA-seq: RNA sequencing
RoB: Risk of bias
RT-qPCR: Reverse transcription quantitative PCR
SRA: Sequence Read Archive
STRING: Search Tool for the Retrieval of Interacting Genes/Proteins
STAT: Signal transducer and activator of transcription
Th1: T helper type 1
Th17: T helper type 17
Th22: T helper type 22
TLDA: TaqMan Low-Density Array
Treg: Regulatory T cell
xCell: Cell-type enrichment analysis tool
