# Supplementary material for: Unravelling the transcriptomic landscape of primary lymphocytic scarring alopecias: systematic review and meta-analysis
Source: Front Immunol. 2025 Aug 11;16:1651019. doi: 10.3389/fimmu.2025.1651019 (PMC12375577; doi:10.3389/fimmu.2025.1651019)
Supplement: Supplementary file 1 [file Supplementaryfile1.docx]

**Unravelling the Transcriptomic Landscape of Primary Lymphocytic Scarring Alopecias: Systematic Review and Meta-Analysis**

Irene Rivera-Ruiz^1,2,#^, Benjamin Ungar^3,#^, Viviana Dávila-Flores^4^, Jesús Gay-Mimbrera^1^, Pedro J. Gómez-Arias^1,2^, Miguel Juan-Cencerrado^1,2^, Carmen Mochón-Jiménez^1,2^, Esmeralda Parra-Peralbo^5^, Beatriz Isla-Tejera^3^, Teresa López-Viñau López^3,*^, Emma Guttman-Yassky^3,7^, Juan Ruano^1,2,*^

^1^: Inflammatory Immune-Mediated Chronic Skin Diseases Laboratory, IMIBIC, 14004 Córdoba, Spain

^2^: Department of Dermatology, Reina Sofía University Hospital, 14004 Córdoba, Spain

^3^: Department of Dermatology, Icahn School of Medicine at Mount Sinai, New York, New York.

^4^: Department of Pathology, Reina Sofía University Hospital, 14004 Córdoba, Spain

^5^: Department of Pharmacy and Nutrition, Faculty of Biomedical Science and Health, Universidad Europea, 28670 Madrid, Spain

^6^: Department of Pharmacy, Reina Sofía University Hospital, 14004 Córdoba, Spain

^7^: Laboratory of Inflammatory Skin Diseases, Icahn School of Medicine at Mount Sinai Hospital, New York, NY, USA

I.R.-R. and B.U. contributed equally to this work and share first authorship. E.G.-Y. and J.R. are senior co-authors.**¡**

MOOSE (Meta-analyses Of Observational Studies in Epidemiology) Checklist

- A reporting checklist for Authors, Editors, and Reviewers of Meta-analyses of Observational Studies.
- You must report the page number in your manuscript where you consider each of the items listed in this checklist.
- If you have not included this information, either revise your manuscript accordingly before submitting or note N/A.

# Reporting of Background

| **Reporting Criteria** | **Reported (Yes/No)** | **Reported on Page No.** |
| --- | --- | --- |
| Problem definition | Yes | 7,9,10 |
| Hypothesis statement | Yes | 26, 27, and Supplementary Materials |
| Description of Study Outcome(s) | Yes | NA |
| Type of exposure or intervention used | Yes | NA |
| Type of study design used | Yes | 26,27 |
| Study population | Yes | 9 |

# Reporting of Search Strategy

| **Reporting Criteria** | **Reported (Yes/No)** | **Reported on Page No.** |
| --- | --- | --- |
| Qualifications of searchers (eg, librarians and investigators) | Yes | **Supplementary Materials** |
| Search strategy, including time period included in the synthesis and keywords | Yes | Supplementary Tables S1-S2 |
| Effort to include all available studies, including contact with authors | Yes | Supplementary Materials |
| Databases and registries searched | Yes | Pg 16 and Supplementary Materials |
| Search software used, name and version, including special features used (eg, explosion) | Yes | Supplementary Materials |
| Use of hand searching (eg, reference lists of obtained articles) | NA | Only transcriptomic datasets from GEO were included; no manual reference-based search was conducted as this review focused exclusively on public gene expression repositories. |
| List of citations located and those excluded, including justification | Yes | **Main manuscript**: Pp 26 and 27 (Methods – Dataset Selection; **Supplementary Information**: Pages **2–3** (Supplementary Methods – Dataset Retrieval and Processing); Tables S4-S8 |
| Method for addressing articles published in languages other than English | Yes | **Supplementary Information**: Page **2** (Supplementary Methods – Literature Search and Inclusion Criteria) |
| Method of handling abstracts and unpublished studies | Yes | **Main manuscript**: Page **26** (“Study Selection and Data Extraction”); **Supplementary Information**: Page **2–3** (“Supplementary Methods – Study Identification and Inclusion”) |
| Description of any contact with authors | Yes | Supplementary Methods |

# Reporting of Methods

| **Reporting Criteria** | **Reported (Yes/No)** | **Reported on Page No.** |
| --- | --- | --- |
| Description of relevance or appropriateness of studies assembled for assessing the hypothesis to be tested | Yes | **Main manuscript**: Page **27** (Material and Methods – subsección Data Processing and Differential Expression Analysis); **Supplementary Information**: Page **3** (Figure S1: Study selection workflow) |
| Rationale for the selection and coding of data (eg, sound clinical principles or convenience) | Yes | **Main manuscript**: Pages **26 and 27** (Material and Methods – subsección Dataset selection and raw data processing); **Supplementary Information**: Page **3** (Figure S1: Study selection workflow) |
| Documentation of how data were classified and coded (eg, multiple raters, blinding, and interrater reliability) | Yes | **Main manuscript**: Page **26** (Material and Methods – subsección Study Selection and Data Extraction); **Supplementary Information**: Pages **3–4** (Figure S1 and Table S1: Data processing pipeline) |
| Assessment of confounding (eg, comparability of cases and controls in studies where appropriate) | Yes | **Main manuscript**: Page 26 (Material and Methods – subsección *Risk of Bias and Dataset Quality*); **Supplementary Information**: Page **4** (Table S2) |
| Assessment of study quality, including blinding of quality assessors; stratification or regression on possible predictors of study results | Yes (partially) | **Main manuscript**: Pag 26 (*Risk of Bias and Dataset Quality*); **Supplementary Information**: Pages **2–3**, Table S1 |
| Assessment of heterogeneity | Yes | **Supplementary Methods**: Pages **4–5** (evaluación de heterogeneidad mediante análisis Leave-One-Study-Out [LOSO] y metafor::rma() para puntuaciones GSVA) |
| Description of statistical methods (eg, complete description of fixed or random effects models...) | Yes | (Main document) **page 27 Material and Methods – subsección *Meta-Analysis, Deconvolution and Robustness***, supplemented by **Supplementary Methods**, pages **3–6** (model specifications, heterogeneity management, limma + duplicateCorrelation, leave-one-study-out, GSVA, meta-analysis with metafor, etc.) |
| Provision of appropriate tables and graphics | Yes | Main Figures 1–7; Table 1; Supplementary Figures S1–S13 (pages 9–14, 17–22, 45–57) |

# Reporting of Results

| **Reporting Criteria** | **Reported (Yes/No)** | **Reported on Page No.** |
| --- | --- | --- |
| Table giving descriptive information for each study included | Yes | Table 1 (pp. 40-42) |
| Results of sensitivity testing (eg, subgroup analysis) | Yes | Supplementary Figures (pp. 40-42) |
| Indication of statistical uncertainty of findings | Yes | Supplementary Figures S12–S13, pp. 20–22 |

# Reporting of Discussion

| **Reporting Criteria** | **Reported (Yes/No)** | **Reported on Page No.** |
| --- | --- | --- |
| Quantitative assessment of bias (eg, publication bias) | No | All datasets were retrieved from public repositories (e.g., GEO) regardless of publication status, ensuring inclusion was independent of journal publication. As studies without deposited raw data cannot be identified or quantified, formal assessment of publication bias was not feasible. However, both published and unpublished datasets were explicitly noted. |
| Justification for exclusion (eg, exclusion of non–English-language citations) | Yes | Supp. Material: Tables S5-S8 (pg. 11-29) |
| Assessment of quality of included studies | Yes | Supp. Material: pgs. 101-104 |

# Reporting of Conclusions

| **Reporting Criteria** | **Reported (Yes/No)** | **Reported on Page No.** |
| --- | --- | --- |
| Consideration of alternative explanations for observed results | Yes | 20-24 |
| Generalization of the conclusions | Yes | 20-24 |
| Guidelines for future research | Yes | 24 |

# Disclosure of funding source

| **Reporting Criteria** | **Reported (Yes/No)** | **Reported on Page No.** |
| --- | --- | --- |
| Disclosure of funding source | Yes | 29, Supp. Material: pg 103, 104 |
