# Supplementary material for: Unravelling the transcriptomic landscape of primary lymphocytic scarring alopecias: systematic review and meta-analysis
Source: Front Immunol. 2025 Aug 11;16:1651019. doi: 10.3389/fimmu.2025.1651019 (PMC12375577; doi:10.3389/fimmu.2025.1651019)
Supplement: Supplementary file 2 [file Supplementaryfile2.docx]

| **Section and Topic** | **Item #** | **Checklist item** | **Location where item is reported** |
| --- | --- | --- | --- |
| **TITLE** | | |  |
| Title | 1 | Identify the report as a systematic review. | Page 1 (Title) |
| **ABSTRACT** | | |  |
| Abstract | 2 | See the PRISMA 2020 for Abstracts checklist. | Pages 7-8 (Unstructured abstract) |
| **INTRODUCTION** | | |  |
| Rationale | 3 | Describe the rationale for the review in the context of existing knowledge. | Pages 9-10 (Introduction) |
| Objectives | 4 | Provide an explicit statement of the objective(s) or question(s) the review addresses. | Page 10 (Introduction, last paragraph) |
| **METHODS** | | |  |
| Eligibility criteria | 5 | Specify the inclusion and exclusion criteria for the review and how studies were grouped for the syntheses. | Main manuscript: Page 26 ("Dataset selection and inclusion criteria") Supplementary Information: Pages 2–3 ("Supplementary Methods – Study Identification and Inclusion") |
| Information sources | 6 | Specify all databases, registers, websites, organisations, reference lists and other sources searched or consulted to identify studies. Specify the date when each source was last searched or consulted. | Main manuscript: Page 26 ("Dataset selection and raw data processing") Supplementary Information: Page 2 ("Supplementary Methods – Dataset Retrieval") |
| Search strategy | 7 | Present the full search strategies for all databases, registers and websites, including any filters and limits used. | **Location where item is reported**: Supplementary Tables S1–S2 (detailed search strategy and dataset accession numbers) |
| Selection process | 8 | Specify the methods used to decide whether a study met the inclusion criteria of the review, including how many reviewers screened each record and each report retrieved, whether they worked independently, and if applicable, details of automation tools used in the process. | Main manuscript: Page 26 ("Dataset selection and inclusion criteria") Supplementary Information: Page 2 |
| Data collection process | 9 | Specify the methods used to collect data from reports, including how many reviewers collected data from each report, whether they worked independently, any processes for obtaining or confirming data from study investigators, and if applicable, details of automation tools used in the process. | Main manuscript: Page 26 (last paragraph of "Dataset selection and raw data processing") Supplementary Information: Page 2 |
| Data items | 10a | List and define all outcomes for which data were sought. Specify whether all results that were compatible with each outcome domain in each study were sought (e.g. for all measures, time points, analyses), and if not, the methods used to decide which results to collect. | Main manuscript: Page 27 ("Raw data processing and DEG detection") Supplementary Information: Pages 3–4 |
|  | 10b | List and define all other variables for which data were sought (e.g. participant and intervention characteristics, funding sources). Describe any assumptions made about any missing or unclear information. | Supplementary Information: Page 3 (e.g., batch, platform, group annotation) |
| Study risk of bias assessment | 11 | Specify the methods used to assess risk of bias in the included studies, including details of the tool(s) used, how many reviewers assessed each study and whether they worked independently, and if applicable, details of automation tools used in the process. | Main manuscript: Pages 26 and 27 ("Risk of Bias and Dataset Quality") Supplementary Information: Page 4; Supplementary Table S3 |
| Effect measures | 12 | Specify for each outcome the effect measure(s) (e.g. risk ratio, mean difference) used in the synthesis or presentation of results. | Main manuscript: Pages 27 (DESeq2 log2 fold change, limma-derived effect size estimates for meta-analysis) |
| Synthesis methods | 13a | Describe the processes used to decide which studies were eligible for each synthesis (e.g. tabulating the study intervention characteristics and comparing against the planned groups for each synthesis (item #5)). | Supplementary Information |
|  | 13b | Describe any methods required to prepare the data for presentation or synthesis, such as handling of missing summary statistics, or data conversions. | Supplementary Information |
|  | 13c | Describe any methods used to tabulate or visually display results of individual studies and syntheses. | Main manuscript: Pages 26-28; Figures 1–7 Supplementary Figures S1–S25; Supplementary Tables S4–S9 |
|  | 13d | Describe any methods used to synthesize results and provide a rationale for the choice(s). If meta-analysis was performed, describe the model(s), method(s) to identify the presence and extent of statistical heterogeneity, and software package(s) used. | Main manuscript: Pages 26 and 27 ("Meta-analysis model", "limma + duplicateCorrelation") Supplementary Information: Pages 3–5 |
|  | 13e | Describe any methods used to explore possible causes of heterogeneity among study results (e.g. subgroup analysis, meta-regression). | Supplementary Information Supplementary Figures S3,S4.1-S4.2 |
|  | 13f | Describe any sensitivity analyses conducted to assess robustness of the synthesized results. | Supplementary Information Supplementary Figures S19–S22 |
| Reporting bias assessment | 14 | Describe any methods used to assess risk of bias due to missing results in a synthesis (arising from reporting biases). | Supplementary Information Supplementary Information: Pages 4–5. |
| Certainty assessment | 15 | Describe any methods used to assess certainty (or confidence) in the body of evidence for an outcome. | Supplementary Information: Pages 4–5 (Supplementary Methods – Risk of Bias Assessment); Main manuscript: Page 4 (Material and Methods – “Risk of bias and quality assessment”). |
| **RESULTS** | | |  |
| Study selection | 16a | Describe the results of the search and selection process, from the number of records identified in the search to the number of studies included in the review, ideally using a flow diagram. | Main manuscript (Figure 1, page 11); Supplementary Information (pg 2) |
|  | 16b | Cite studies that might appear to meet the inclusion criteria, but which were excluded, and explain why they were excluded. | Main manuscript (Figure 1, page 11); Supplementary Information (pg 2) |
| Study characteristics | 17 | Cite each included study and present its characteristics. | Supplementary Table S3; Main manuscript (Results – Dataset Characteristics, page 8) |
| Risk of bias in studies | 18 | Present assessments of risk of bias for each included study. | Supplementary Table S3; Main manuscript (Results – Dataset Characteristics, page 8) |
| Results of individual studies | 19 | For all outcomes, present, for each study: (a) summary statistics for each group (where appropriate) and (b) an effect estimate and its precision (e.g. confidence/credible interval), ideally using structured tables or plots. | Supplementary Figures S5–S8 (Forest plots); Supplementary Tables S7–S10 |
| Results of syntheses | 20a | For each synthesis, briefly summarise the characteristics and risk of bias among contributing studies. | Supplementary Figures S5–S8 (Forest plots); Supplementary Tables S7–S10 |
|  | 20b | Present results of all statistical syntheses conducted. If meta-analysis was done, present for each the summary estimate and its precision (e.g. confidence/credible interval) and measures of statistical heterogeneity. If comparing groups, describe the direction of the effect. | Supplementary Figures S5–S8 (CI 95%), Supplementary Tables S6–S10 |
|  | 20c | Present results of all investigations of possible causes of heterogeneity among study results. | Main manuscript (Results – Subtype-Specific Mechanisms, page 10); Supplementary Table S9 |
|  | 20d | Present results of all sensitivity analyses conducted to assess the robustness of the synthesized results. | Main manuscript (Results – Sensitivity Analyses, page 10); Supplementary Figures S9–S11 |
| Reporting biases | 21 | Present assessments of risk of bias due to missing results (arising from reporting biases) for each synthesis assessed. | Main manuscript (Discussion – Bias Considerations, page 11); Supplementary Methods (pg 5) |
| Certainty of evidence | 22 | Present assessments of certainty (or confidence) in the body of evidence for each outcome assessed. | Main manuscript (Discussion – Limitations and Strengths, page 12) |
| **DISCUSSION** | | |  |
| Discussion | 23a | Provide a general interpretation of the results in the context of other evidence. | **Main manuscript**, Discussion (pp. 21–24) – Integrates results with prior studies including Bao et al., Dubin et al., etc. |
|  | 23b | Discuss any limitations of the evidence included in the review. | **Main manuscript**, Strengths and Limitations (pp. 23 and 24) – Addresses metadata gaps, bulk transcriptomics, pooled controls. |
|  | 23c | Discuss any limitations of the review processes used. | **Main manuscript**, Strengths and Limitations (p. 23 and 24) – Notes platform differences, inclusion of unpublished datasets. |
|  | 23d | Discuss implications of the results for practice, policy, and future research. | **Main manuscript**, Conclusion (pp. 25) – Emphasises clinical translation, systemic surveillance, precision therapy design. |
| **OTHER INFORMATION** | | |  |
| Registration and protocol | 24a | Provide registration information for the review, including register name and registration number, or state that the review was not registered. | **Abstract, Main manuscript**, Methods (p. 26); also **Supplementary Information**, p. 2. Registered in PROSPERO: CRD42024559969. |
|  | 24b | Indicate where the review protocol can be accessed, or state that a protocol was not prepared. | **Main manuscript**, Methods (p. 26); **Supplementary Information**, p. 2. |
|  | 24c | Describe and explain any amendments to information provided at registration or in the protocol. | **Supplementary Information**, p. 2, “Protocol Deviations” section – minor clarifications; no major amendments to objectives or methods. |
| Support | 25 | Describe sources of financial or non-financial support for the review, and the role of the funders or sponsors in the review. | **Main manuscript**, **ACKNOWLEDGMENTS** section (p. 30) – Supported by ISCIII PI23/01590 (JR); no funder involvement in design, analysis, or publication. |
| Competing interests | 26 | Declare any competing interests of review authors. | **Main manuscript**, Conflict of Interest section (pp. 30 and 31) – |
| Availability of data, code and other materials | 27 | Report which of the following are publicly available and where they can be found: template data collection forms; data extracted from included studies; data used for all analyses; analytic code; any other materials used in the review. | **Supplementary Information** |

*From:*  Page MJ, McKenzie JE, Bossuyt PM, Boutron I, Hoffmann TC, Mulrow CD, et al. The PRISMA 2020 statement: an updated guideline for reporting systematic reviews. BMJ 2021;372:n71. doi: 10.1136/bmj.n71. This work is licensed under CC BY 4.0. To view a copy of this license, visit <https://creativecommons.org/licenses/by/4.0/>
