# Supplementary material for: Unravelling the transcriptomic landscape of primary lymphocytic scarring alopecias: systematic review and meta-analysis
Source: Front Immunol. 2025 Aug 11;16:1651019. doi: 10.3389/fimmu.2025.1651019 (PMC12375577; doi:10.3389/fimmu.2025.1651019)
Supplement: Supplementary file 3 [file DataSheet1.docx]

**Supplementary Information**

**Unravelling the Transcriptomic Landscape of Primary Lymphocytic Scarring Alopecias: Systematic Review and Meta-Analysis**

Irene Rivera-Ruiz^1,2,#^, Benjamin Ungar^3,#^, Viviana Dávila-Flores^4^, Jesús Gay-Mimbrera^1^, Pedro J. Gómez-Arias^1,2^, Miguel Juan-Cencerrado^1,2^, Carmen Mochón-Jiménez^1,2^, Esmeralda Parra-Peralbo^5^, Beatriz Isla-Tejera^3^, Teresa López-Viñau López^3,*^, Emma Guttman-Yassky^3,7^, Juan Ruano^1,2,*^

^1^: Inflammatory Immune-Mediated Chronic Skin Diseases Laboratory, IMIBIC, 14004 Córdoba, Spain

^2^: Department of Dermatology, Reina Sofía University Hospital, 14004 Córdoba, Spain

^3^: Department of Dermatology, Icahn School of Medicine at Mount Sinai, New York, New York.

^4^: Department of Pathology, Reina Sofía University Hospital, 14004 Córdoba, Spain

^5^: Department of Pharmacy and Nutrition, Faculty of Biomedical Science and Health, Universidad Europea, 28670 Madrid, Spain

^6^: Department of Pharmacy, Reina Sofía University Hospital, 14004 Córdoba, Spain

^7^: Laboratory of Inflammatory Skin Diseases, Icahn School of Medicine at Mount Sinai Hospital, New York, NY, USA

**Running title:** Transcriptome Meta-Analysis of Scarring Alopecias

0. Index

| **Supplementary Method Section** | **Title** | **Page** |
| --- | --- | --- |
| S1 | Search Strategy and Screening Results | 1 |
| S2 | Study Inclusion, Data Extraction, and Risk of Bias Assessment | 5 |
| S3 | Study-Specific Highlights and Contextual Summaries | 6 |
| S4 | Processing and Harmonization of Included Datasets | 7 |
| S5 | Data Availability, Normalization Pipelines, and Analytical Plan by Dataset | 8 |
| S6 | Differential Gene Expression and Functional Analysis | 8 |
| S7 | Meta-analysis of GSVA Functional Pathways Using a Leave-One-Study-Out Framework | 9 |
| S8 | Differential Abundance of Inferred Cell Types across Scarring Alopecia Subtypes | 10 |
| S9 | Sensitivity of Functional Enrichment Across Studies and Risk of Bias Assessment | 10 |
| S10 | Deviation from Protocol | 11 |
| S11 | Assessment of Study-Level Bias and Conflicts of Interest | 11 |
|  |  |  |
| **Supplementary Tables** | **Title** | **Page** |
| Table S1 | Summary of Records Retrieved by Search Strategy | 1 |
| Table S2 | Study Inclusion, Data Extraction, and Risk of Bias Assessment | 5 |
| Table S3 | Study-Specific Highlights and Contextual Summaries | 6 |
| Table S4 | Processing and Harmonization of Included Datasets | 7 |
| Table S5 | Data Availability, Normalization Pipelines, and Analytical Plan by Dataset | 8 |
| Table S6 | Differential Gene Expression and Functional Analysis | 8 |
| Table S7 | Meta-analysis of GSVA Functional Pathways Using a Leave-One-Study-Out Framework | 9 |
| Table S8 | Differential Abundance of Inferred Cell Types across Scarring Alopecia Subtypes | 10 |
| Table S9 | Sensitivity of Functional Enrichment Across Studies and Risk of Bias Assessment | 10 |
| Table S10 | Deviation from Protocol | 11 |
| Table S11 | Assessment of Study-Level Bias and Conflicts of Interest | 11 |
| **Supplementary Figures** | **Title** | **Page** |
| Fig. S1 | Hierarchical clustering of transcriptomes before batch correction | 1 |
| Fig. S2 | PCA before and after batch correction | 5 |
| Fig. S3 | Expression of most robust and most sensitive genes (LOSO analysis) | 6 |
| Fig. S4.1 | LOSO meta-analysis sensitivity: differentially expressed genes (panel 1) | 7 |
| Fig. S4.2 | LOSO meta-analysis sensitivity: differentially expressed genes (panel 2) | 8 |
| Fig. S5 | Network modules of subtype-specific DEGs: STRING-based clustering and annotation | 8 |

### ****S1. Search Strategy and Screening Results****

The search and dataset screening were conducted by the research team (JR and co-authors), consisting of dermatologists and clinician-scientists with substantial experience in systematic reviews, transcriptomic data analysis, and alopecia research. All investigators had previously published work in transcriptomic profiling of inflammatory dermatoses and meta-analytic methods, ensuring adequate expertise for dataset identification and assessment.

Two reviewers (IRR and FGG) independently screened all titles and abstracts retrieved through the systematic search to identify studies meeting the predefined inclusion criteria. Full-text articles were obtained for all potentially eligible records. Any discrepancies were resolved by consensus or consultation with a third reviewer (JR).

A total of **1,089 records** were identified across **seven data sources**. GEO and ArrayExpress contributed transcriptomic datasets (7 and 940 records, respectively). Additional studies were retrieved from PubMed (n = 11), Scopus (n = 27), the Cochrane Library (n = 5), ClinicalTrials.gov (n = 56), and grey literature sources (n = 43). After title/abstract screening and full-text review, most records were excluded due to reasons such as **non-scarring alopecia**, **lack of transcriptomic data**, or **unpublished results**.

**These findings highlight the importance of data availability and transparency in transcriptomic research. They also underscore the challenges in meta-analytical efforts involving rare diseases like scarring alopecias, where sample size is limited and data sharing is inconsistent across platforms and publications.**

****Supplementary Table S**1. Databases Searched, Dates, Results, and Strategies Used for Dataset Identification.** Summary of databases and grey literature sources searched for transcriptomic studies in scarring alopecias; searches were conducted in March 2024 according to the PROSPERO-registered protocol (CRD42024559969); queries were tailored to each database’s syntax and included terms related to primary lymphocytic scarring alopecias and gene expression profiling; filters were applied to limit results to human studies using transcriptomic platforms such as microarrays or RNA-seq; abbreviations: GEO, Gene Expression Omnibus; EMBL-EBI, European Molecular Biology Laboratory–European Bioinformatics Institute; RNA-seq, RNA sequencing; MeSH, Medical Subject Headings; BASE, Bielefeld Academic Search Engine; all retrieved records were screened according to predefined eligibility criteria as detailed in the Methods section.

| **Database** | **Date Searched** | **Results Retrieved** | **Search Strategy** |
| --- | --- | --- | --- |
| **GEO (NCBI)** | 23 March 2024 | 7 | ("alopecia cicatricial" OR "lichen planopilaris" OR "frontal fibrosing alopecia" OR "central centrifugal cicatricial alopecia" OR "pseudopelade de Brocq") AND ("skin"[MeSH Terms] OR "scalp") AND ("expression profiling by array" OR "expression profiling by high-throughput sequencing" OR "single cell") |
| **ArrayExpress (EMBL-EBI)** | 23 March 2024 | 940 | Key terms: “alopecia cicatricial,” “lichen planopilaris,” “frontal fibrosing alopecia,” “central centrifugal cicatricial alopecia,” and “pseudopelade de Brocq.” Filters included Homo sapiens as the organism, and either “transcription profiling by array” or “RNA-seq of coding RNA” as the assay type. Searches were limited to datasets released between 2000 and 2024. |
| **PubMed / MEDLINE (via Ovid)** | 23 March 2024 | 11 | ("lichen planopilaris" OR "frontal fibrosing alopecia" OR "central centrifugal cicatricial alopecia" OR "pseudopelade de Brocq" OR "scarring alopecia") AND ("gene expression" OR "transcriptomic" OR "RNA-seq" OR "microarray") AND ("human" OR "scalp" OR "lesional skin"); **Filters applied: English, Humans, Publication dates from 2000–2024** |
| **Scopus** | 23 March 2024 | 27 | **TITLE-ABS-KEY ("lichen planopilaris" OR "frontal fibrosing alopecia" OR "central centrifugal cicatricial alopecia" OR "pseudopelade de Brocq" OR "scarring alopecia") AND ("gene expression" OR transcriptomic OR RNA-seq OR microarray) AND (human OR scalp OR "lesional skin") AND (PUBYEAR > 1999 AND PUBYEAR < 2024)** |
| **Cochrane Library (Trials and Reviews)** | 23 March 2024 | 5 | "scarring alopecia" OR "lichen planopilaris" OR "frontal fibrosing alopecia" OR "central centrifugal cicatricial alopecia" in Title/Abstract; limited to trials and reviews mentioning transcriptomics |
| **Clinical Trials** | 23 March 2024 | 56 | AREA[ConditionSearch](alopecia) AND AREA[BasicSearch](Scarring Alopecia) |
| **Grey Literature (BASE, OpenAIRE, DART-Europe, ProQuest Dissertations & Theses)** | 23–25 March 2024 | 43 | A grey literature search was conducted using multiple sources, including BASE, OpenAIRE, DART-Europe, ProQuest Dissertations & Theses, and institutional repositories. Search terms included combinations of “scarring alopecia”, “lichen planopilaris”, “gene expression”, “transcriptomic”, and “RNA-seq”. Filters were applied to restrict results to human studies, theses, reports, and datasets published between 2000 and 2025. |

**Supplementary Table S2. Full Search Strategy in Ovid MEDLINE.** Search conducted on 1 April 2024. Complete Ovid MEDLINE search strategy used to identify transcriptomic studies involving primary scarring alopecias. The search combined controlled vocabulary and free-text terms for disease entities, transcriptomic technologies, and anatomical site. Filters were applied to restrict results to human studies published since the year 2000.

| **#** | **Query** | **Results** |
| --- | --- | --- |
| 1 | lichen planopilaris.mp. | 523 |
| 2 | frontal fibrosing alopecia.mp. | 507 |
| 3 | central centrifugal cicatricial alopecia.mp. | 141 |
| 4 | pseudopelade.mp. | 102 |
| 5 | 1 or 2 or 3 or 4 | 1036 |
| 6 | gene expression.mp. | 1237206 |
| 7 | transcriptomic*.mp. | 67605 |
| 8 | RNA-seq.mp. | 48502 |
| 9 | microarray.mp. | 99826 |
| 10 | single cell.mp. | 77826 |
| 11 | 6 or 7 or 8 or 9 or 10 | 1372015 |
| 12 | scalp.mp. | 39800 |
| 13 | skin.mp. | 855255 |
| 14 | 12 or 13 | 883393 |
| 15 | 5 and 11 and 14 | 11 |
| 16 | limit 15 to (humans and yr="2000 -Current") | 11 |

**Supplementary Table S3. Summary of Records Retrieved, Excluded, and Percentage Excluded by Database. Number of records retrieved per database, the number of studies excluded following full-text evaluation, and the corresponding percentage of excluded records.**

| **Database** | **Records Retrieved** | **Studies Excluded** | **% Excluded** |
| --- | --- | --- | --- |
| GEO | 7 | 1 | 14.3% |
| ArrayExpress | 940 | 937 | 99.7% |
| Ovid/MEDLINE | 11 | 10 | 90.9% |
| Scopus | 27 | 26 | 96.3% |
| Cochrane Library | 5 | 5 | 100.0% |
| ClinicalTrials.gov | 56 | 56 | 100.0% |
| Grey Literature | 43 | 43 | 100.0% |

# ****Supplementary Table S4. Reasons for Study Exclusion by Database****

**Number of excluded studies categorized by reason, displayed across databases. Each cell indicates the frequency of a specific exclusion reason within each source.**

| **Reason** | **ClinicalTrials** | **Cochrane**  **Library** | **Ovid/MEDLINE** | **Scopus** | **Total Excluded** |
| --- | --- | --- | --- | --- | --- |
| Animal model | 0 | 0 | 0 | 21 | **21** |
| Non-scarring alopecia | 20 | 0 | 0 | 0 | **20** |
| No transcriptomic data available | 18 | 0 | 0 | 0 | **18** |
| Results not published | 18 | 0 | 0 | 0 | **18** |
| Irrelevant topic | 0 | 0 | 0 | 4 | **4** |
| No transcriptomic data | 0 | 1 | 1 | 1 | **3** |
| Androgenetic alopecia | 0 | 2 | 0 | 0 | **2** |
| Animal study | 0 | 2 | 0 | 0 | **2** |
| Clinical study; no gene expression profiling | 0 | 0 | 2 | 0 | **2** |
| Single-cell study | 0 | 0 | 1 | 0 | **1** |
| Perspective article | 0 | 0 | 1 | 0 | **1** |
| No molecular profiling | 0 | 0 | 1 | 0 | **1** |
| Narrative review | 0 | 0 | 1 | 0 | **1** |
| Microdissected samples | 0 | 0 | 1 | 0 | **1** |
| Genetic association | 0 | 0 | 1 | 0 | **1** |
| miRNA study | 0 | 0 | 1 | 0 | **1** |

# **Supplementary Table S**5. Excluded Studies Identified in Ovid MEDLINE and Reasons for Exclusion

List of studies identified in Ovid MEDLINE that were excluded from the systematic review and meta-analysis, along with detailed justifications based on pre-established inclusion/exclusion criteria. Only one study (Karnik et al., 2009) met the criteria for inclusion and was integrated into the meta-analysis.

| **#** | **Citation** | **Decision** | **Reason for Exclusion** |
| --- | --- | --- | --- |
| 1 | Tziotzios C, Petridis C, Dand N, et al. Exp Dermatol. 2021;30(8):1154-1162. | Exclude | Narrative review; no original transcriptomic data. |
| 2 | Zirwas MJ, Singh A, Burney W, et al. Int J Dermatol. 2016;55(3):e143-e148. | Exclude | Clinical study focused on biopsy diagnostics; no gene expression profiling. |
| 3 | Tziotzios C, Ainali C, Holmes S, et al. J Invest Dermatol. 2017;137(11):2440–2443. | Exclude | Study based on miRNA, not mRNA transcriptomics. |
| 4 | Famenini S, Goh C. Clin Cosmet Investig Dermatol. 2015;8:333-344. | Exclude | Review of clinical features and treatment; no transcriptomic analysis. |
| 5 | Ladizinski B, Bazakas A, Selim MA, et al. J Am Acad Dermatol. 2013;68(5):749-755. | Exclude | Retrospective clinical study; no gene expression profiling. |
| 6 | Karnik P, Tekeste Z, McCormick TS, et al. J Invest Dermatol. 2009;129(5):1243-1257. | Include | Includes gene expression profiling relevant to scarring alopecia. |
| 7 | Gadre A, Gadkari R, Ward S, et al. J Invest Dermatol. 2023;143(12):2380-2391. | Exclude | Single-cell transcriptomic study; excluded by protocol. |
| 8 | Harries MJ, Meyer K, Chaudhry I, et al. J Pathol. 2013;231(2):236-247. | Exclude | Transcriptomic data restricted to microdissected stem cells; limited representation. |
| 9 | Vano-Galvan S, Molina-Ruiz AM, Arias-Santiago S, et al. J Am Acad Dermatol. 2014;70(4):670-678. | Exclude | Descriptive multicenter clinical study; no molecular profiling. |
| 10 | Vañó-Galván S, Saceda-Corralo D, Blume-Peytavi U, et al. Br J Dermatol. 2018;178(4):e276-e278. | Exclude | Genetic association study; does not include transcriptomic profiling. |
| 11 | Paus R, Bertolini M. J Investig Dermatol Symp Proc. 2015;17(2):9-10. | Exclude | Perspective article; no primary transcriptomic data. |

1. Tziotzios C, Petridis C, Dand N, et al. Frontal fibrosing alopecia: reflecting on the current understanding of pathogenesis and future directions. Exp Dermatol. 2021;30(8):1154–1162. doi:10.1111/exd.14340.
2. Zirwas MJ, Singh A, Burney W, et al. The diagnostic yield of biopsy in clinically suspected lichen planopilaris. Int J Dermatol. 2016;55(3):e143–e148. doi:10.1111/ijd.13029.
3. Tziotzios C, Ainali C, Holmes S, et al. Tissue and circulating microRNA co-expression analysis shows potential involvement of miRNAs in the pathobiology of frontal fibrosing alopecia. J Invest Dermatol. 2017;137(11):2440–2443. doi:10.1016/j.jid.2017.07.821.
4. Famenini S, Goh C. Central centrifugal cicatricial alopecia: challenges and solutions. Clin Cosmet Investig Dermatol. 2015;8:333–344. doi:10.2147/CCID.S63487.
5. Ladizinski B, Bazakas A, Selim MA, et al. Frontal fibrosing alopecia: a retrospective review of 19 patients seen at Duke University. J Am Acad Dermatol. 2013;68(5):749–755. doi:10.1016/j.jaad.2012.10.056.
6. Karnik P, Tekeste Z, McCormick TS, et al. Hair follicle stem cell-specific PPARgamma deletion causes scarring alopecia. J Invest Dermatol. 2009;129(5):1243–1257. doi:10.1038/jid.2008.339.
7. Gadre A, Gadkari R, Ward S, et al. A single-cell atlas of central centrifugal cicatricial alopecia reveals fibroinflammatory triggers of hair follicle degeneration. J Invest Dermatol. 2023;143(12):2380–2391. doi:10.1016/j.jid.2023.07.017.
8. Harries MJ, Meyer K, Chaudhry I, et al. Lichen planopilaris is characterized by immune privilege collapse of the hair follicle epithelial stem cell niche. J Pathol. 2013;231(2):236–247. doi:10.1002/path.4223.
9. Vano-Galvan S, Molina-Ruiz AM, Arias-Santiago S, et al. Frontal fibrosing alopecia: a multicenter review of 355 patients. J Am Acad Dermatol. 2014;70(4):670–678. doi:10.1016/j.jaad.2013.12.025.
10. Vañó-Galván S, Saceda-Corralo D, Blume-Peytavi U, et al. Frequency of the PADI3 genetic variant in central centrifugal cicatricial alopecia in a European cohort. Br J Dermatol. 2018;178(4):e276–e278. doi:10.1111/bjd.16235.
11. Paus R, Bertolini M. The role of hair follicle immune privilege collapse in scarring alopecia: lessons from lichen planopilaris. J Investig Dermatol Symp Proc. 2015;17(2):9–10. doi:10.1038/jidsymp.2015.7.

# Supplementary Table S6. Excluded Studies Identified in Scopus and Reasons for Exclusion.

This table lists the studies identified through the Scopus database that were excluded from the systematic review and meta-analysis. Each entry includes the citation, the exclusion decision, and the specific reason for exclusion based on predefined eligibility criteria. The majority of exclusions were due to the use of non-human (animal) models, lack of transcriptomic data, irrelevance to primary scarring alopecias, or focus on unrelated molecular techniques.

| **#** | **Citation** | **Decision** | **Reason for Exclusion** |
| --- | --- | --- | --- |
| 1 | Karnik P. et al. J Invest Dermatol. 2009;129(5):1243-1257. | Excluded | Animal model |
| 2 | Hong YK. et al. J Invest Dermatol. 2024;144(7):1491-1504.e10. | Excluded | Animal model |
| 3 | Méchin MC. et al. Int J Mol Sci. 2020;21(2):nan-nan. | Excluded | Animal model |
| 4 | Strobl K. et al. EMBO Mol Med. 2024;16(12):3142-3168. | Excluded | Animal model |
| 5 | Del Duca E. et al. Br J Dermatol. 2020;183(6):1083-1093. | Excluded | Animal model |
| 6 | Karami H. et al. IET Syst Biol. 2022;16(5):173-185. | Excluded | Irrelevant topic |
| 7 | Chacon-Camacho OF. et al. Am J Med Genet A. 2020;182(11):2773-2777. | Excluded | Animal model |
| 8 | Sidorova IV. et al. Reprod Endocrinol. 2020;52:39-48. | Excluded | Irrelevant topic |
| 9 | Nemer G. et al. Clin Genet. 2020;98(2):116-125. | Excluded | Irrelevant topic |
| 10 | Halley-Stott RP. et al. Stem Cell Rev Rep. 2020;16(6):1105-1120. | Excluded | Animal model |
| 11 | Villavisanis DF. et al. J Hand Surg. 2024;49(5):472-480. | Excluded | Animal model |
| 12 | Xiong J. et al. Bioact Mater. 2024;36:112-125. | Excluded | Animal model |
| 13 | Liu L. et al. Front Immunol. 2022;13:nan-nan. | Excluded | Animal model |
| 14 | Sardella C. et al. J Invest Dermatol. 2018;138(3):500-510. | Excluded | Animal model |
| 15 | Irurzun I. et al. Pediatr Dermatol. 2021;38(3):568-574. | Excluded | Animal model |
| 16 | Gaspar NK. An Bras Dermatol. 2016;91(6):776-780. | Excluded | Animal model |
| 17 | Yu M. et al. J Dermatol Sci. 2010;57(1):27-36. | Excluded | Animal model |
| 18 | Dubin C. et al. J Am Acad Dermatol. 2022;86(3):551-562. | Excluded | Irrelevant topic |
| 19 | Ohyama M. et al. Skin Res. 2012;11(Suppl 19):4-10. | Excluded | Animal model |
| 20 | Wood GA. et al. Exp Dermatol. 2005;14(5):373-379. | Excluded | Animal model |
| 21 | Bao A. et al. JAMA Dermatol. 2024;160(11):1211-1219. | Excluded | Animal model |
| 22 | Harries MJ, Paus R. J Invest Dermatol. 2009;129(5):1066-1070. | Excluded | Animal model |
| 23 | Li Q. et al. Lupus. 2022;31(11):1306-1316. | Excluded | Animal model |
| 24 | Shalbaf M. et al. Lupus Sci Med. 2019;6(1):nan-nan. | Excluded | Irrelevant topic |
| 25 | Hiroi A. et al. Br J Dermatol. 2006;155(2):437-445. | Excluded | Animal model |
| 26 | Rothnagel JA. et al. J Dermatol Sci. 1994;7(Suppl 1):S164-S169. | Excluded | Animal model |
| 27 | Gadre A. et al. JID Innov. 2024;4(3):nan-nan. | Excluded | Non-transcriptomic data |

Citation

1. Karnik P.; Tekeste Z.; McCormick T.S.; Gilliam A.C.; Price V.H.; Cooper K.D.; Mirmirani P.. Hair follicle stem cell-specific PPARγ deletion causes scarring alopecia. Journal of Investigative Dermatology. 2009;129.0(5):1243-1257
2. Hong Y.-K.; Hwang D.-Y.; Yang C.-C.; Cheng S.M.; Chen P.-C.; Aala W.J.; I-Chen Harn H.; Evans S.T.; Onoufriadis A.; Liu S.-L.; Lin Y.-C.; Chang Y.-H.; Lo T.-K.; Hung K.-S.; Lee Y.-C.; Tang M.-J.; Lu K.Q.; McGrath J.A.; Hsu C.-K.. Profibrotic Subsets of SPP1+ Macrophages and POSTN+ Fibroblasts Contribute to Fibrotic Scarring in Acne Keloidalis. Journal of Investigative Dermatology. 2024;144.0(7):1491-1504.e10
3. Méchin M.-C.; Takahara H.; Simon M.. Deimination and Peptidylarginine Deiminases in Skin Physiology and Diseases. Int J Mol Sci. 2020 Jan 15;21(2):566. doi: 10.3390/ijms21020566. PMID: 31952341; PMCID: PMC7014782.
4. Strobl K.; Klufa J.; Jin R.; Artner-Gent L.; Krauß D.; Novoszel P.; Strobl J.; Stary G.; Vujic I.; Griss J.; Holcmann M.; Farlik M.; Homey B.; Sibilia M.; Bauer T.. JAK-STAT1 as therapeutic target for EGFR deficiency-associated inflammation and scarring alopecia. EMBO Molecular Medicine. 2024;16.0(12):3142-3168
5. Del Duca E.; Ruano Ruiz J.; Pavel A.B.; Sanyal R.D.; Song T.; Gay-Mimbrera J.; Zhang N.; Estrada Y.D.; Peng X.; Renert-Yuval Y.; Phelps R.G.; Paus R.; Krueger J.G.; Guttman-Yassky E.. Frontal fibrosing alopecia shows robust T helper 1 and Janus kinase 3 skewing. British Journal of Dermatology. 2020;183.0(6):1083-1093
6. Karami H.; Nomiri S.; Ghasemigol M.; Mehrvarzian N.; Derakhshani A.; Fereidouni M.; Mirimoghaddam M.; Safarpour H.. CHAC1 as a novel biomarker for distinguishing alopecia from other dermatological diseases and determining its severity. IET Systems Biology. 2022;16.0(5):173-185
7. Chacon-Camacho O.F.; Arce-Gonzalez R.; Ordaz-Robles T.; Perezpeña-Diazconti M.; Nava-Castañeda A.; Zenteno J.C.. Exome sequencing identifies a SREBF1 recurrent ARG557CYS mutation as the cause of hereditary mucoepithelial dysplasia in a family with high clinical variability. American Journal of Medical Genetics, Part A. 2020;182.0(11):2773-2777
8. Sidorova I.V.; Tutchenko T.M.; Burka O.A.; Semeniuk L.M.; Nohovska I.G.; Demianenko L.V.. Androgen-associated and androgen-independent causes of alopecia and acne in women. Reproductive Endocrinology. 2020;nan(52):39-48
9. Nemer G.; El-Hachem N.; Eid E.; Hamie L.; Bardawil T.; Khalil S.; El-Rassy I.; Safi R.; Khalil A.; Abbas O.; Shimomura Y.; Kurban M.. A novel TRAF3IP2 variant causing familial scarring alopecia with mixed features of discoid lupus erythematosus and folliculitis decalvans. Clinical Genetics. 2020;98.0(2):116-125
10. Halley-Stott R.P.; Adeola H.A.; Khumalo N.P.. Destruction of the stem cell Niche, Pathogenesis and Promising Treatment Targets for Primary Scarring Alopecias. Stem Cell Reviews and Reports. 2020;16.0(6):1105-1120
11. Villavisanis D.F.; Perrault D.P.; Kiani S.N.; Cholok D.; Fox P.M.. Current Treatment Landscape for Dystrophic Epidermolysis Bullosa: From Surgical Management to Emerging Gene Therapies and Novel Skin Grafts. Journal of Hand Surgery. 2024;49.0(5):472-480
12. Xiong J.; Liu Z.; Jia L.; Sun Y.; Guo R.; Xi T.; Li Z.; Wu M.; Jiang H.; Li Y.. Bioinspired engineering ADSC nanovesicles thermosensitive hydrogel enhance autophagy of dermal papilla cells for androgenetic alopecia treatment. Bioactive Materials. 2024;36.0(nan):112-125
13. Liu L.; Chen Y.; Chen J.; Xue Y.; Chen T.; Li Y.; Shao X.; Chen J.. Association between frontal fibrosing Alopecia and Rosacea: Results from clinical observational studies and gene expression profiles. Frontiers in Immunology. 2022;13.0(nan):nan-nan
14. Sardella C.; Winkler C.; Quignodon L.; Hardman J.A.; Toffoli B.; Giordano Attianese G.M.P.; Hundt J.E.; Michalik L.; Vinson C.R.; Paus R.; Desvergne B.; Gilardi F.. Delayed Hair Follicle Morphogenesis and Hair Follicle Dystrophy in a Lipoatrophy Mouse Model of Pparg Total Deletion. Journal of Investigative Dermatology. 2018;138.0(3):500-510
15. Irurzun I.; Natale M.I.; Agostinelli M.L.; Lamberti M.; Montero D.; Granda C.; Mássimo J.A.; Manzur G.B.; Valinotto L.E.. Ichthyosis follicularis, atrichia and photophobia (IFAP) and hereditary mucoepithelial dysplasia: Two syndromes that share a common clinical spectrum. Pediatric Dermatology. 2021;38.0(3):568-574
16. Gaspar N.K.. DHEA and frontal fibrosing alopecia: Molecular and physiopathological mechanisms. Anais Brasileiros de Dermatologia. 2016;91.0(6):776-780
17. Yu M.; Bell R.H.; Ross E.K.; Lo B.K.K.; Isaac-Renton M.; Martinka M.; Haegert A.; Shapiro J.; McElwee K.J.. Lichen planopilaris and pseudopelade of Brocq involve distinct disease associated gene expression patterns by microarray. Journal of Dermatological Science. 2010;57.0(1):27-36
18. Dubin C.; Glickman J.W.; Del Duca E.; Chennareddy S.; Han J.; Dahabreh D.; Estrada Y.D.; Zhang N.; Kimmel G.W.; Singer G.; Chowdhury M.; Zheng A.Y.; Angelov M.; Gay-Mimbrera J.; Ruano Ruiz J.; Krueger J.G.; Pavel A.B.; Guttman-Yassky E.. Scalp and serum profiling of frontal fibrosing alopecia reveals scalp immune and fibrosis dysregulation with no systemic involvement. Journal of the American Academy of Dermatology. 2022;86.0(3):551-562
19. Ohyama M.; Kobayashi T.; Sakai N.; Veraitch O.; Amagai M.. Evaluation of the effects of roxithromycin on the intrinsic gene expression in canine hair follicle cells. Skin Research. 2012;11.0(SUPPL. 19):4-10
20. Wood G.A.; Flenniken A.; Osborne L.; Fleming C.; Vukobradovic I.; Morikawa L.; Xu Q.; Porter R.; Adamson S.L.; Rossant J.; McKerlie C.. Two mouse mutations mapped to chromosome 11 with differing morphologies but similar progressive inflammatory alopecia. Experimental Dermatology. 2005;14.0(5):373-379
21. Bao A.; Qadri A.; Gadre A.; Will E.; Collins D.; Ahima R.; Bordone L.A.; Aguh C.. Low-Dose Metformin and Profibrotic Signature in Central Centrifugal Cicatricial Alopecia. JAMA Dermatology. 2024;160.0(11):1211-1219
22. Harries M.J.; Paus R.. Scarring alopecia and the PPAR-γ connection. Journal of Investigative Dermatology. 2009;129.0(5):1066-1070
23. Li Q.; Yang M.; Chen K.; Zhou S.; Zhou S.; Wu H.. Tight correlation of 5-hydroxymethylcytosine expression with the scarring damage of discoid lupus erythematosus. Lupus. 2022;31.0(11):1306-1316
24. Shalbaf M.; Alase A.A.; Berekmeri A.; Md Yusof M.Y.; Pistolic J.; Goodfield M.J.; Edward S.; Botchkareva N.V.; Stacey M.; Vital E.M.; Wittmann M.. Plucked hair follicles from patients with chronic discoid lupus erythematosus show a disease-specific molecular signature. Lupus Science and Medicine. 2019;6.0(1):nan-nan
25. Hiroi A.; Ito T.; Seo N.; Uede K.; Yoshimasu T.; Ito M.; Nakamura K.; Ito N.; Paus R.; Furukawa F.. Male New Zealand Black/KN mice: A novel model for autoimmune-induced permanent alopecia?. British Journal of Dermatology. 2006;155.0(2):437-445
26. Rothnagel J.A.; Longley M.A.; Holder R.A.; Bundman D.S.; Seki T.; Bickenbach J.R.; Roop D.R.. Genetic disorders of keratin: are scarring alopecias a sub-set?. Journal of Dermatological Science. 1994;7.0(SUPPL. 1):S164-S169
27. Gadre A, Dyson T, Jedrych J, Anhalt G, Byrd AS, Aguh C. Proteomic Profiling of Central Centrifugal Cicatricial Alopecia Reveals Role of Humoral Immune Response Pathway and Metabolic Dysregulation. JID Innov. 2024 Jan 28;4(3):100263. doi: 10.1016/j.xjidi.2024.100263. Erratum in: JID Innov. 2024 Aug 30;4(6):100312. doi: 10.1016/j.xjidi.2024.100312. PMID: 38585195; PMCID: PMC10995914.

# Supplementary Table S7. Excluded Studies Identified in Cochrane Library and Reasons for Exclusion.

List of studies identified through the Cochrane Library database that were excluded from the systematic review and meta-analysis. Reasons include irrelevant conditions such as androgenetic alopecia, absence of transcriptomic data, and use of non-human models.

| **#** | **Citation** | **Inclusion** | **Reason for Exclusion** |
| --- | --- | --- | --- |
| 1 | De Villez RL. Topical minoxidil therapy in hereditary androgenetic alopecia. Arch Dermatol. 1985;121(2):197–202. | Exclude | Androgenetic alopecia |
| 2 | Keene S, Goren A. Genetic variations in the androgen receptor gene and finasteride response in women with androgenetic alopecia. Dermatol Ther. 2011;24(2):296–300. | Exclude | No transcriptomic data |
| 3 | Mirmirani P, Consolo M, Oyetakin-White P, et al. Similar response patterns to topical minoxidil foam 5% in men with androgenetic alopecia: a microarray analysis. Br J Dermatol. 2015;172(6):1555–1561. | Exclude | Androgenetic alopecia |
| 4 | Nakamura T, Yamamura H, Park K, et al. Chicken egg yolk peptides stimulate hair growth via VEGF production. J Med Food. 2018;21(7):701–708. | Exclude | Animal study |
| 5 | Tenore GC, Caruso D, Buonomo G, et al. Annurca apple nutraceutical promotes hair growth in clinical trial. J Med Food. 2018;21(1):90–103. | Exclude | Animal study |

1. De Villez RL. Topical minoxidil therapy in hereditary androgenetic alopecia. Arch Dermatol. 1985;121(2):197–202.
2. Keene S, Goren A. Genetic variations in the androgen receptor gene and finasteride response in women with androgenetic alopecia. Dermatol Ther. 2011;24(2):296–300.
3. Mirmirani P, Consolo M, Oyetakin-White P, et al. Similar response patterns to topical minoxidil foam 5% in men with androgenetic alopecia: a microarray analysis. Br J Dermatol. 2015;172(6):1555–1561.
4. Nakamura T, Yamamura H, Park K, et al. Chicken egg yolk peptides stimulate hair growth via VEGF production. J Med Food. 2018;21(7):701–708.
5. Tenore GC, Caruso D, Buonomo G, et al. Annurca apple nutraceutical promotes hair growth in clinical trial. J Med Food. 2018;21(1):90–103.

# Supplementary Table S8. Excluded Studies from ClinicalTrials.gov and Reasons for Exclusion

List of clinical trials identified through ClinicalTrials.gov that were excluded from the systematic review and meta-analysis. Exclusion reasons include studies on non-scarring alopecia, absence of alopecia as primary condition, lack of transcriptomic data, or unavailability of results.

|  | **Citation** | **Decision** | **Reason** |
| --- | --- | --- | --- |
| 1 | Use of Botulinum Toxin in the Treatment of Androgenic Alopecia (ClinicalTrials.gov ID: NCT05456087, Status: ACTIVE_NOT_RECRUITING) | Excluded | Non-scarring alopecia |
| 2 | Pilot Study of the Effect of Laser on Reversing Chronic Radiation Injury (ClinicalTrials.gov ID: NCT01910818, Status: ACTIVE_NOT_RECRUITING) | Excluded | No alopecia |
| 3 | The Effect of Platelet Rich Plasma on Non-scarring Alopecia (ClinicalTrials.gov ID: NCT03689452, Status: COMPLETED) | Excluded | Non-scarring alopecia |
| 4 | To Compare the Efficacy of Weekly Azathioprine Pulse Versus Betamethasone Oral Mini-Pulse in the Treatment of Moderate to Severe Alopecia Areata (ClinicalTrials.gov ID: NCT06786689, Status: COMPLETED) | Excluded | Non-scarring alopecia |
| 5 | New Treatment of Alopecia Areata (ClinicalTrials.gov ID: NCT05594316, Status: COMPLETED) | Excluded | Non-scarring alopecia |
| 6 | Healthcare Disparities in Alopecia Areata (ClinicalTrials.gov ID: NCT05727306, Status: COMPLETED) | Excluded | Non-scarring alopecia |
| 7 | Ritlecitinib for Cicatricial Alopecia (ClinicalTrials.gov ID: NCT05549934, Status: COMPLETED) | Excluded | Results not published; no accessible data for systematic review inclusion. |
| 8 | Evaluation of the Effect of Topical Calcipotriol Versus Platelet-Rich Plasma (ClinicalTrials.gov ID: NCT05954104, Status: COMPLETED) | Excluded | Non-scarring alopecia |
| 9 | Dual JAK1/TYK2 Inhibitor for Cicatricial Alopecia (ClinicalTrials.gov ID: NCT05076006, Status: COMPLETED) | Excluded | Results not published; no accessible data for systematic review inclusion. |
| 10 | Role of Neurogenic Inflammation and Topical 6% Gabapentin Therapy in Symptomatic Scarring Alopecia (ClinicalTrials.gov ID: NCT03346668, Status: COMPLETED) | Excluded | Results not published; no accessible data for systematic review inclusion. |
| 11 | Topical 5% Minoxidil and Potent Topical Corticosteroid Versus Intralesional Corticosteroid in the Treatment of Alopecia Areata (ClinicalTrials.gov ID: NCT03535233, Status: COMPLETED) | Excluded | Non-scarring alopecia |
| 12 | Barriers to Care and QOL for CCCA Patients (ClinicalTrials.gov ID: NCT03044782, Status: COMPLETED) | Excluded | No study results |
| 13 | Apremilast in the Treatment of Central Centrifugal Cicatricial Alopecia (CCCA) (ClinicalTrials.gov ID: NCT03521687, Status: COMPLETED) | Excluded | Results not published; no accessible data for systematic review inclusion. |
| 14 | Phototoxic Doses of Ultraviolet A for Treatment of Alopecia Areata (ClinicalTrials.gov ID: NCT01559584, Status: COMPLETED) | Excluded | Non-scarring alopecia |
| 15 | Quality of Life Comparison in Patients With Different Types of Alopecia (ClinicalTrials.gov ID: NCT04246957, Status: COMPLETED) | Excluded | Results not published; no accessible data for systematic review inclusion. |
| 16 | Expression of Fas Protein in Skin Biopsies of Participants With Scarring Alopecia (ClinicalTrials.gov ID: NCT00691769, Status: COMPLETED) | nan | Results not published; no accessible data for systematic review inclusion. |
| 17 | Hair Transplantation in Cicatricial Alopecia (ClinicalTrials.gov ID: NCT04472715, Status: COMPLETED) | nan | Results not published; no accessible data for systematic review inclusion. |
| 18 | Relevance of Trichoscopy in Differential Diagnosis of Focal Non-cicatricial Alopecia in Children (ClinicalTrials.gov ID: NCT03260777, Status: COMPLETED) | nan | Results not published; no accessible data for systematic review inclusion. |
| 19 | a Retrospective Study on the Systemic Treatment of LPP and FFA (ClinicalTrials.gov ID: NCT06512766, Status: COMPLETED) | nan | Results not published; no accessible data for systematic review inclusion. |
| 20 | A Study of Revian Red All LED Cap as a Novel Treatment for Central Centrifugal Cicatricial Alopecia (ClinicalTrials.gov ID: NCT05759338, Status: COMPLETED) | nan | Results not published; no accessible data for systematic review inclusion. |
| 21 | Keratinocyte Growth Factor- Hair Serum for the Prevention of Chemotherapy Induced Alopecia (ClinicalTrials.gov ID: NCT04554732, Status: COMPLETED) | nan | Results not published; no accessible data for systematic review inclusion. |
| 22 | Follicular Revival in Fibrosing Alopecia: Evaluating Use of Micro-needling (ClinicalTrials.gov ID: NCT04342091, Status: COMPLETED) | Excluded | No transcriptomic data available |
| 23 | Oral Low-Dose Naltrexone for Lichen Planopilaris and Frontal Fibrosing Alopecia (ClinicalTrials.gov ID: NCT04409041, Status: COMPLETED) | Excluded | No transcriptomic data available |
| 24 | A Study to Evaluate the Safety and Tolerability of Tirbanibulin Ointment 1% in Adult Participants With Actinic Keratosis (ClinicalTrials.gov ID: NCT05279131, Status: COMPLETED) | nan | Non alopecia |
| 25 | Efficacy Study of Cosmetic Product Against Telogen Effluvium on Women (ClinicalTrials.gov ID: NCT04652232, Status: COMPLETED) | Excluded | Non-scarring alopecia |
| 26 | Pain Outcomes Following Intralesional Corticosteroid Injections (ClinicalTrials.gov ID: NCT03630198, Status: COMPLETED) | Excluded | Non transcriptomic analysis |
| 27 | Study to Determine Effect of Gentle Wounding to Stimulate Hair Follicle Neogenesis (ClinicalTrials.gov ID: NCT03491267, Status: ENROLLING_BY_INVITATION) | Excluded | No alopecia |
| 28 | Methotrexate Versus Triamcinilone Acetonide in Treatment of Recalcitrant Alopecia Areata (ClinicalTrials.gov ID: NCT06088147, Status: NOT_YET_RECRUITING) | Excluded | Non-scarring alopecia |
| 29 | Treatment of Alopecic Scars by Follicular Unit Extraction Hair Transplantation (ClinicalTrials.gov ID: NCT06145672, Status: NOT_YET_RECRUITING) | Excluded | No transcriptomic data available |
| 30 | Evaluation of Microbiota Transplant Therapy in Patients With Alopecia Areata (ClinicalTrials.gov ID: NCT06747611, Status: NOT_YET_RECRUITING) | Excluded | Non-scarring alopecia |
| 31 | Topical Simvastatin Versus Topical Steroid in Treatment of Alopecia Areata (ClinicalTrials.gov ID: NCT06399783, Status: NOT_YET_RECRUITING) | Excluded | Non-scarring alopecia |
| 32 | Study on the Efficacy of Autologous Fat Grafting in Improving Hair Transplantation Outcomes for Patients With Localized Scleroderma-Related Alopecia (ClinicalTrials.gov ID: NCT06646146, Status: NOT_YET_RECRUITING) | Excluded | No transcriptomic data available |
| 33 | Evaluation of Serum Levels of Interlukin-15 and Interlukin-21 in Patients With Alopecia Areata (ClinicalTrials.gov ID: NCT05910138, Status: NOT_YET_RECRUITING) | Excluded | Non-scarring alopecia |
| 34 | Biocellular-Cellular Regenerative Treatment Scaring Alopecia and Alopecia Areata (ClinicalTrials.gov ID: NCT03078686, Status: RECRUITING) | Excluded | No transcriptomic data available |
| 35 | 1470nm Laser for the Treatment of Androgenetic Alopecia and Scarring Alopecia (ClinicalTrials.gov ID: NCT05460611, Status: RECRUITING) | Excluded | No transcriptomic data available |
| 36 | Quantification of the Socio-economic Multifacet Burden of Alopecia Areata and Identification of the Associated Factors (ClinicalTrials.gov ID: NCT05588310, Status: RECRUITING) | Excluded | Non-scarring alopecia |
| 37 | Polymorphism of Janus Kinase 1 and 2 (JAK 1&2) in Patients With Alopecia Areata (ClinicalTrials.gov ID: NCT05861401, Status: RECRUITING) | Excluded | Non-scarring alopecia |
| 38 | Combined Microneedling With Either 1% Lactic Acid Solution or Vitamin D3 or Triamcinolone Acetonide in The Treatment of Alopecia Areata (ClinicalTrials.gov ID: NCT06327581, Status: RECRUITING) | Excluded | Non-scarring alopecia |
| 39 | Azelaic Acid as a Novel Treatment for Central Centrifugal Cicatricial Alopecia (CCCA) (ClinicalTrials.gov ID: NCT05416333, Status: RECRUITING) | Excluded | No transcriptomic data available |
| 40 | A Pilot Study of Revian Red All LED Cap as a Novel Treatment for Central Centrifugal Cicatricial Alopecia (ClinicalTrials.gov ID: NCT04764331, Status: RECRUITING) | Excluded | Not available data yet |
| 41 | Treatment Results for Patients With Central Centrifugal Cicatricial Alopecia (CCCA): a Multicenter Prospective Study (ClinicalTrials.gov ID: NCT04207931, Status: RECRUITING) | Excluded | Not available data yet |
| 42 | The Effectiveness of Hydroxychloroquine Versus Methotrexate in the Treatment of Lichen Planopilaris in Routine Clinical Care: a Patient Preference Trial (ClinicalTrials.gov ID: NCT06512753, Status: RECRUITING) | Excluded | Non-scarring alopecia |
| 43 | Safety and Efficacy Evaluation of the Mosaic Ultra Device (ClinicalTrials.gov ID: NCT06117293, Status: RECRUITING) | Excluded | No alopecia |
| 44 | PAI-1 Expression in Non-scarring Hair Loss (ClinicalTrials.gov ID: NCT02548689, Status: TERMINATED) | Excluded | Non-scarring alopecia |
| 45 | Ocular Finding in Alopecia Areata (ClinicalTrials.gov ID: NCT03155958, Status: UNKNOWN) | Excluded | Non-scarring alopecia |
| 46 | Excimer Light and Topical Steroid in Treatment of Alopecia Areata (ClinicalTrials.gov ID: NCT04793945, Status: UNKNOWN) | Excluded | Non-scarring alopecia |
| 47 | Efficacy of Fractional CO2 Laser as a Mono- or Adjuvant Therapy for Alopecia Areata (ClinicalTrials.gov ID: NCT04003376, Status: UNKNOWN) | Excluded | Non-scarring alopecia |
| 48 | Efficacy of Combined Microneedling With Methotrexate in Treatment of Alopecia Areata (ClinicalTrials.gov ID: NCT05485571, Status: UNKNOWN) | Excluded | Non-scarring alopecia |
| 49 | Hair Regrowth After Bicoronal Incision (ClinicalTrials.gov ID: NCT01557491, Status: UNKNOWN) | Excluded | No transcriptomic data available |
| 50 | Hair Counts From Vertical and Horizontal Sections of Scalp Biopsy SPecimens in Thai Population With Alopecia (ClinicalTrials.gov ID: NCT01651689, Status: UNKNOWN) | Excluded | No transcriptomic data available |
| 51 | The Comparison Study of Intralesional Botulinum Toxin A and Corticosteroid Injection for Alopecia Areata (ClinicalTrials.gov ID: NCT00999869, Status: UNKNOWN) | Excluded | Non-scarring alopecia |
| 52 | Safety and Efficacy of Clobetasol Propionate 0.05% E Foam in Alopecia (ClinicalTrials.gov ID: NCT01111981, Status: UNKNOWN) | Excluded | No transcriptomic data available |
| 53 | Evaluating the Efficacy of Microneedling in the Treatment of Androgenetic Alopecia (ClinicalTrials.gov ID: NCT02154503, Status: UNKNOWN) | Excluded | Non-scarring alopecia |
| 54 | Split Wound Comparison of Trichophytic Closure Techniques for Donor Site in Hair Transplantation (ClinicalTrials.gov ID: NCT01655602, Status: UNKNOWN) | Excluded | Non-scarring alopecia |
| 55 | Study to Assess the Efficacy of Intralesional Corticosteroid on the Treatment of Frontal Fibrosing Alopecia (ClinicalTrials.gov ID: NCT02467101, Status: UNKNOWN) | Excluded | No transcriptomic data available |
| 56 | Calcipotriol as a Novel Treatment for Central Centrifugal Cicatricial Alopecia (CCCA) (ClinicalTrials.gov ID: NCT05416320, Status: WITHDRAWN) | Excluded | Study withdrawn |

**S2. Study Inclusion, and Data Extraction**

Data extraction was independently performed by two reviewers using a pre-defined template to ensure methodological consistency. Six transcriptomic datasets were selected for inclusion in the meta-analysis, encompassing RNA-seq and microarray platforms across primary scarring alopecia subtypes (FFA, LPP, CCCA, PsPB) and controls. A summary of dataset characteristics—including GEO accession numbers, platforms, sample composition, and type of data available—is provided in **Supplementary Table S8**. RNA-seq datasets were processed using DESeq2 or edgeR from raw counts, while Affymetrix microarray data were normalised using the RMA method via the affy or oligo packages. Datasets lacking CEL files or standard annotations (e.g., GSE11905) were excluded from cross-platform integration.

Dataset-specific technical limitations and preprocessing solutions are detailed in **Supplementary Table S9**. For example, in GSE186075, several samples were only available as FPKM matrices. To enable count-based differential expression analysis, the corresponding raw SRA files were retrieved and processed using Salmon to generate gene-level counts compatible with DESeq2.

**Supplementary Table S9**. **Experimental Platforms, File Availability, Data Type, and Processing Workflow by Dataset**. This table provides a technical summary of all datasets included in the meta-analysis, highlighting the experimental platform used, the types of files available in GEO, and the analytical strategy applied to each dataset. RNA-seq and microarray data were processed using standardized pipelines to enable downstream integration. Datasets lacking raw or standardized input files were excluded from integrative analyses but retained for qualitative comparisons when appropriate.

| **GEO Accession** | **Platform** | **Data in GEO** | **Analysis Plan** |
| --- | --- | --- | --- |
| GSE186075 | RNA-seq (Illumina HiSeq 3000) | Raw counts, FPKM, and SRR files | SRR files were converted to FASTQ and processed with Salmon; counts aggregated with tximport; merged and normalized using DESeq2 |
| GSE59131 | Microarray (Affymetrix U133 Plus 2.0) | .CEL files and normalized matrix available | CEL files processed using *affy::rma*; probes mapped with hgu133plus2.db |
| GSE58934 | Microarray (Affymetrix U133 Plus 2.0) | .CEL files and normalized matrix available | CEL files processed using *affy::rma*; probes mapped with hgu133plus2.db |
| GSE179054 | Microarray (Affymetrix Clariom S) | .CEL files and processed matrix available | Each chip type was processed separately with oligo::rma and merged by gene. |
| GSE113052 | Microarray (Affymetrix Clariom S) | .CEL files and processed matrix available | CEL files processed using *oligo::rma;* mapped via clariomshumantranscriptcluster.db |
| GSE125733 | RNA-seq (Illumina HiSeq 2000) | Raw counts and FPKM available | CEL files processed using *oligo::rma*; mapped via clariomshumantranscriptcluster.db |
| GSE11905 | Microarray (Operon v2 21k) | No raw data; outdated platform; limited annotation. | Not used in meta-analysis due to platform incompatibility and lack of raw files |

Abbreviations: CCCA, central centrifugal cicatricial alopecia; LPP, lichen planopilaris; FFA, frontal fibrosing alopecia; PPB, pseudopelade of Brocq; LS, lesional scalp; NL, non-lesional scalp; HC, healthy control; RNA-seq, RNA sequencing; FPKM, fragments per kilobase of exon model per million reads mapped. **For a subset of samples in GSE186075 (SRR16473800 to SRR16473781), only FPKM matrices were available in GEO. To enable count-based differential expression analysis, raw sequence files were retrieved from the SRA repository and converted to FASTQ using** fasterq-dump**. Quantification was performed using Salmon (quasi-mapping mode), and gene-level count matrices were generated for downstream analysis with DESeq2.**

****Supplementary** Table S10. Dataset-Specific Technical Limitations and Preprocessing Solutions**
This table summarises dataset-specific technical issues encountered during preprocessing and the strategies applied to ensure compatibility with downstream meta-analysis. FPKM-normalised files were excluded from differential expression analysis due to incompatibility with count-based statistical frameworks such as DESeq2 or edgeR. Where possible, raw count or .CEL files were prioritised. Reprocessing pipelines ensured standardised input across platforms.

| **GEO Accession** | **Diagnosis/Subtypes** | **Platform** | **Issue Identified** | **Resolution** | **Files Excluded** | **Justification** |
| --- | --- | --- | --- | --- | --- | --- |
| GSE186075 | **FFA**: GSM5631438–1441, 1445–1446 (n=6) **LPP**: GSM5631442–1444 (n=3) **HC**: GSM5631447–1458 (n=12) | Bulk RNA-seq (Illumina HiSeq 3000) | Only FPKM matrices available for subset | Retrieved corresponding SRA files, converted to FASTQ, quantified with Salmon to obtain gene-level counts | None | Ensured compatibility with count-based analysis by reprocessing SRR16473800–SRR16473781 |
|  | **LPP** (n=30), **FFA** (n=28), **CCCA** (n=11), **HC** (n=4) | Bulk RNA-seq (Illumina HiSeq 3000) | Raw counts provided as individual .counts.txt.gz files | Automated reading and merging into count matrix using R script | None | Raw count data compatible with DESeq2 pipeline |

**S3. Study-Specific Highlights and Contextual Summaries**

This section provides a concise summary of each included dataset, contextualizing their contributions to the literature, main findings, and methodological considerations. These narrative summaries complement the technical details reported in Tables and Suppementary Tables S8–S10 and enrich the interpretability of our meta-analysis.

**GSE186075**
Wang et al. conducted one of the most comprehensive transcriptomic studies on scarring alopecias, analysing bulk RNA-seq data from 30 LPP, 36 FFA, 9 CCCA patients, and 12 healthy controls. The study revealed a homogeneous gene expression profile across PCA subtypes, identifying a shared fibrotic signature and downregulation of cholesterogenic pathways. Notably, mast cell (MC)-associated genes were highly enriched and supported by immunohistochemistry, suggesting a dual role for MCs in inflammation and fibrosis. These findings reinforce the hypothesis of a convergent pathogenic process and the rationale for unified therapeutic strategies.
Cite as: [Wang et al., 2022, PNAS Nexus. PMID: 35899069]

(Wang EHC, Monga I, Sallee BN, Chen JC, Abdelaziz AR, Perez-Lorenzo R, Bordone LA, Christiano AM. Primary cicatricial alopecias are characterized by dysregulation of shared gene expression pathways. PNAS Nexus. 2022 Jul 11;1(3):pgac111. doi: 10.1093/pnasnexus/pgac111. PMID: 35899069; PMCID: PMC9308563.)

**GSE59131 and GSE58934**
These datasets were deposited without associated peer-reviewed publications. Both originated from the same research group, previously affiliated with Case Western Reserve University. Due to confirmed research misconduct in grant submissions by the lead author (as per ORI records), the reliability of study-level documentation is uncertain. However, raw CEL files were available and processed uniformly with other datasets.

**GSE179054**
Jamerson et al. profiled gene expression from lesional scalp biopsies of 16 CCCA patients stratified by disease severity. Severe forms exhibited upregulation of fibrotic genes (MMP9), Wnt signaling inhibitors (SFRP4), and macrophage markers (MSR1), suggesting biological divergence from focal or limited phenotypes. Although no external controls were included, the study provides compelling evidence for molecular heterogeneity in CCCA progression.
Cite as: [Jamerson et al., 2022, Exp Dermatol. PMID: 35007355]

(Jamerson TA, Conover Talbot C Jr, Dina Y, Kwatra SG, Garza LA, Aguh C. Gene expression profiling suggests severe, extensive central centrifugal cicatricial alopecia may be both clinically and biologically distinct from limited disease subtypes. Exp Dermatol. 2022 May;31(5):789-793. doi: 10.1111/exd.14524. Epub 2022 Jan 20. PMID: 35007355; PMCID: PMC9127746.)

**GSE113052**
In a paired design, lesional and non-lesional biopsies from 5 CCCA patients were analysed. Upregulated fibroproliferative genes (MMP2, COL1A1, PDGFA) and downregulation of lipid metabolism genes were observed. The intraindividual comparison minimized confounding from intersubject variability. Although limited by the absence of healthy controls and small sample size, the study highlighted pathways shared with systemic fibrotic diseases.
Cite as: [Aguh et al., 2018, J Am Acad Dermatol. PMID: 29913259]

(Aguh C, Dina Y, Talbot CC Jr, Garza L. Fibroproliferative genes are preferentially expressed in central centrifugal cicatricial alopecia. J Am Acad Dermatol. 2018 Nov;79(5):904-912.e1. doi: 10.1016/j.jaad.2018.05.1257. Epub 2018 Jun 18. PMID: 29913259; PMCID: PMC6348462.)

**GSE125733**
This dataset focused on FFA and was previously reanalysed by Liu et al. (2024), revealing roles for immune dysregulation and ferroptosis. While their analysis was restricted to a single dataset, our integrative meta-analysis placed GSE125733 in a broader context, revealing consistent immune activation and lipid pathway alterations across subtypes.
Cite as: [Liu et al., 2024, Skin Res Technol. PMID: 38314944] (Liu L, Xue Y, Wang J, Shen C, Li Y, Huang Y. Transcriptome analysis of frontal fibrosis alopecia revealed involvement of immune cells and ferroptosis. Skin Res Technol. 2024 Feb;30(2):e13608. doi: 10.1111/srt.13608. PMID: 38314944; PMCID: PMC10840369.)

**S4. Processing and Harmonization of Included Datasets**

All included transcriptomic datasets were harmonized to generate comparable gene-level expression matrices suitable for differential analysis. Due to platform heterogeneity (Affymetrix microarrays vs. Illumina bulk RNA-seq), dataset-specific preprocessing pipelines were applied as follows:

**RNA-seq Datasets**

Two RNA-seq datasets were included: GSE186075 and GSE125733. Although FPKM-normalized files were available for download from GEO, they were not used due to incompatibility with count-based statistical frameworks (e.g., DESeq2, edgeR). Instead, the raw SRA files were retrieved, converted to FASTQ format using fasterq-dump, and processed locally with Salmon v1.10.1 in quasi-mapping mode. Transcript-level estimates were aggregated to gene-level counts using the tximport package. This approach ensured the use of uniformly quantified, non-normalized count matrices across all RNA-seq datasets.

- In GSE186075, 21 samples (GSM5631438–GSM5631458) initially available only as FPKM files were recovered via this pipeline (see Table S9). The remaining RNA-seq samples (n = 73) were already available as raw count files in .counts.txt.gz format and were directly parsed into unified matrices, preserving gene annotations (Ensembl IDs or gene symbols).

**Affymetrix Microarray Datasets**

Five datasets were generated using Affymetrix microarrays:

- GSE59131 and GSE58934 (U133 Plus 2.0): Raw .CEL files were processed using the affy package with RMA (Robust Multiarray Average) normalization. Annotation was performed using the hgu133plus2.db Bioconductor package, mapping probes to official gene symbols.
- GSE179054 and GSE113052 (Clariom S): These were processed using the oligo package and RMA normalization. Due to differences in chip types and design, probesets were mapped to gene symbols using the clariomshumantranscriptcluster.db package.
- GSE11905 (Operon v2 21k platform) was only available as a processed matrix with no raw files and limited annotation metadata. Although the dataset was retained for exploratory analysis, it was excluded from the integrative meta-analysis due to its incompatibility with cross-platform normalization and its limited probe coverage.

**Cross-platform Harmonization**

To harmonize RNA-seq and microarray data for integrative analyses:

1. Gene symbols were used as the common identifier across all platforms.
2. For RNA-seq data, genes with consistently low counts across all samples were filtered out using a minimum count-per-million (CPM) threshold.
3. Microarray data were filtered to retain only probes with unambiguous gene symbol mappings and detectable expression in >20% of samples.
4. Normalized matrices from each dataset were merged by gene symbol using full outer joins, and batch effects were subsequently corrected using ComBat (from the sva package), accounting for study and platform as covariates.

All harmonized matrices, including raw counts, normalized expression values, and metadata files, are available upon request and described in detail in Supplementary Table S8. Dataset-specific technical issues and solutions are detailed in Supplementary Table S9.

**S5. Data Availability, Normalization Pipelines, and Analytical Plan by Dataset**

Each included dataset was assessed for data type availability, preprocessing compatibility, and suitability for integration into the meta-analysis. Table S10 summarizes the platform used, file formats available in GEO, and the specific normalization or preprocessing pipeline implemented.

**RNA-seq datasets (GSE186075, GSE125733):**

- GSE186075: Although FPKM matrices were deposited for a subset of samples (GSM5631438–GSM5631458), these were not used for downstream analyses due to their incompatibility with count-based differential methods. Instead, raw SRA files corresponding to these samples (SRR16473800–SRR16473781) were downloaded, converted to FASTQ using fasterq-dump, and quantified using Salmon in quasi-mapping mode. This yielded a raw gene-level count matrix annotated using Ensembl and Gene Symbols. These counts were merged with those provided directly as .counts.txt.gz files for other samples.
- GSE125733: Included both raw counts and FPKM data. Raw count matrices were used and genes were annotated from Entrez IDs to Gene Symbols prior to analysis using DESeq2.

**Microarray datasets:**

- Affymetrix U133 Plus 2.0 (GSE59131, GSE58934): Raw .CEL files were downloaded and normalized using the affy::rma() function. Gene annotations were mapped using the corresponding U133 Plus 2.0 annotation package. When both normalized matrix and CEL files were available, the raw files were prioritized to ensure uniform preprocessing.
- Affymetrix Clariom S (GSE179054, GSE113052): Datasets were processed from .CEL files using oligo::rma(). Probe-to-gene mapping was handled using the appropriate Clariom S annotation libraries. Each chip type was normalized separately before merging to ensure consistency.
- Operon v2 21k (GSE11905): Only a pre-normalized expression matrix was available. Given its platform incompatibility and absence of raw data, this dataset was retained for qualitative comparison but excluded from the core meta-analysis.

All datasets were aligned at the gene symbol level and expression values were log-transformed and batch-corrected using ComBat where applicable. Genes not detected across datasets were excluded from integrative analyses. The full analytical workflow applied to each dataset, including preprocessing scripts, is provided in Supplementary Table S10.

### ****S6. Differential Gene Expression and Functional Analysis****

**Differentially expressed genes (DEGs) were identified for each scarring alopecia subtype (FFA, LPP, CCCA) compared to controls, using batch-corrected expression matrices and linear modelling with limma and duplicateCorrelation. Genes with FDR-adjusted p-value < 0.05 and absolute log2 fold change > 1 were considered significant. The list of shared and subtype-specific DEGs is provided in Supplementary Table S11.**

Gene ontology (GO) and Reactome pathway enrichment analyses were performed separately for upregulated and downregulated genes using GeneCodis. Results are summarised visually in **Figures 3 and 4** of the main manuscript.

Candidate therapeutic compounds were identified through enrichment of drug-target gene signatures using LINCS and PharmGKB databases. Enrichment statistics and compound prioritisation results are presented in **Supplementary Table S12**.

**Table S11. Shared DEGs across subtypes and unique DEGs per subtype.**

This table shows the top differentially expressed genes (DEGs) for Frontal Fibrosing Alopecia (FFA), Lichen Planopilaris (LPP), and Central Centrifugal Cicatricial Alopecia (CCCA), identified using linear models with empirical Bayes moderation and duplicateCorrelation. Analysis was performed on ComBat-adjusted expression matrices including random effects for study and platform. Genes were selected based on criteria: unadjusted p-value < 0.05 and |log₂ fold change| > 0.5.

| **gene** | **logFC** | **AveExpr** | **t** | **adj.P.Val** | **B** | **condition** | **direction** | **regulation** | **N**  **significant** | **Comparisons**  **significant** | **Direction**  all |
| --- | --- | --- | --- | --- | --- | --- | --- | --- | --- | --- | --- |
| ARHGAP45 | 0.996 | 7.896 | 6.975 | 6.49e-08 | 14.018 | FFA_vs_Control | ↑ | up | 2 | FFA_vs_Control, LPP_vs_Control | FFA_vs_Control: ↑ \| LPP_vs_Control: ↑ |
| STMN2 | 1.236 | 9.063 | 6.466 | 3.74e-07 | 11.491 | FFA_vs_Control | ↑ | up | 2 | FFA_vs_Control, LPP_vs_Control | FFA_vs_Control: ↑ \| LPP_vs_Control: ↑ |
| TMEM140 | 0.608 | 7.31 | 6.441 | 3.87e-07 | 11.371 | FFA_vs_Control | ↑ | up | 3 | CCCA_vs_Control, FFA_vs_Control, LPP_vs_Control | FFA_vs_Control: ↑ \| LPP_vs_Control: ↑ \| CCCA_vs_Control: ↑ |
| CXCL10 | 1.871 | 6.084 | 6.437 | 3.87e-07 | 11.35 | FFA_vs_Control | ↑ | up | 1 | FFA_vs_Control | FFA_vs_Control: ↑ |
| NLRC3 | 0.903 | 6.794 | 6.383 | 4.6e-07 | 11.087 | FFA_vs_Control | ↑ | up | 1 | FFA_vs_Control | FFA_vs_Control: ↑ |
| CYTH4 | 0.869 | 7.417 | 6.328 | 5.08e-07 | 10.822 | FFA_vs_Control | ↑ | up | 1 | FFA_vs_Control | FFA_vs_Control: ↑ |
| MYO1F | 0.909 | 7.875 | 6.155 | 8.96e-07 | 9.999 | FFA_vs_Control | ↑ | up | 2 | FFA_vs_Control, LPP_vs_Control | FFA_vs_Control: ↑ \| LPP_vs_Control: ↑ |
| DENND1C | 0.862 | 7.609 | 6.091 | 1.07e-06 | 9.694 | FFA_vs_Control | ↑ | up | 2 | FFA_vs_Control, LPP_vs_Control | FFA_vs_Control: ↑ \| LPP_vs_Control: ↑ |
| CD40 | 0.53 | 7.684 | 6.078 | 1.13e-06 | 9.634 | FFA_vs_Control | ↑ | up | 1 | FFA_vs_Control | FFA_vs_Control: ↑ |
| TBC1D10C | 0.927 | 7.185 | 6.069 | 1.15e-06 | 9.592 | FFA_vs_Control | ↑ | up | 1 | FFA_vs_Control | FFA_vs_Control: ↑ |
| IRF1 | 0.881 | 8.274 | 6.043 | 1.23e-06 | 9.468 | FFA_vs_Control | ↑ | up | 1 | FFA_vs_Control | FFA_vs_Control: ↑ |
| GBP5 | 1.196 | 6.497 | 6.033 | 1.28e-06 | 9.422 | FFA_vs_Control | ↑ | up | 1 | FFA_vs_Control | FFA_vs_Control: ↑ |
| GZMB | 0.744 | 5.943 | 6.003 | 1.44e-06 | 9.282 | FFA_vs_Control | ↑ | up | 1 | FFA_vs_Control | FFA_vs_Control: ↑ |
| ARHGAP4 | 0.856 | 8.093 | 5.984 | 1.55e-06 | 9.194 | FFA_vs_Control | ↑ | up | 1 | FFA_vs_Control | FFA_vs_Control: ↑ |
| BIRC3 | 0.919 | 8.005 | 5.981 | 1.56e-06 | 9.178 | FFA_vs_Control | ↑ | up | 1 | FFA_vs_Control | FFA_vs_Control: ↑ |
| TNFAIP2 | 0.752 | 9.402 | 5.973 | 1.59e-06 | 9.143 | FFA_vs_Control | ↑ | up | 1 | FFA_vs_Control | FFA_vs_Control: ↑ |
| IFFO1 | 0.622 | 7.938 | 5.927 | 1.88e-06 | 8.927 | FFA_vs_Control | ↑ | up | 1 | FFA_vs_Control | FFA_vs_Control: ↑ |
| SIGLEC1 | 0.86 | 7.739 | 5.916 | 1.94e-06 | 8.88 | FFA_vs_Control | ↑ | up | 1 | FFA_vs_Control | FFA_vs_Control: ↑ |
| HK3 | 0.69 | 6.107 | 5.904 | 2.03e-06 | 8.825 | FFA_vs_Control | ↑ | up | 1 | FFA_vs_Control | FFA_vs_Control: ↑ |
| SNX22 | 0.776 | 7.0 | 5.848 | 2.45e-06 | 8.563 | FFA_vs_Control | ↑ | up | 3 | CCCA_vs_Control, FFA_vs_Control, LPP_vs_Control | FFA_vs_Control: ↑ \| LPP_vs_Control: ↑ \| CCCA_vs_Control: ↑ |
| BOD1L1 | 0.518 | 9.264 | 5.82 | 2.71e-06 | 8.434 | FFA_vs_Control | ↑ | up | 1 | FFA_vs_Control | FFA_vs_Control: ↑ |
| CXCL9 | 1.999 | 6.87 | 5.812 | 2.76e-06 | 8.397 | FFA_vs_Control | ↑ | up | 1 | FFA_vs_Control | FFA_vs_Control: ↑ |
| APOL3 | 0.744 | 8.138 | 5.809 | 2.78e-06 | 8.384 | FFA_vs_Control | ↑ | up | 1 | FFA_vs_Control | FFA_vs_Control: ↑ |
| STAB1 | 0.872 | 8.731 | 5.767 | 3.24e-06 | 8.191 | FFA_vs_Control | ↑ | up | 2 | FFA_vs_Control, LPP_vs_Control | FFA_vs_Control: ↑ \| LPP_vs_Control: ↑ |
| BATF | 0.584 | 6.587 | 5.75 | 3.41e-06 | 8.115 | FFA_vs_Control | ↑ | up | 1 | FFA_vs_Control | FFA_vs_Control: ↑ |
| RGL4 | 0.642 | 5.923 | 5.744 | 3.46e-06 | 8.089 | FFA_vs_Control | ↑ | up | 1 | FFA_vs_Control | FFA_vs_Control: ↑ |
| CD8A | 0.896 | 6.435 | 5.699 | 4.09e-06 | 7.883 | FFA_vs_Control | ↑ | up | 1 | FFA_vs_Control | FFA_vs_Control: ↑ |
| GPR132 | 0.654 | 6.231 | 5.682 | 4.29e-06 | 7.81 | FFA_vs_Control | ↑ | up | 1 | FFA_vs_Control | FFA_vs_Control: ↑ |
| PRF1 | 0.634 | 6.441 | 5.682 | 4.29e-06 | 7.807 | FFA_vs_Control | ↑ | up | 1 | FFA_vs_Control | FFA_vs_Control: ↑ |
| SLC51A | 0.75 | 6.282 | 5.642 | 4.9e-06 | 7.627 | FFA_vs_Control | ↑ | up | 3 | CCCA_vs_Control, FFA_vs_Control, LPP_vs_Control | FFA_vs_Control: ↑ \| LPP_vs_Control: ↑ \| CCCA_vs_Control: ↑ |
| HELB | 0.873 | 6.491 | 5.606 | 5.41e-06 | 7.464 | FFA_vs_Control | ↑ | up | 2 | FFA_vs_Control, LPP_vs_Control | FFA_vs_Control: ↑ \| LPP_vs_Control: ↑ |
| ARHGAP9 | 0.778 | 7.376 | 5.602 | 5.47e-06 | 7.447 | FFA_vs_Control | ↑ | up | 1 | FFA_vs_Control | FFA_vs_Control: ↑ |
| BST2 | 0.845 | 7.292 | 5.587 | 5.67e-06 | 7.382 | FFA_vs_Control | ↑ | up | 1 | FFA_vs_Control | FFA_vs_Control: ↑ |
| TMEM266 | 0.639 | 6.538 | 5.562 | 6.25e-06 | 7.269 | FFA_vs_Control | ↑ | up | 2 | FFA_vs_Control, LPP_vs_Control | FFA_vs_Control: ↑ \| LPP_vs_Control: ↑ |
| APOL1 | 0.723 | 7.666 | 5.521 | 7.19e-06 | 7.087 | FFA_vs_Control | ↑ | up | 1 | FFA_vs_Control | FFA_vs_Control: ↑ |
| RAB37 | 0.586 | 6.046 | 5.509 | 7.38e-06 | 7.033 | FFA_vs_Control | ↑ | up | 1 | FFA_vs_Control | FFA_vs_Control: ↑ |
| ZAP70 | 0.937 | 6.955 | 5.499 | 7.68e-06 | 6.987 | FFA_vs_Control | ↑ | up | 1 | FFA_vs_Control | FFA_vs_Control: ↑ |
| SAMD9L | 0.875 | 7.875 | 5.459 | 8.69e-06 | 6.81 | FFA_vs_Control | ↑ | up | 1 | FFA_vs_Control | FFA_vs_Control: ↑ |
| ETV7 | 0.564 | 6.99 | 5.455 | 8.78e-06 | 6.793 | FFA_vs_Control | ↑ | up | 1 | FFA_vs_Control | FFA_vs_Control: ↑ |
| CCR7 | 0.834 | 6.189 | 5.429 | 9.56e-06 | 6.681 | FFA_vs_Control | ↑ | up | 1 | FFA_vs_Control | FFA_vs_Control: ↑ |
| NLRC5 | 0.818 | 9.16 | 5.419 | 9.78e-06 | 6.638 | FFA_vs_Control | ↑ | up | 1 | FFA_vs_Control | FFA_vs_Control: ↑ |
| FERMT3 | 0.649 | 7.329 | 5.397 | 1.06e-05 | 6.539 | FFA_vs_Control | ↑ | up | 1 | FFA_vs_Control | FFA_vs_Control: ↑ |
| MYO1G | 0.745 | 7.113 | 5.375 | 1.15e-05 | 6.445 | FFA_vs_Control | ↑ | up | 1 | FFA_vs_Control | FFA_vs_Control: ↑ |
| VAV1 | 0.582 | 7.017 | 5.351 | 1.26e-05 | 6.341 | FFA_vs_Control | ↑ | up | 1 | FFA_vs_Control | FFA_vs_Control: ↑ |
| LTB | 0.769 | 7.265 | 5.337 | 1.33e-05 | 6.279 | FFA_vs_Control | ↑ | up | 1 | FFA_vs_Control | FFA_vs_Control: ↑ |
| DOCK10 | 0.706 | 7.423 | 5.322 | 1.4e-05 | 6.213 | FFA_vs_Control | ↑ | up | 1 | FFA_vs_Control | FFA_vs_Control: ↑ |
| S1PR4 | 0.539 | 6.1 | 5.311 | 1.44e-05 | 6.166 | FFA_vs_Control | ↑ | up | 1 | FFA_vs_Control | FFA_vs_Control: ↑ |
| PSMB9 | 0.865 | 8.334 | 5.309 | 1.45e-05 | 6.159 | FFA_vs_Control | ↑ | up | 1 | FFA_vs_Control | FFA_vs_Control: ↑ |
| LAX1 | 0.667 | 6.319 | 5.287 | 1.56e-05 | 6.062 | FFA_vs_Control | ↑ | up | 1 | FFA_vs_Control | FFA_vs_Control: ↑ |
| LIPE | 1.137 | 8.62 | 5.285 | 1.57e-05 | 6.056 | FFA_vs_Control | ↑ | up | 1 | FFA_vs_Control | FFA_vs_Control: ↑ |
| FOXC1 | -1.132 | 9.601 | -8.095 | 3.38e-09 | 19.875 | FFA_vs_Control | ↓ | down | 2 | FFA_vs_Control, LPP_vs_Control | FFA_vs_Control: ↓ \| LPP_vs_Control: ↓ |
| SLC25A23 | -0.621 | 8.79 | -7.899 | 3.49e-09 | 18.821 | FFA_vs_Control | ↓ | down | 2 | FFA_vs_Control, LPP_vs_Control | FFA_vs_Control: ↓ \| LPP_vs_Control: ↓ |
| FAXDC2 | -1.052 | 10.202 | -7.894 | 3.49e-09 | 18.795 | FFA_vs_Control | ↓ | down | 3 | CCCA_vs_Control, FFA_vs_Control, LPP_vs_Control | FFA_vs_Control: ↓ \| LPP_vs_Control: ↓ \| CCCA_vs_Control: ↓ |
| ZBED3 | -0.999 | 7.475 | -7.633 | 1.11e-08 | 17.414 | FFA_vs_Control | ↓ | down | 2 | FFA_vs_Control, LPP_vs_Control | FFA_vs_Control: ↓ \| LPP_vs_Control: ↓ |
| EIF1AD | -0.581 | 8.461 | -7.534 | 1.53e-08 | 16.896 | FFA_vs_Control | ↓ | down | 3 | CCCA_vs_Control, FFA_vs_Control, LPP_vs_Control | FFA_vs_Control: ↓ \| LPP_vs_Control: ↓ \| CCCA_vs_Control: ↓ |
| RGP1 | -0.572 | 9.046 | -7.5 | 1.53e-08 | 16.718 | FFA_vs_Control | ↓ | down | 2 | FFA_vs_Control, LPP_vs_Control | FFA_vs_Control: ↓ \| LPP_vs_Control: ↓ |
| ACOX2 | -1.076 | 7.862 | -7.38 | 2.53e-08 | 16.094 | FFA_vs_Control | ↓ | down | 3 | CCCA_vs_Control, FFA_vs_Control, LPP_vs_Control | FFA_vs_Control: ↓ \| LPP_vs_Control: ↓ \| CCCA_vs_Control: ↓ |
| TGFB2 | -1.034 | 7.317 | -7.327 | 2.95e-08 | 15.82 | FFA_vs_Control | ↓ | down | 1 | FFA_vs_Control | FFA_vs_Control: ↓ |
| SLC26A3 | -1.012 | 5.062 | -7.293 | 3.16e-08 | 15.641 | FFA_vs_Control | ↓ | down | 1 | FFA_vs_Control | FFA_vs_Control: ↓ |
| SNAP29 | -0.638 | 8.837 | -7.262 | 3.19e-08 | 15.481 | FFA_vs_Control | ↓ | down | 2 | FFA_vs_Control, LPP_vs_Control | FFA_vs_Control: ↓ \| LPP_vs_Control: ↓ |
| CERS4 | -0.925 | 9.425 | -7.23 | 3.19e-08 | 15.318 | FFA_vs_Control | ↓ | down | 1 | FFA_vs_Control | FFA_vs_Control: ↓ |
| CUX2 | -1.375 | 5.861 | -7.226 | 3.19e-08 | 15.299 | FFA_vs_Control | ↓ | down | 1 | FFA_vs_Control | FFA_vs_Control: ↓ |
| TP53INP2 | -0.977 | 9.025 | -7.223 | 3.19e-08 | 15.28 | FFA_vs_Control | ↓ | down | 1 | FFA_vs_Control | FFA_vs_Control: ↓ |
| SLC9A1 | -0.512 | 8.615 | -7.197 | 3.39e-08 | 15.151 | FFA_vs_Control | ↓ | down | 1 | FFA_vs_Control | FFA_vs_Control: ↓ |
| CPT2 | -0.795 | 8.061 | -7.122 | 4.41e-08 | 14.763 | FFA_vs_Control | ↓ | down | 3 | CCCA_vs_Control, FFA_vs_Control, LPP_vs_Control | FFA_vs_Control: ↓ \| LPP_vs_Control: ↓ \| CCCA_vs_Control: ↓ |
| DAG1 | -0.722 | 9.639 | -7.111 | 4.41e-08 | 14.71 | FFA_vs_Control | ↓ | down | 1 | FFA_vs_Control | FFA_vs_Control: ↓ |
| HIPK2 | -0.631 | 9.483 | -7.104 | 4.41e-08 | 14.672 | FFA_vs_Control | ↓ | down | 1 | FFA_vs_Control | FFA_vs_Control: ↓ |
| ISM1 | -0.8 | 8.273 | -7.098 | 4.41e-08 | 14.641 | FFA_vs_Control | ↓ | down | 3 | CCCA_vs_Control, FFA_vs_Control, LPP_vs_Control | FFA_vs_Control: ↓ \| LPP_vs_Control: ↓ \| CCCA_vs_Control: ↓ |
| ADGRG2 | -0.761 | 5.965 | -7.091 | 4.41e-08 | 14.609 | FFA_vs_Control | ↓ | down | 3 | CCCA_vs_Control, FFA_vs_Control, LPP_vs_Control | FFA_vs_Control: ↓ \| LPP_vs_Control: ↓ \| CCCA_vs_Control: ↓ |
| ZDHHC9 | -0.898 | 9.192 | -7.026 | 5.94e-08 | 14.276 | FFA_vs_Control | ↓ | down | 3 | CCCA_vs_Control, FFA_vs_Control, LPP_vs_Control | FFA_vs_Control: ↓ \| LPP_vs_Control: ↓ \| CCCA_vs_Control: ↓ |
| SC5D | -1.43 | 9.133 | -6.993 | 6.49e-08 | 14.11 | FFA_vs_Control | ↓ | down | 3 | CCCA_vs_Control, FFA_vs_Control, LPP_vs_Control | FFA_vs_Control: ↓ \| LPP_vs_Control: ↓ \| CCCA_vs_Control: ↓ |
| TECR | -0.69 | 10.151 | -6.984 | 6.49e-08 | 14.063 | FFA_vs_Control | ↓ | down | 1 | FFA_vs_Control | FFA_vs_Control: ↓ |
| CCDC86 | -0.711 | 8.282 | -6.98 | 6.49e-08 | 14.041 | FFA_vs_Control | ↓ | down | 2 | FFA_vs_Control, LPP_vs_Control | FFA_vs_Control: ↓ \| LPP_vs_Control: ↓ |
| ATP6V0C | -1.481 | 9.626 | -6.966 | 6.52e-08 | 13.975 | FFA_vs_Control | ↓ | down | 2 | FFA_vs_Control, LPP_vs_Control | FFA_vs_Control: ↓ \| LPP_vs_Control: ↓ |
| SLC25A35 | -1.079 | 7.443 | -6.934 | 7.35e-08 | 13.81 | FFA_vs_Control | ↓ | down | 1 | FFA_vs_Control | FFA_vs_Control: ↓ |
| PAFAH2 | -0.692 | 8.128 | -6.926 | 7.35e-08 | 13.772 | FFA_vs_Control | ↓ | down | 3 | CCCA_vs_Control, FFA_vs_Control, LPP_vs_Control | FFA_vs_Control: ↓ \| LPP_vs_Control: ↓ \| CCCA_vs_Control: ↓ |
| AGPAT3 | -1.14 | 9.84 | -6.922 | 7.35e-08 | 13.752 | FFA_vs_Control | ↓ | down | 3 | CCCA_vs_Control, FFA_vs_Control, LPP_vs_Control | FFA_vs_Control: ↓ \| LPP_vs_Control: ↓ \| CCCA_vs_Control: ↓ |
| PRKY | -2.765 | 5.665 | -6.885 | 8.63e-08 | 13.565 | FFA_vs_Control | ↓ | down | 1 | FFA_vs_Control | FFA_vs_Control: ↓ |
| FAM234A | -0.582 | 9.271 | -6.872 | 8.92e-08 | 13.501 | FFA_vs_Control | ↓ | down | 1 | FFA_vs_Control | FFA_vs_Control: ↓ |
| KNSTRN | -1.032 | 7.962 | -6.838 | 1.04e-07 | 13.327 | FFA_vs_Control | ↓ | down | 2 | CCCA_vs_Control, FFA_vs_Control | FFA_vs_Control: ↓ \| CCCA_vs_Control: ↓ |
| INSIG1 | -1.652 | 10.185 | -6.825 | 1.07e-07 | 13.264 | FFA_vs_Control | ↓ | down | 1 | FFA_vs_Control | FFA_vs_Control: ↓ |
| MCCC2 | -0.795 | 8.91 | -6.769 | 1.36e-07 | 12.986 | FFA_vs_Control | ↓ | down | 3 | CCCA_vs_Control, FFA_vs_Control, LPP_vs_Control | FFA_vs_Control: ↓ \| LPP_vs_Control: ↓ \| CCCA_vs_Control: ↓ |
| GATA6 | -0.952 | 7.058 | -6.766 | 1.36e-07 | 12.97 | FFA_vs_Control | ↓ | down | 2 | CCCA_vs_Control, FFA_vs_Control | FFA_vs_Control: ↓ \| CCCA_vs_Control: ↓ |
| ACSS2 | -1.054 | 9.816 | -6.763 | 1.36e-07 | 12.954 | FFA_vs_Control | ↓ | down | 3 | CCCA_vs_Control, FFA_vs_Control, LPP_vs_Control | FFA_vs_Control: ↓ \| LPP_vs_Control: ↓ \| CCCA_vs_Control: ↓ |
| ACY1 | -1.278 | 7.079 | -6.756 | 1.37e-07 | 12.919 | FFA_vs_Control | ↓ | down | 2 | FFA_vs_Control, LPP_vs_Control | FFA_vs_Control: ↓ \| LPP_vs_Control: ↓ |
| TMEM164 | -1.186 | 9.811 | -6.739 | 1.46e-07 | 12.833 | FFA_vs_Control | ↓ | down | 2 | CCCA_vs_Control, FFA_vs_Control | FFA_vs_Control: ↓ \| CCCA_vs_Control: ↓ |
| GNAL | -0.674 | 8.608 | -6.723 | 1.54e-07 | 12.757 | FFA_vs_Control | ↓ | down | 1 | FFA_vs_Control | FFA_vs_Control: ↓ |
| SLC27A2 | -1.343 | 6.176 | -6.708 | 1.62e-07 | 12.68 | FFA_vs_Control | ↓ | down | 3 | CCCA_vs_Control, FFA_vs_Control, LPP_vs_Control | FFA_vs_Control: ↓ \| LPP_vs_Control: ↓ \| CCCA_vs_Control: ↓ |
| BEAN1 | -1.142 | 7.538 | -6.699 | 1.65e-07 | 12.638 | FFA_vs_Control | ↓ | down | 2 | CCCA_vs_Control, FFA_vs_Control | FFA_vs_Control: ↓ \| CCCA_vs_Control: ↓ |
| PDZK1 | -2.018 | 6.986 | -6.694 | 1.65e-07 | 12.613 | FFA_vs_Control | ↓ | down | 2 | CCCA_vs_Control, FFA_vs_Control | FFA_vs_Control: ↓ \| CCCA_vs_Control: ↓ |
| KLK1 | -0.711 | 7.88 | -6.69 | 1.65e-07 | 12.591 | FFA_vs_Control | ↓ | down | 1 | FFA_vs_Control | FFA_vs_Control: ↓ |
| AGPAT1 | -1.028 | 9.228 | -6.668 | 1.77e-07 | 12.482 | FFA_vs_Control | ↓ | down | 3 | CCCA_vs_Control, FFA_vs_Control, LPP_vs_Control | FFA_vs_Control: ↓ \| LPP_vs_Control: ↓ \| CCCA_vs_Control: ↓ |
| MUC1 | -1.859 | 7.801 | -6.668 | 1.77e-07 | 12.482 | FFA_vs_Control | ↓ | down | 2 | CCCA_vs_Control, FFA_vs_Control | FFA_vs_Control: ↓ \| CCCA_vs_Control: ↓ |
| WASL | -0.676 | 9.304 | -6.643 | 1.97e-07 | 12.358 | FFA_vs_Control | ↓ | down | 2 | FFA_vs_Control, LPP_vs_Control | FFA_vs_Control: ↓ \| LPP_vs_Control: ↓ |
| BCKDHB | -0.809 | 8.176 | -6.606 | 2.33e-07 | 12.179 | FFA_vs_Control | ↓ | down | 3 | CCCA_vs_Control, FFA_vs_Control, LPP_vs_Control | FFA_vs_Control: ↓ \| LPP_vs_Control: ↓ \| CCCA_vs_Control: ↓ |
| FAM222B | -0.538 | 7.895 | -6.588 | 2.51e-07 | 12.087 | FFA_vs_Control | ↓ | down | 1 | FFA_vs_Control | FFA_vs_Control: ↓ |
| PXMP2 | -0.711 | 8.231 | -6.568 | 2.66e-07 | 11.99 | FFA_vs_Control | ↓ | down | 1 | FFA_vs_Control | FFA_vs_Control: ↓ |
| CTNND2 | -1.097 | 7.374 | -6.555 | 2.79e-07 | 11.928 | FFA_vs_Control | ↓ | down | 1 | FFA_vs_Control | FFA_vs_Control: ↓ |
| THRSP | -1.84 | 10.204 | -6.551 | 2.79e-07 | 11.908 | FFA_vs_Control | ↓ | down | 1 | FFA_vs_Control | FFA_vs_Control: ↓ |
| LPCAT3 | -1.47 | 8.675 | -6.546 | 2.81e-07 | 11.885 | FFA_vs_Control | ↓ | down | 1 | FFA_vs_Control | FFA_vs_Control: ↓ |
| PLA2G2A | 1.431 | 8.882 | 6.386 | 5.98e-07 | 11.104 | LPP_vs_Control | ↑ | up | 1 | LPP_vs_Control | LPP_vs_Control: ↑ |
| MXRA8 | 0.786 | 9.599 | 6.29 | 7.8e-07 | 10.64 | LPP_vs_Control | ↑ | up | 2 | CCCA_vs_Control, LPP_vs_Control | LPP_vs_Control: ↑ \| CCCA_vs_Control: ↑ |
| STMN2 | 1.2 | 9.063 | 6.179 | 1.12e-06 | 10.112 | LPP_vs_Control | ↑ | up | 2 | FFA_vs_Control, LPP_vs_Control | FFA_vs_Control: ↑ \| LPP_vs_Control: ↑ |
| EVA1B | 1.159 | 7.808 | 6.122 | 1.22e-06 | 9.84 | LPP_vs_Control | ↑ | up | 2 | CCCA_vs_Control, LPP_vs_Control | LPP_vs_Control: ↑ \| CCCA_vs_Control: ↑ |
| CPXM1 | 0.881 | 7.895 | 6.11 | 1.23e-06 | 9.784 | LPP_vs_Control | ↑ | up | 1 | LPP_vs_Control | LPP_vs_Control: ↑ |
| EMILIN1 | 0.95 | 8.089 | 5.996 | 1.71e-06 | 9.253 | LPP_vs_Control | ↑ | up | 2 | CCCA_vs_Control, LPP_vs_Control | LPP_vs_Control: ↑ \| CCCA_vs_Control: ↑ |
| TMEM140 | 0.558 | 7.31 | 5.817 | 2.97e-06 | 8.427 | LPP_vs_Control | ↑ | up | 3 | CCCA_vs_Control, FFA_vs_Control, LPP_vs_Control | FFA_vs_Control: ↑ \| LPP_vs_Control: ↑ \| CCCA_vs_Control: ↑ |
| SLC51A | 0.776 | 6.282 | 5.749 | 3.64e-06 | 8.113 | LPP_vs_Control | ↑ | up | 3 | CCCA_vs_Control, FFA_vs_Control, LPP_vs_Control | FFA_vs_Control: ↑ \| LPP_vs_Control: ↑ \| CCCA_vs_Control: ↑ |
| SNX22 | 0.768 | 7.0 | 5.699 | 4.21e-06 | 7.886 | LPP_vs_Control | ↑ | up | 3 | CCCA_vs_Control, FFA_vs_Control, LPP_vs_Control | FFA_vs_Control: ↑ \| LPP_vs_Control: ↑ \| CCCA_vs_Control: ↑ |
| ANKRD36C | 0.882 | 7.315 | 5.666 | 4.67e-06 | 7.737 | LPP_vs_Control | ↑ | up | 1 | LPP_vs_Control | LPP_vs_Control: ↑ |
| C22orf23 | 1.104 | 6.682 | 5.647 | 4.95e-06 | 7.655 | LPP_vs_Control | ↑ | up | 2 | CCCA_vs_Control, LPP_vs_Control | LPP_vs_Control: ↑ \| CCCA_vs_Control: ↑ |
| ZBTB20 | 1.264 | 8.829 | 5.587 | 5.84e-06 | 7.387 | LPP_vs_Control | ↑ | up | 2 | CCCA_vs_Control, LPP_vs_Control | LPP_vs_Control: ↑ \| CCCA_vs_Control: ↑ |
| ZC3HAV1L | 0.513 | 6.977 | 5.544 | 6.7e-06 | 7.193 | LPP_vs_Control | ↑ | up | 1 | LPP_vs_Control | LPP_vs_Control: ↑ |
| RCN3 | 0.711 | 8.294 | 5.53 | 7.03e-06 | 7.131 | LPP_vs_Control | ↑ | up | 1 | LPP_vs_Control | LPP_vs_Control: ↑ |
| APC2 | 0.662 | 6.612 | 5.355 | 1.19e-05 | 6.363 | LPP_vs_Control | ↑ | up | 2 | CCCA_vs_Control, LPP_vs_Control | LPP_vs_Control: ↑ \| CCCA_vs_Control: ↑ |
| PERM1 | 1.487 | 7.064 | 5.341 | 1.24e-05 | 6.303 | LPP_vs_Control | ↑ | up | 2 | CCCA_vs_Control, LPP_vs_Control | LPP_vs_Control: ↑ \| CCCA_vs_Control: ↑ |
| G0S2 | 1.328 | 10.028 | 5.315 | 1.35e-05 | 6.188 | LPP_vs_Control | ↑ | up | 2 | CCCA_vs_Control, LPP_vs_Control | LPP_vs_Control: ↑ \| CCCA_vs_Control: ↑ |
| HELB | 0.841 | 6.491 | 5.312 | 1.36e-05 | 6.179 | LPP_vs_Control | ↑ | up | 2 | FFA_vs_Control, LPP_vs_Control | FFA_vs_Control: ↑ \| LPP_vs_Control: ↑ |
| ASB16 | 0.806 | 6.545 | 5.304 | 1.38e-05 | 6.142 | LPP_vs_Control | ↑ | up | 1 | LPP_vs_Control | LPP_vs_Control: ↑ |
| SPRN | 0.789 | 6.532 | 5.293 | 1.43e-05 | 6.094 | LPP_vs_Control | ↑ | up | 1 | LPP_vs_Control | LPP_vs_Control: ↑ |
| KRT13 | 0.908 | 6.206 | 5.284 | 1.46e-05 | 6.056 | LPP_vs_Control | ↑ | up | 1 | LPP_vs_Control | LPP_vs_Control: ↑ |
| TMEM266 | 0.617 | 6.538 | 5.278 | 1.48e-05 | 6.029 | LPP_vs_Control | ↑ | up | 2 | FFA_vs_Control, LPP_vs_Control | FFA_vs_Control: ↑ \| LPP_vs_Control: ↑ |
| IGFL1 | 0.973 | 6.296 | 5.249 | 1.61e-05 | 5.904 | LPP_vs_Control | ↑ | up | 2 | CCCA_vs_Control, LPP_vs_Control | LPP_vs_Control: ↑ \| CCCA_vs_Control: ↑ |
| DENND1C | 0.744 | 7.609 | 5.178 | 2.03e-05 | 5.602 | LPP_vs_Control | ↑ | up | 2 | FFA_vs_Control, LPP_vs_Control | FFA_vs_Control: ↑ \| LPP_vs_Control: ↑ |
| STAB1 | 0.792 | 8.731 | 5.158 | 2.17e-05 | 5.516 | LPP_vs_Control | ↑ | up | 2 | FFA_vs_Control, LPP_vs_Control | FFA_vs_Control: ↑ \| LPP_vs_Control: ↑ |
| BRSK2 | 0.665 | 5.933 | 5.151 | 2.21e-05 | 5.487 | LPP_vs_Control | ↑ | up | 1 | LPP_vs_Control | LPP_vs_Control: ↑ |
| ZFHX4 | 0.608 | 6.872 | 5.103 | 2.62e-05 | 5.286 | LPP_vs_Control | ↑ | up | 1 | LPP_vs_Control | LPP_vs_Control: ↑ |
| CRMP1 | 0.935 | 7.258 | 5.094 | 2.72e-05 | 5.246 | LPP_vs_Control | ↑ | up | 1 | LPP_vs_Control | LPP_vs_Control: ↑ |
| NOS3 | 0.676 | 7.303 | 5.064 | 2.99e-05 | 5.122 | LPP_vs_Control | ↑ | up | 1 | LPP_vs_Control | LPP_vs_Control: ↑ |
| RARRES2 | 0.84 | 9.628 | 5.052 | 3.08e-05 | 5.072 | LPP_vs_Control | ↑ | up | 1 | LPP_vs_Control | LPP_vs_Control: ↑ |
| ARHGAP45 | 0.732 | 7.896 | 5.045 | 3.14e-05 | 5.04 | LPP_vs_Control | ↑ | up | 2 | FFA_vs_Control, LPP_vs_Control | FFA_vs_Control: ↑ \| LPP_vs_Control: ↑ |
| NRIP2 | 0.53 | 6.748 | 5.039 | 3.21e-05 | 5.019 | LPP_vs_Control | ↑ | up | 2 | CCCA_vs_Control, LPP_vs_Control | LPP_vs_Control: ↑ \| CCCA_vs_Control: ↑ |
| ZIC1 | 0.622 | 7.761 | 4.996 | 3.7e-05 | 4.838 | LPP_vs_Control | ↑ | up | 1 | LPP_vs_Control | LPP_vs_Control: ↑ |
| PKDCC | 0.588 | 7.858 | 4.968 | 4.02e-05 | 4.724 | LPP_vs_Control | ↑ | up | 2 | CCCA_vs_Control, LPP_vs_Control | LPP_vs_Control: ↑ \| CCCA_vs_Control: ↑ |
| PTH1R | 0.569 | 7.174 | 4.954 | 4.22e-05 | 4.664 | LPP_vs_Control | ↑ | up | 1 | LPP_vs_Control | LPP_vs_Control: ↑ |
| MYO1F | 0.737 | 7.875 | 4.91 | 4.92e-05 | 4.482 | LPP_vs_Control | ↑ | up | 2 | FFA_vs_Control, LPP_vs_Control | FFA_vs_Control: ↑ \| LPP_vs_Control: ↑ |
| HBB | 2.052 | 11.52 | 4.885 | 5.29e-05 | 4.382 | LPP_vs_Control | ↑ | up | 1 | LPP_vs_Control | LPP_vs_Control: ↑ |
| PTGDS | 1.033 | 10.167 | 4.866 | 5.59e-05 | 4.306 | LPP_vs_Control | ↑ | up | 1 | LPP_vs_Control | LPP_vs_Control: ↑ |
| SLC16A8 | 0.691 | 6.237 | 4.814 | 6.57e-05 | 4.093 | LPP_vs_Control | ↑ | up | 1 | LPP_vs_Control | LPP_vs_Control: ↑ |
| FSTL3 | 0.545 | 8.331 | 4.747 | 8.19e-05 | 3.825 | LPP_vs_Control | ↑ | up | 1 | LPP_vs_Control | LPP_vs_Control: ↑ |
| MASP2 | 0.789 | 6.185 | 4.744 | 8.25e-05 | 3.814 | LPP_vs_Control | ↑ | up | 1 | LPP_vs_Control | LPP_vs_Control: ↑ |
| ARPC1B | 0.658 | 9.328 | 4.741 | 8.33e-05 | 3.801 | LPP_vs_Control | ↑ | up | 1 | LPP_vs_Control | LPP_vs_Control: ↑ |
| EXOC3L1 | 0.532 | 6.877 | 4.714 | 9.03e-05 | 3.693 | LPP_vs_Control | ↑ | up | 2 | CCCA_vs_Control, LPP_vs_Control | LPP_vs_Control: ↑ \| CCCA_vs_Control: ↑ |
| ADAMTS12 | 0.603 | 7.417 | 4.697 | 9.57e-05 | 3.625 | LPP_vs_Control | ↑ | up | 1 | LPP_vs_Control | LPP_vs_Control: ↑ |
| PTX3 | 0.663 | 5.516 | 4.684 | 0.0001 | 3.572 | LPP_vs_Control | ↑ | up | 1 | LPP_vs_Control | LPP_vs_Control: ↑ |
| OPN1SW | 0.543 | 5.251 | 4.68 | 0.000101 | 3.559 | LPP_vs_Control | ↑ | up | 1 | LPP_vs_Control | LPP_vs_Control: ↑ |
| PKN1 | 0.536 | 8.505 | 4.666 | 0.000106 | 3.502 | LPP_vs_Control | ↑ | up | 2 | CCCA_vs_Control, LPP_vs_Control | LPP_vs_Control: ↑ \| CCCA_vs_Control: ↑ |
| ANKRD12 | 0.72 | 9.995 | 4.666 | 0.000106 | 3.501 | LPP_vs_Control | ↑ | up | 1 | LPP_vs_Control | LPP_vs_Control: ↑ |
| INHBA | 0.574 | 7.689 | 4.661 | 0.000108 | 3.484 | LPP_vs_Control | ↑ | up | 2 | CCCA_vs_Control, LPP_vs_Control | LPP_vs_Control: ↑ \| CCCA_vs_Control: ↑ |
| IL3RA | 0.608 | 7.164 | 4.654 | 0.00011 | 3.455 | LPP_vs_Control | ↑ | up | 2 | CCCA_vs_Control, LPP_vs_Control | LPP_vs_Control: ↑ \| CCCA_vs_Control: ↑ |
| SLC25A23 | -0.635 | 8.79 | -7.95 | 7.66e-09 | 19.079 | LPP_vs_Control | ↓ | down | 2 | FFA_vs_Control, LPP_vs_Control | FFA_vs_Control: ↓ \| LPP_vs_Control: ↓ |
| FOXC1 | -1.104 | 9.601 | -7.769 | 1.05e-08 | 18.12 | LPP_vs_Control | ↓ | down | 2 | FFA_vs_Control, LPP_vs_Control | FFA_vs_Control: ↓ \| LPP_vs_Control: ↓ |
| SLC27A2 | -1.539 | 6.176 | -7.57 | 2.1e-08 | 17.07 | LPP_vs_Control | ↓ | down | 3 | CCCA_vs_Control, FFA_vs_Control, LPP_vs_Control | FFA_vs_Control: ↓ \| LPP_vs_Control: ↓ \| CCCA_vs_Control: ↓ |
| FAXDC2 | -0.98 | 10.202 | -7.238 | 5.43e-08 | 15.351 | LPP_vs_Control | ↓ | down | 3 | CCCA_vs_Control, FFA_vs_Control, LPP_vs_Control | FFA_vs_Control: ↓ \| LPP_vs_Control: ↓ \| CCCA_vs_Control: ↓ |
| ISM1 | -0.828 | 8.273 | -7.231 | 5.43e-08 | 15.315 | LPP_vs_Control | ↓ | down | 3 | CCCA_vs_Control, FFA_vs_Control, LPP_vs_Control | FFA_vs_Control: ↓ \| LPP_vs_Control: ↓ \| CCCA_vs_Control: ↓ |
| RORC | -0.701 | 8.046 | -7.223 | 5.43e-08 | 15.274 | LPP_vs_Control | ↓ | down | 1 | LPP_vs_Control | LPP_vs_Control: ↓ |
| GPHN | -0.564 | 7.749 | -7.208 | 5.43e-08 | 15.196 | LPP_vs_Control | ↓ | down | 1 | LPP_vs_Control | LPP_vs_Control: ↓ |
| ADGRG2 | -0.785 | 5.965 | -7.201 | 5.43e-08 | 15.16 | LPP_vs_Control | ↓ | down | 3 | CCCA_vs_Control, FFA_vs_Control, LPP_vs_Control | FFA_vs_Control: ↓ \| LPP_vs_Control: ↓ \| CCCA_vs_Control: ↓ |
| CYP39A1 | -0.93 | 7.871 | -7.192 | 5.43e-08 | 15.116 | LPP_vs_Control | ↓ | down | 1 | LPP_vs_Control | LPP_vs_Control: ↓ |
| ACY1 | -1.376 | 7.079 | -7.156 | 5.93e-08 | 14.932 | LPP_vs_Control | ↓ | down | 2 | FFA_vs_Control, LPP_vs_Control | FFA_vs_Control: ↓ \| LPP_vs_Control: ↓ |
| ABHD6 | -0.721 | 8.375 | -7.117 | 6.66e-08 | 14.731 | LPP_vs_Control | ↓ | down | 1 | LPP_vs_Control | LPP_vs_Control: ↓ |
| GATAD2B | -0.527 | 9.213 | -6.953 | 1.27e-07 | 13.901 | LPP_vs_Control | ↓ | down | 1 | LPP_vs_Control | LPP_vs_Control: ↓ |
| SC5D | -1.444 | 9.133 | -6.95 | 1.27e-07 | 13.888 | LPP_vs_Control | ↓ | down | 3 | CCCA_vs_Control, FFA_vs_Control, LPP_vs_Control | FFA_vs_Control: ↓ \| LPP_vs_Control: ↓ \| CCCA_vs_Control: ↓ |
| RGP1 | -0.538 | 9.046 | -6.939 | 1.27e-07 | 13.828 | LPP_vs_Control | ↓ | down | 2 | FFA_vs_Control, LPP_vs_Control | FFA_vs_Control: ↓ \| LPP_vs_Control: ↓ |
| ACOX2 | -1.028 | 7.862 | -6.937 | 1.27e-07 | 13.819 | LPP_vs_Control | ↓ | down | 3 | CCCA_vs_Control, FFA_vs_Control, LPP_vs_Control | FFA_vs_Control: ↓ \| LPP_vs_Control: ↓ \| CCCA_vs_Control: ↓ |
| RGMB | -1.067 | 8.337 | -6.916 | 1.33e-07 | 13.716 | LPP_vs_Control | ↓ | down | 1 | LPP_vs_Control | LPP_vs_Control: ↓ |
| MCCC2 | -0.822 | 8.91 | -6.888 | 1.38e-07 | 13.572 | LPP_vs_Control | ↓ | down | 3 | CCCA_vs_Control, FFA_vs_Control, LPP_vs_Control | FFA_vs_Control: ↓ \| LPP_vs_Control: ↓ \| CCCA_vs_Control: ↓ |
| ATP6V0C | -1.488 | 9.626 | -6.887 | 1.38e-07 | 13.569 | LPP_vs_Control | ↓ | down | 2 | FFA_vs_Control, LPP_vs_Control | FFA_vs_Control: ↓ \| LPP_vs_Control: ↓ |
| ZBED3 | -0.913 | 7.475 | -6.863 | 1.39e-07 | 13.451 | LPP_vs_Control | ↓ | down | 2 | FFA_vs_Control, LPP_vs_Control | FFA_vs_Control: ↓ \| LPP_vs_Control: ↓ |
| EIF1AD | -0.537 | 8.461 | -6.861 | 1.39e-07 | 13.438 | LPP_vs_Control | ↓ | down | 3 | CCCA_vs_Control, FFA_vs_Control, LPP_vs_Control | FFA_vs_Control: ↓ \| LPP_vs_Control: ↓ \| CCCA_vs_Control: ↓ |
| ALDH6A1 | -0.908 | 8.318 | -6.849 | 1.39e-07 | 13.381 | LPP_vs_Control | ↓ | down | 2 | CCCA_vs_Control, LPP_vs_Control | LPP_vs_Control: ↓ \| CCCA_vs_Control: ↓ |
| CCDC86 | -0.708 | 8.282 | -6.847 | 1.39e-07 | 13.368 | LPP_vs_Control | ↓ | down | 2 | FFA_vs_Control, LPP_vs_Control | FFA_vs_Control: ↓ \| LPP_vs_Control: ↓ |
| ACSS2 | -1.077 | 9.816 | -6.807 | 1.64e-07 | 13.167 | LPP_vs_Control | ↓ | down | 3 | CCCA_vs_Control, FFA_vs_Control, LPP_vs_Control | FFA_vs_Control: ↓ \| LPP_vs_Control: ↓ \| CCCA_vs_Control: ↓ |
| ZDHHC9 | -0.88 | 9.192 | -6.771 | 1.85e-07 | 12.991 | LPP_vs_Control | ↓ | down | 3 | CCCA_vs_Control, FFA_vs_Control, LPP_vs_Control | FFA_vs_Control: ↓ \| LPP_vs_Control: ↓ \| CCCA_vs_Control: ↓ |
| ACOT2 | -1.388 | 8.405 | -6.768 | 1.85e-07 | 12.976 | LPP_vs_Control | ↓ | down | 1 | LPP_vs_Control | LPP_vs_Control: ↓ |
| SYN2 | -0.823 | 6.554 | -6.743 | 1.95e-07 | 12.852 | LPP_vs_Control | ↓ | down | 1 | LPP_vs_Control | LPP_vs_Control: ↓ |
| IQCK | -0.849 | 7.064 | -6.726 | 2.06e-07 | 12.766 | LPP_vs_Control | ↓ | down | 1 | LPP_vs_Control | LPP_vs_Control: ↓ |
| AMACR | -1.271 | 6.691 | -6.718 | 2.07e-07 | 12.725 | LPP_vs_Control | ↓ | down | 1 | LPP_vs_Control | LPP_vs_Control: ↓ |
| GLYATL1 | -1.001 | 5.486 | -6.68 | 2.44e-07 | 12.539 | LPP_vs_Control | ↓ | down | 1 | LPP_vs_Control | LPP_vs_Control: ↓ |
| OTX1 | -0.776 | 7.7 | -6.668 | 2.51e-07 | 12.479 | LPP_vs_Control | ↓ | down | 1 | LPP_vs_Control | LPP_vs_Control: ↓ |
| PAFAH2 | -0.675 | 8.128 | -6.649 | 2.61e-07 | 12.384 | LPP_vs_Control | ↓ | down | 3 | CCCA_vs_Control, FFA_vs_Control, LPP_vs_Control | FFA_vs_Control: ↓ \| LPP_vs_Control: ↓ \| CCCA_vs_Control: ↓ |
| CCL27 | -2.152 | 6.296 | -6.648 | 2.61e-07 | 12.383 | LPP_vs_Control | ↓ | down | 1 | LPP_vs_Control | LPP_vs_Control: ↓ |
| BCKDHB | -0.824 | 8.176 | -6.628 | 2.78e-07 | 12.283 | LPP_vs_Control | ↓ | down | 3 | CCCA_vs_Control, FFA_vs_Control, LPP_vs_Control | FFA_vs_Control: ↓ \| LPP_vs_Control: ↓ \| CCCA_vs_Control: ↓ |
| ZNF43 | -0.87 | 8.337 | -6.625 | 2.78e-07 | 12.267 | LPP_vs_Control | ↓ | down | 2 | CCCA_vs_Control, LPP_vs_Control | LPP_vs_Control: ↓ \| CCCA_vs_Control: ↓ |
| SNAP29 | -0.589 | 8.837 | -6.6 | 3.07e-07 | 12.143 | LPP_vs_Control | ↓ | down | 2 | FFA_vs_Control, LPP_vs_Control | FFA_vs_Control: ↓ \| LPP_vs_Control: ↓ |
| CLDN8 | -1.058 | 7.595 | -6.595 | 3.07e-07 | 12.119 | LPP_vs_Control | ↓ | down | 1 | LPP_vs_Control | LPP_vs_Control: ↓ |
| SLC25A17 | -0.719 | 8.067 | -6.577 | 3.28e-07 | 12.033 | LPP_vs_Control | ↓ | down | 2 | CCCA_vs_Control, LPP_vs_Control | LPP_vs_Control: ↓ \| CCCA_vs_Control: ↓ |
| AGPAT3 | -1.097 | 9.84 | -6.553 | 3.61e-07 | 11.916 | LPP_vs_Control | ↓ | down | 3 | CCCA_vs_Control, FFA_vs_Control, LPP_vs_Control | FFA_vs_Control: ↓ \| LPP_vs_Control: ↓ \| CCCA_vs_Control: ↓ |
| SHMT1 | -0.502 | 8.7 | -6.482 | 5.06e-07 | 11.568 | LPP_vs_Control | ↓ | down | 1 | LPP_vs_Control | LPP_vs_Control: ↓ |
| NUDT7 | -0.725 | 6.567 | -6.478 | 5.06e-07 | 11.546 | LPP_vs_Control | ↓ | down | 1 | LPP_vs_Control | LPP_vs_Control: ↓ |
| AGPAT1 | -1.014 | 9.228 | -6.47 | 5.13e-07 | 11.51 | LPP_vs_Control | ↓ | down | 3 | CCCA_vs_Control, FFA_vs_Control, LPP_vs_Control | FFA_vs_Control: ↓ \| LPP_vs_Control: ↓ \| CCCA_vs_Control: ↓ |
| SGPP2 | -1.48 | 9.21 | -6.462 | 5.23e-07 | 11.47 | LPP_vs_Control | ↓ | down | 1 | LPP_vs_Control | LPP_vs_Control: ↓ |
| CERS6 | -1.296 | 8.666 | -6.453 | 5.29e-07 | 11.428 | LPP_vs_Control | ↓ | down | 1 | LPP_vs_Control | LPP_vs_Control: ↓ |
| CPT2 | -0.731 | 8.061 | -6.451 | 5.29e-07 | 11.415 | LPP_vs_Control | ↓ | down | 3 | CCCA_vs_Control, FFA_vs_Control, LPP_vs_Control | FFA_vs_Control: ↓ \| LPP_vs_Control: ↓ \| CCCA_vs_Control: ↓ |
| ACSM3 | -1.349 | 7.151 | -6.439 | 5.43e-07 | 11.359 | LPP_vs_Control | ↓ | down | 2 | CCCA_vs_Control, LPP_vs_Control | LPP_vs_Control: ↓ \| CCCA_vs_Control: ↓ |
| SLC31A2 | -1.811 | 8.418 | -6.437 | 5.43e-07 | 11.35 | LPP_vs_Control | ↓ | down | 1 | LPP_vs_Control | LPP_vs_Control: ↓ |
| WASL | -0.665 | 9.304 | -6.431 | 5.5e-07 | 11.318 | LPP_vs_Control | ↓ | down | 2 | FFA_vs_Control, LPP_vs_Control | FFA_vs_Control: ↓ \| LPP_vs_Control: ↓ |
| RNF185 | -0.62 | 9.233 | -6.418 | 5.74e-07 | 11.257 | LPP_vs_Control | ↓ | down | 1 | LPP_vs_Control | LPP_vs_Control: ↓ |
| ZNF675 | -0.862 | 7.484 | -6.41 | 5.88e-07 | 11.216 | LPP_vs_Control | ↓ | down | 2 | CCCA_vs_Control, LPP_vs_Control | LPP_vs_Control: ↓ \| CCCA_vs_Control: ↓ |
| HACL1 | -0.926 | 8.459 | -6.394 | 5.98e-07 | 11.14 | LPP_vs_Control | ↓ | down | 1 | LPP_vs_Control | LPP_vs_Control: ↓ |
| TMEM121 | 0.517 | 5.986 | 6.308 | 2.29e-06 | 10.715 | CCCA_vs_Control | ↑ | up | 1 | CCCA_vs_Control | CCCA_vs_Control: ↑ |
| ADRA2C | 0.895 | 6.924 | 6.198 | 3.16e-06 | 10.196 | CCCA_vs_Control | ↑ | up | 1 | CCCA_vs_Control | CCCA_vs_Control: ↑ |
| SNX22 | 0.929 | 7.0 | 5.903 | 6.11e-06 | 8.821 | CCCA_vs_Control | ↑ | up | 3 | CCCA_vs_Control, FFA_vs_Control, LPP_vs_Control | FFA_vs_Control: ↑ \| LPP_vs_Control: ↑ \| CCCA_vs_Control: ↑ |
| INHBA | 0.837 | 7.689 | 5.822 | 7.63e-06 | 8.451 | CCCA_vs_Control | ↑ | up | 2 | CCCA_vs_Control, LPP_vs_Control | LPP_vs_Control: ↑ \| CCCA_vs_Control: ↑ |
| SCARF2 | 0.639 | 7.146 | 5.457 | 2.24e-05 | 6.82 | CCCA_vs_Control | ↑ | up | 1 | CCCA_vs_Control | CCCA_vs_Control: ↑ |
| PRRX2 | 0.609 | 7.722 | 5.271 | 3.69e-05 | 6.015 | CCCA_vs_Control | ↑ | up | 1 | CCCA_vs_Control | CCCA_vs_Control: ↑ |
| PCDH17 | 0.516 | 6.296 | 5.252 | 3.93e-05 | 5.934 | CCCA_vs_Control | ↑ | up | 1 | CCCA_vs_Control | CCCA_vs_Control: ↑ |
| CLDN5 | 1.094 | 8.486 | 5.235 | 4.11e-05 | 5.863 | CCCA_vs_Control | ↑ | up | 1 | CCCA_vs_Control | CCCA_vs_Control: ↑ |
| TMEM204 | 0.637 | 7.718 | 5.222 | 4.26e-05 | 5.805 | CCCA_vs_Control | ↑ | up | 1 | CCCA_vs_Control | CCCA_vs_Control: ↑ |
| GJC2 | 0.572 | 6.242 | 5.204 | 4.41e-05 | 5.73 | CCCA_vs_Control | ↑ | up | 1 | CCCA_vs_Control | CCCA_vs_Control: ↑ |
| A4GALT | 0.71 | 7.637 | 5.203 | 4.41e-05 | 5.726 | CCCA_vs_Control | ↑ | up | 1 | CCCA_vs_Control | CCCA_vs_Control: ↑ |
| VWA1 | 0.555 | 8.497 | 5.17 | 4.84e-05 | 5.584 | CCCA_vs_Control | ↑ | up | 1 | CCCA_vs_Control | CCCA_vs_Control: ↑ |
| APC2 | 0.746 | 6.612 | 5.168 | 4.86e-05 | 5.577 | CCCA_vs_Control | ↑ | up | 2 | CCCA_vs_Control, LPP_vs_Control | LPP_vs_Control: ↑ \| CCCA_vs_Control: ↑ |
| ZNF775 | 0.71 | 7.276 | 5.157 | 5.06e-05 | 5.531 | CCCA_vs_Control | ↑ | up | 1 | CCCA_vs_Control | CCCA_vs_Control: ↑ |
| NRIP2 | 0.63 | 6.748 | 5.134 | 5.33e-05 | 5.432 | CCCA_vs_Control | ↑ | up | 2 | CCCA_vs_Control, LPP_vs_Control | LPP_vs_Control: ↑ \| CCCA_vs_Control: ↑ |
| ZNF787 | 0.538 | 7.89 | 5.112 | 5.66e-05 | 5.34 | CCCA_vs_Control | ↑ | up | 1 | CCCA_vs_Control | CCCA_vs_Control: ↑ |
| PKN1 | 0.686 | 8.505 | 5.11 | 5.68e-05 | 5.332 | CCCA_vs_Control | ↑ | up | 2 | CCCA_vs_Control, LPP_vs_Control | LPP_vs_Control: ↑ \| CCCA_vs_Control: ↑ |
| F12 | 0.572 | 6.892 | 5.102 | 5.79e-05 | 5.3 | CCCA_vs_Control | ↑ | up | 1 | CCCA_vs_Control | CCCA_vs_Control: ↑ |
| SEMA6B | 0.546 | 7.056 | 5.074 | 6.15e-05 | 5.181 | CCCA_vs_Control | ↑ | up | 1 | CCCA_vs_Control | CCCA_vs_Control: ↑ |
| METRN | 0.648 | 7.351 | 5.072 | 6.16e-05 | 5.173 | CCCA_vs_Control | ↑ | up | 1 | CCCA_vs_Control | CCCA_vs_Control: ↑ |
| GAS2L1 | 0.534 | 8.81 | 5.049 | 6.49e-05 | 5.078 | CCCA_vs_Control | ↑ | up | 1 | CCCA_vs_Control | CCCA_vs_Control: ↑ |
| EVA1B | 1.115 | 7.808 | 5.045 | 6.57e-05 | 5.064 | CCCA_vs_Control | ↑ | up | 2 | CCCA_vs_Control, LPP_vs_Control | LPP_vs_Control: ↑ \| CCCA_vs_Control: ↑ |
| EMILIN1 | 0.931 | 8.089 | 5.032 | 6.78e-05 | 5.008 | CCCA_vs_Control | ↑ | up | 2 | CCCA_vs_Control, LPP_vs_Control | LPP_vs_Control: ↑ \| CCCA_vs_Control: ↑ |
| G0S2 | 1.467 | 10.028 | 5.025 | 6.91e-05 | 4.979 | CCCA_vs_Control | ↑ | up | 2 | CCCA_vs_Control, LPP_vs_Control | LPP_vs_Control: ↑ \| CCCA_vs_Control: ↑ |
| TSSC4 | 0.528 | 8.293 | 4.951 | 8.58e-05 | 4.674 | CCCA_vs_Control | ↑ | up | 1 | CCCA_vs_Control | CCCA_vs_Control: ↑ |
| CYGB | 0.515 | 7.846 | 4.931 | 8.98e-05 | 4.592 | CCCA_vs_Control | ↑ | up | 1 | CCCA_vs_Control | CCCA_vs_Control: ↑ |
| PKDCC | 0.673 | 7.858 | 4.876 | 0.000105 | 4.37 | CCCA_vs_Control | ↑ | up | 2 | CCCA_vs_Control, LPP_vs_Control | LPP_vs_Control: ↑ \| CCCA_vs_Control: ↑ |
| TMEM140 | 0.545 | 7.31 | 4.862 | 0.000109 | 4.312 | CCCA_vs_Control | ↑ | up | 3 | CCCA_vs_Control, FFA_vs_Control, LPP_vs_Control | FFA_vs_Control: ↑ \| LPP_vs_Control: ↑ \| CCCA_vs_Control: ↑ |
| KLK14 | 0.651 | 6.568 | 4.861 | 0.000109 | 4.307 | CCCA_vs_Control | ↑ | up | 1 | CCCA_vs_Control | CCCA_vs_Control: ↑ |
| CCDC85B | 0.788 | 8.109 | 4.851 | 0.000111 | 4.267 | CCCA_vs_Control | ↑ | up | 1 | CCCA_vs_Control | CCCA_vs_Control: ↑ |
| EXOC3L1 | 0.637 | 6.877 | 4.828 | 0.000118 | 4.172 | CCCA_vs_Control | ↑ | up | 2 | CCCA_vs_Control, LPP_vs_Control | LPP_vs_Control: ↑ \| CCCA_vs_Control: ↑ |
| ZBTB20 | 1.276 | 8.829 | 4.826 | 0.000118 | 4.167 | CCCA_vs_Control | ↑ | up | 2 | CCCA_vs_Control, LPP_vs_Control | LPP_vs_Control: ↑ \| CCCA_vs_Control: ↑ |
| C22orf23 | 1.1 | 6.682 | 4.815 | 0.000121 | 4.12 | CCCA_vs_Control | ↑ | up | 2 | CCCA_vs_Control, LPP_vs_Control | LPP_vs_Control: ↑ \| CCCA_vs_Control: ↑ |
| HAGHL | 0.598 | 6.994 | 4.791 | 0.000128 | 4.026 | CCCA_vs_Control | ↑ | up | 1 | CCCA_vs_Control | CCCA_vs_Control: ↑ |
| MMP17 | 0.567 | 7.121 | 4.768 | 0.000134 | 3.935 | CCCA_vs_Control | ↑ | up | 1 | CCCA_vs_Control | CCCA_vs_Control: ↑ |
| IL3RA | 0.721 | 7.164 | 4.723 | 0.000152 | 3.753 | CCCA_vs_Control | ↑ | up | 2 | CCCA_vs_Control, LPP_vs_Control | LPP_vs_Control: ↑ \| CCCA_vs_Control: ↑ |
| PERM1 | 1.531 | 7.064 | 4.71 | 0.000158 | 3.702 | CCCA_vs_Control | ↑ | up | 2 | CCCA_vs_Control, LPP_vs_Control | LPP_vs_Control: ↑ \| CCCA_vs_Control: ↑ |
| IGSF22 | 0.751 | 6.328 | 4.707 | 0.000159 | 3.693 | CCCA_vs_Control | ↑ | up | 1 | CCCA_vs_Control | CCCA_vs_Control: ↑ |
| CDC42EP5 | 0.832 | 6.933 | 4.705 | 0.00016 | 3.684 | CCCA_vs_Control | ↑ | up | 1 | CCCA_vs_Control | CCCA_vs_Control: ↑ |
| PITX1 | 0.852 | 6.779 | 4.669 | 0.000176 | 3.543 | CCCA_vs_Control | ↑ | up | 1 | CCCA_vs_Control | CCCA_vs_Control: ↑ |
| HYAL1 | 0.591 | 7.89 | 4.654 | 0.000183 | 3.482 | CCCA_vs_Control | ↑ | up | 1 | CCCA_vs_Control | CCCA_vs_Control: ↑ |
| PWWP2B | 0.505 | 7.878 | 4.637 | 0.000188 | 3.418 | CCCA_vs_Control | ↑ | up | 1 | CCCA_vs_Control | CCCA_vs_Control: ↑ |
| MXRA8 | 0.676 | 9.599 | 4.634 | 0.00019 | 3.403 | CCCA_vs_Control | ↑ | up | 2 | CCCA_vs_Control, LPP_vs_Control | LPP_vs_Control: ↑ \| CCCA_vs_Control: ↑ |
| IGFL1 | 0.999 | 6.296 | 4.614 | 0.0002 | 3.328 | CCCA_vs_Control | ↑ | up | 2 | CCCA_vs_Control, LPP_vs_Control | LPP_vs_Control: ↑ \| CCCA_vs_Control: ↑ |
| PTGIR | 0.581 | 7.303 | 4.606 | 0.000206 | 3.295 | CCCA_vs_Control | ↑ | up | 1 | CCCA_vs_Control | CCCA_vs_Control: ↑ |
| CRTAC1 | 0.511 | 6.048 | 4.603 | 0.000207 | 3.283 | CCCA_vs_Control | ↑ | up | 1 | CCCA_vs_Control | CCCA_vs_Control: ↑ |
| SLC51A | 0.723 | 6.282 | 4.583 | 0.000216 | 3.206 | CCCA_vs_Control | ↑ | up | 3 | CCCA_vs_Control, FFA_vs_Control, LPP_vs_Control | FFA_vs_Control: ↑ \| LPP_vs_Control: ↑ \| CCCA_vs_Control: ↑ |
| GATA2 | 0.752 | 7.625 | 4.582 | 0.000217 | 3.2 | CCCA_vs_Control | ↑ | up | 1 | CCCA_vs_Control | CCCA_vs_Control: ↑ |
| SOD3 | 0.827 | 9.241 | 4.577 | 0.000219 | 3.182 | CCCA_vs_Control | ↑ | up | 1 | CCCA_vs_Control | CCCA_vs_Control: ↑ |
| SLC1A7 | 0.689 | 6.128 | 4.564 | 0.000228 | 3.133 | CCCA_vs_Control | ↑ | up | 1 | CCCA_vs_Control | CCCA_vs_Control: ↑ |
| FAXDC2 | -1.253 | 10.202 | -7.92 | 9.06e-09 | 18.859 | CCCA_vs_Control | ↓ | down | 3 | CCCA_vs_Control, FFA_vs_Control, LPP_vs_Control | FFA_vs_Control: ↓ \| LPP_vs_Control: ↓ \| CCCA_vs_Control: ↓ |
| ACSL5 | -1.207 | 7.724 | -7.093 | 3.5e-07 | 14.577 | CCCA_vs_Control | ↓ | down | 1 | CCCA_vs_Control | CCCA_vs_Control: ↓ |
| ISM1 | -0.942 | 8.273 | -7.049 | 3.5e-07 | 14.355 | CCCA_vs_Control | ↓ | down | 3 | CCCA_vs_Control, FFA_vs_Control, LPP_vs_Control | FFA_vs_Control: ↓ \| LPP_vs_Control: ↓ \| CCCA_vs_Control: ↓ |
| SLC27A2 | -1.642 | 6.176 | -6.913 | 4.51e-07 | 13.674 | CCCA_vs_Control | ↓ | down | 3 | CCCA_vs_Control, FFA_vs_Control, LPP_vs_Control | FFA_vs_Control: ↓ \| LPP_vs_Control: ↓ \| CCCA_vs_Control: ↓ |
| ACOX2 | -1.195 | 7.862 | -6.905 | 4.51e-07 | 13.631 | CCCA_vs_Control | ↓ | down | 3 | CCCA_vs_Control, FFA_vs_Control, LPP_vs_Control | FFA_vs_Control: ↓ \| LPP_vs_Control: ↓ \| CCCA_vs_Control: ↓ |
| TLR5 | -0.686 | 7.696 | -6.766 | 7.77e-07 | 12.943 | CCCA_vs_Control | ↓ | down | 1 | CCCA_vs_Control | CCCA_vs_Control: ↓ |
| ADGRG2 | -0.854 | 5.965 | -6.706 | 9.13e-07 | 12.645 | CCCA_vs_Control | ↓ | down | 3 | CCCA_vs_Control, FFA_vs_Control, LPP_vs_Control | FFA_vs_Control: ↓ \| LPP_vs_Control: ↓ \| CCCA_vs_Control: ↓ |
| ZNF675 | -1.049 | 7.484 | -6.677 | 9.28e-07 | 12.503 | CCCA_vs_Control | ↓ | down | 2 | CCCA_vs_Control, LPP_vs_Control | LPP_vs_Control: ↓ \| CCCA_vs_Control: ↓ |
| ALCAM | -1.122 | 8.788 | -6.635 | 1.03e-06 | 12.295 | CCCA_vs_Control | ↓ | down | 1 | CCCA_vs_Control | CCCA_vs_Control: ↓ |
| TMEM164 | -1.366 | 9.811 | -6.543 | 1.49e-06 | 11.847 | CCCA_vs_Control | ↓ | down | 2 | CCCA_vs_Control, FFA_vs_Control | FFA_vs_Control: ↓ \| CCCA_vs_Control: ↓ |
| PKIB | -1.073 | 6.27 | -6.504 | 1.65e-06 | 11.659 | CCCA_vs_Control | ↓ | down | 1 | CCCA_vs_Control | CCCA_vs_Control: ↓ |
| PAFAH2 | -0.768 | 8.128 | -6.475 | 1.75e-06 | 11.519 | CCCA_vs_Control | ↓ | down | 3 | CCCA_vs_Control, FFA_vs_Control, LPP_vs_Control | FFA_vs_Control: ↓ \| LPP_vs_Control: ↓ \| CCCA_vs_Control: ↓ |
| HSBP1L1 | -0.615 | 8.71 | -6.427 | 2.04e-06 | 11.285 | CCCA_vs_Control | ↓ | down | 1 | CCCA_vs_Control | CCCA_vs_Control: ↓ |
| HLCS | -0.978 | 7.939 | -6.415 | 2.04e-06 | 11.23 | CCCA_vs_Control | ↓ | down | 1 | CCCA_vs_Control | CCCA_vs_Control: ↓ |
| EIF1AD | -0.584 | 8.461 | -6.383 | 2.23e-06 | 11.076 | CCCA_vs_Control | ↓ | down | 3 | CCCA_vs_Control, FFA_vs_Control, LPP_vs_Control | FFA_vs_Control: ↓ \| LPP_vs_Control: ↓ \| CCCA_vs_Control: ↓ |
| AGPAT1 | -1.165 | 9.228 | -6.37 | 2.23e-06 | 11.011 | CCCA_vs_Control | ↓ | down | 3 | CCCA_vs_Control, FFA_vs_Control, LPP_vs_Control | FFA_vs_Control: ↓ \| LPP_vs_Control: ↓ \| CCCA_vs_Control: ↓ |
| SC5D | -1.538 | 9.133 | -6.339 | 2.23e-06 | 10.865 | CCCA_vs_Control | ↓ | down | 3 | CCCA_vs_Control, FFA_vs_Control, LPP_vs_Control | FFA_vs_Control: ↓ \| LPP_vs_Control: ↓ \| CCCA_vs_Control: ↓ |
| ZNF43 | -0.972 | 8.337 | -6.339 | 2.23e-06 | 10.863 | CCCA_vs_Control | ↓ | down | 2 | CCCA_vs_Control, LPP_vs_Control | LPP_vs_Control: ↓ \| CCCA_vs_Control: ↓ |
| KNSTRN | -1.134 | 7.962 | -6.33 | 2.23e-06 | 10.819 | CCCA_vs_Control | ↓ | down | 2 | CCCA_vs_Control, FFA_vs_Control | FFA_vs_Control: ↓ \| CCCA_vs_Control: ↓ |
| ACAA2 | -1.373 | 9.282 | -6.328 | 2.23e-06 | 10.809 | CCCA_vs_Control | ↓ | down | 1 | CCCA_vs_Control | CCCA_vs_Control: ↓ |
| PLIN2 | -1.474 | 9.682 | -6.303 | 2.29e-06 | 10.694 | CCCA_vs_Control | ↓ | down | 1 | CCCA_vs_Control | CCCA_vs_Control: ↓ |
| DHRS9 | -1.23 | 7.437 | -6.284 | 2.42e-06 | 10.6 | CCCA_vs_Control | ↓ | down | 1 | CCCA_vs_Control | CCCA_vs_Control: ↓ |
| CLMP | -1.4 | 9.355 | -6.27 | 2.48e-06 | 10.536 | CCCA_vs_Control | ↓ | down | 1 | CCCA_vs_Control | CCCA_vs_Control: ↓ |
| SLC25A17 | -0.8 | 8.067 | -6.262 | 2.48e-06 | 10.496 | CCCA_vs_Control | ↓ | down | 2 | CCCA_vs_Control, LPP_vs_Control | LPP_vs_Control: ↓ \| CCCA_vs_Control: ↓ |
| BCKDHB | -0.901 | 8.176 | -6.204 | 3.16e-06 | 10.224 | CCCA_vs_Control | ↓ | down | 3 | CCCA_vs_Control, FFA_vs_Control, LPP_vs_Control | FFA_vs_Control: ↓ \| LPP_vs_Control: ↓ \| CCCA_vs_Control: ↓ |
| NTAN1 | -1.26 | 8.898 | -6.181 | 3.33e-06 | 10.112 | CCCA_vs_Control | ↓ | down | 1 | CCCA_vs_Control | CCCA_vs_Control: ↓ |
| ZNF136 | -0.629 | 7.026 | -6.165 | 3.39e-06 | 10.039 | CCCA_vs_Control | ↓ | down | 1 | CCCA_vs_Control | CCCA_vs_Control: ↓ |
| ACSM3 | -1.507 | 7.151 | -6.163 | 3.39e-06 | 10.028 | CCCA_vs_Control | ↓ | down | 2 | CCCA_vs_Control, LPP_vs_Control | LPP_vs_Control: ↓ \| CCCA_vs_Control: ↓ |
| AGPAT3 | -1.202 | 9.84 | -6.15 | 3.45e-06 | 9.969 | CCCA_vs_Control | ↓ | down | 3 | CCCA_vs_Control, FFA_vs_Control, LPP_vs_Control | FFA_vs_Control: ↓ \| LPP_vs_Control: ↓ \| CCCA_vs_Control: ↓ |
| MCCC2 | -0.856 | 8.91 | -6.147 | 3.45e-06 | 9.952 | CCCA_vs_Control | ↓ | down | 3 | CCCA_vs_Control, FFA_vs_Control, LPP_vs_Control | FFA_vs_Control: ↓ \| LPP_vs_Control: ↓ \| CCCA_vs_Control: ↓ |
| ALDH6A1 | -0.95 | 8.318 | -6.134 | 3.52e-06 | 9.893 | CCCA_vs_Control | ↓ | down | 2 | CCCA_vs_Control, LPP_vs_Control | LPP_vs_Control: ↓ \| CCCA_vs_Control: ↓ |
| ADGRL3 | -1.381 | 8.912 | -6.13 | 3.52e-06 | 9.874 | CCCA_vs_Control | ↓ | down | 1 | CCCA_vs_Control | CCCA_vs_Control: ↓ |
| ABHD5 | -1.629 | 9.637 | -6.117 | 3.56e-06 | 9.812 | CCCA_vs_Control | ↓ | down | 1 | CCCA_vs_Control | CCCA_vs_Control: ↓ |
| MUC1 | -2.022 | 7.801 | -6.114 | 3.56e-06 | 9.801 | CCCA_vs_Control | ↓ | down | 2 | CCCA_vs_Control, FFA_vs_Control | FFA_vs_Control: ↓ \| CCCA_vs_Control: ↓ |
| PPFIA1 | -0.758 | 9.492 | -6.111 | 3.56e-06 | 9.784 | CCCA_vs_Control | ↓ | down | 1 | CCCA_vs_Control | CCCA_vs_Control: ↓ |
| ACSS2 | -1.124 | 9.816 | -6.082 | 4e-06 | 9.65 | CCCA_vs_Control | ↓ | down | 3 | CCCA_vs_Control, FFA_vs_Control, LPP_vs_Control | FFA_vs_Control: ↓ \| LPP_vs_Control: ↓ \| CCCA_vs_Control: ↓ |
| TM7SF3 | -0.772 | 9.151 | -6.065 | 4.24e-06 | 9.571 | CCCA_vs_Control | ↓ | down | 1 | CCCA_vs_Control | CCCA_vs_Control: ↓ |
| ZDHHC9 | -0.918 | 9.192 | -6.05 | 4.45e-06 | 9.5 | CCCA_vs_Control | ↓ | down | 3 | CCCA_vs_Control, FFA_vs_Control, LPP_vs_Control | FFA_vs_Control: ↓ \| LPP_vs_Control: ↓ \| CCCA_vs_Control: ↓ |
| MGST1 | -1.561 | 10.373 | -6.025 | 4.82e-06 | 9.382 | CCCA_vs_Control | ↓ | down | 1 | CCCA_vs_Control | CCCA_vs_Control: ↓ |
| TRIM24 | -0.575 | 8.036 | -6.022 | 4.82e-06 | 9.368 | CCCA_vs_Control | ↓ | down | 1 | CCCA_vs_Control | CCCA_vs_Control: ↓ |
| GABRA4 | -1.166 | 6.169 | -6.011 | 4.82e-06 | 9.318 | CCCA_vs_Control | ↓ | down | 1 | CCCA_vs_Control | CCCA_vs_Control: ↓ |
| GATA6 | -1.004 | 7.058 | -6.011 | 4.82e-06 | 9.318 | CCCA_vs_Control | ↓ | down | 2 | CCCA_vs_Control, FFA_vs_Control | FFA_vs_Control: ↓ \| CCCA_vs_Control: ↓ |
| CPT2 | -0.795 | 8.061 | -6.002 | 4.82e-06 | 9.277 | CCCA_vs_Control | ↓ | down | 3 | CCCA_vs_Control, FFA_vs_Control, LPP_vs_Control | FFA_vs_Control: ↓ \| LPP_vs_Control: ↓ \| CCCA_vs_Control: ↓ |
| MOSPD2 | -0.843 | 8.44 | -6.001 | 4.82e-06 | 9.272 | CCCA_vs_Control | ↓ | down | 1 | CCCA_vs_Control | CCCA_vs_Control: ↓ |
| OLAH | -1.598 | 5.759 | -5.972 | 5.27e-06 | 9.138 | CCCA_vs_Control | ↓ | down | 1 | CCCA_vs_Control | CCCA_vs_Control: ↓ |
| BEAN1 | -1.207 | 7.538 | -5.97 | 5.27e-06 | 9.129 | CCCA_vs_Control | ↓ | down | 2 | CCCA_vs_Control, FFA_vs_Control | FFA_vs_Control: ↓ \| CCCA_vs_Control: ↓ |
| TMPRSS11E | -1.647 | 8.637 | -5.964 | 5.27e-06 | 9.102 | CCCA_vs_Control | ↓ | down | 1 | CCCA_vs_Control | CCCA_vs_Control: ↓ |
| PDZK1 | -2.132 | 6.986 | -5.96 | 5.27e-06 | 9.083 | CCCA_vs_Control | ↓ | down | 2 | CCCA_vs_Control, FFA_vs_Control | FFA_vs_Control: ↓ \| CCCA_vs_Control: ↓ |
| ZNF667 | -0.588 | 7.437 | -5.958 | 5.27e-06 | 9.071 | CCCA_vs_Control | ↓ | down | 1 | CCCA_vs_Control | CCCA_vs_Control: ↓ |
| ZNF189 | -0.752 | 8.176 | -5.956 | 5.27e-06 | 9.065 | CCCA_vs_Control | ↓ | down | 1 | CCCA_vs_Control | CCCA_vs_Control: ↓ |

#### ****S7. Differential Abundance of Inferred Cell Types across Scarring Alopecia Subtypes****

To explore disease-specific alterations in tissue composition, we performed transcriptome-based cell-type deconvolution on all included datasets. We identified significant differences in inferred cell abundances between scarring alopecia subtypes (CCCA, LPP, FFA) and healthy scalp controls (Supplementary **Table S16**). These results revealed both common and subtype-specific changes in the immune and stromal cellular microenvironment.

Several cell types were consistently altered across all three subtypes. **Adipocytes**, **stromal cells** (StromaScore), and the **overall microenvironment score** were significantly increased in CCCA, LPP, and FFA, suggesting common stromal expansion or dermal remodeling. Notably, **fibroblasts** were increased in CCCA and FFA but decreased in LPP, while **plasmacytoid dendritic cells (pDCs)**, **pro-B cells**, **naive B cells**, and **plasma cells** followed a shared pattern of elevation in CCCA and reduction in LPP and FFA, implying divergent B-cell lineage involvement.

Subtype-specific signatures included:

- In **CCCA**, increased abundance of **monocytes**, **NK cells**, and **B-cell subsets**, along with decreased **M2 macrophages**, **Th2**, and **CD8+ naive T cells**, reflecting a mixed inflammatory and reparative profile.
- **LPP** was characterized by increased **eosinophils**, **endothelial cells**, and **T effector memory cells**, alongside decreased **Tregs**, **cDCs**, and **GMPs**, indicating robust immune activation and vascular remodeling.
- **FFA** showed significant depletion of **M1 macrophages**, **CD4+ naive T cells**, **sebocytes**, and **memory B cells**, suggesting reduced tissue-resident or progenitor cell populations in this subtype.

These inferred shifts in cell populations add a mechanistic layer to the transcriptomic signatures observed in the GSVA and DEG analyses, underscoring both shared immune dysregulation and subtype-specific microenvironmental remodeling across primary lymphocytic scarring alopecias.

****Supplementary Table S12. Differential Abundance of Inferred Cell Types in Primary Lymphocytic Scarring Alopecias Compared to Healthy Controls****

This table summarises the results of the deconvolution analysis comparing inferred cell type abundance across three primary lymphocytic scarring alopecia (PLSA) subtypes—central centrifugal cicatricial alopecia (CCCA), frontal fibrosing alopecia (FFA), and lichen planopilaris (LPP)—relative to healthy scalp samples. Only cell types with statistically significant differences (adjusted p < 0.05) in at least one disease group are included.

Each row denotes a specific cell type, the number of disease groups in which it showed a significant difference (n), the disease groups affected, the direction of change relative to controls (↑ = increased, ↓ = decreased, --- = not significant), and the minimum adjusted p-value observed across the groups.

Coloured arrows indicate directionality:

- **Red arrows (↑)** indicate significantly increased abundance compared to healthy controls.
- **Blue arrows (↓)** indicate significantly decreased abundance.
- **Black dashes (---)** indicate non-significant differences in that group.

This summary highlights shared and disease-specific patterns of immune and stromal cell dysregulation across the PLSA spectrum.

| **CellType** | **n** | **Groups** | **Direction** | **Min p-value** |
| --- | --- | --- | --- | --- |
| **Shared by all types** |  |  |  |  |
| Adipocytes | 3 | **CCCA, LPP, FFA** | ↑, ↑, ↑ | 2.28e-5 |
| StromaScore | 3 | **CCCA, LPP, FFA** | ↑, ↑, ↑ | 2.95e-3 |
| MicroenvironmentScore | 3 | **CCCA, LPP, FFA** | ↑, ↑, ↑ | 5.18e-5 |
| Fibroblasts | 3 | **CCCA, LPP, FFA** | ↑, ↓, ↑ | 1.06e-5 |
| pDC | 3 | **CCCA, LPP, FFA** | ↑, ↓, ↓ | 2.71e-9 |
| pro B-cells | 3 | **CCCA, LPP, FFA** | ↑, ↓, ↓ | 1.62e-5 |
| naive B-cells | 3 | **CCCA, LPP, FFA** | ↑, ↓, ↓ | 7.30e-5 |
| Plasma cells | 3 | **CCCA, LPP, FFA** | ↑, ↓, ↓ | 2.98e-6 |
| CMP | 3 | **CCCA, LPP, FFA** | ↓, ↓, ↑ | 2.45e-3 |
| **Shared by two** |  |  |  |  |
| Preadipocytes | 2 | **---, LPP, FFA** | ↑, ↑ | 5.65e-6 |
| Mast cells | 2 | **---, LPP, FFA** | ↑, ↑ | 3.14e-5 |
| CD8^+^ T-cells | 2 | **---, LPP, FFA** | ↑, ↑ | 2.58e-4 |
| CD4^+^ TCM cells | 2 | **---, LPP, FFA** | ↑, ↑ | 3.74e-5 |
| ImmuneScore | 2 | **CCCA**, ---, **FFA** | ↑, ↑ | 1.85e-3 |
| Class-switched memory B-cells | 2 | **CCCA**, ---, **FFA** | ↑, ↑ | 1.68e-4 |
| CLP | 2 | **---, LPP, FFA** | ↓, ↓ | 4.25e-6 |
| Basophils | 2 | **---, LPP, FFA** | ↓, ↓ | 5.20e-6 |
| Smooth muscle | 2 | **---, LPP, FFA** | ↓, ↓ | 3.63e-5 |
| Melanocytes | 2 | **CCCA, ---, FFA** | ↑, ↓ | 6.27e-5 |
| Myocytes | 2 | **---, LPP, FFA** | ↓, ↓ | 6.13e-4 |
| Neurons | 2 | **---, LPP, FFA** | ↓, ↓ | 6.86e-4 |
| Tgd cells | 2 | **---, LPP, FFA** | ↓, ↓ | 1.38e-3 |
| **Only CCCA** |  |  |  |  |
| Monocytes | 1 | **CCCA** | ↑ | 1.98e-3 |
| B-cells | 1 | **CCCA** | ↑ | 8.11e-3 |
| NK cells | 1 | **CCCA** | ↑ | 8.69e-3 |
| CD8+ naive T-cells | 1 | **CCCA** | ↓ | 2.57e-3 |
| Th2 cells | 1 | **CCCA** | ↓ | 4.39e-3 |
| Macrophages M2 | 1 | **CCCA** | ↓ | 1.47e-2 |
| Megakaryocytes | 1 | **CCCA** | ↓ | 2.05e-2 |
| iDC | 1 | **CCCA** | ↓ | 2.73e-2 |
| Pericytes | 1 | **CCCA** | ↓ | 2.89e-2 |
| CD4+ memory T-cells | 1 | **CCCA** | ↓ | 3.92e-2 |
| **Only LPP** |  |  |  |  |
| Eosinophils | 1 | **LPP** | ↑ | 3.85e-4 |
| Microvascular Endothelial cells | 1 | **LPP** | ↑ | 7.34e-3 |
| Epithelial cells | 1 | **LPP** | ↑ | 1.54e-2 |
| CD4+ Tem | 1 | **LPP** | ↑ | 3.83e-2 |
| Tregs | 1 | **LPP** | ↓ | 7.27e-3 |
| cDC | 1 | **LPP** | ↓ | 1.19e-2 |
| GMP | 1 | **LPP** | ↓ | 1.21e-2 |
| DC | 1 | **LPP** | ↓ | 3.10e-2 |
| **Only FFA** |  |  |  |  |
| Macrophages M1 | 1 | **FFA** | ↓ | 9.21e-4 |
| CD4^+^ naive T-cells | 1 | **FFA** | ↓ | 1.02e-3 |
| Memory B-cells | 1 | **FFA** | ↓ | 1.41e-3 |
| Sebocytes | 1 | **FFA** | ↓ | 3.90e-3 |
| MSC | 1 | **FFA** | ↓ | 5.39e-3 |

**S8. Drug Repurposing**

**Table S13. Drug repurposing predictions and prioritised candidates**

This table summarises the top repositioning candidates across alopecia subtypes, ranked by number of differentially expressed target genes and adjusted p-values. No candidates were identified in CCCA. All entries correspond to upregulated gene signatures in FFA or LPP. Groups of special interest such as anti-inflammatory agents, fibrates, JAK inhibitors, antioxidants, and antidiabetics are highlighted in bold.

| **Drug** | **Functional Group** | **Genes Found** | **Adj.P-val** | **Targeted genes** | **Gene expression** | **Alopecia Subtype** | **Database Source** |
| --- | --- | --- | --- | --- | --- | --- | --- |
| **infliximab** | Anti-inflammatory / immunomodulators | 4 | 9.92e-03 | HLA-B, CD40, HLA-C, PTPRC | UP | FFA | PharmGKB |
| Tumor necrosis factor alpha (TNF-alpha) inhibitors | Anti-inflammatory / immunomodulators | 4 | 1.11e-02 | TAP1, CD40, HLA-C, PTPRC | UP | FFA | PharmGKB |
| staurosporine | Others / unclear | 3 | 4.49e-02 | LCK, ZAP70, ITK | UP | FFA | LINCS |
| **peginterferon alfa-2b** | Anti-inflammatory / immunomodulators | 3 | 1.11e-02 | HLA-B, HLA-C, CXCL10 | UP | FFA | PharmGKB |
| **etanercept** | Anti-inflammatory / immunomodulators | 3 | 2.94e-02 | HLA-B, CD40, PTPRC | UP | FFA | PharmGKB |
| Rubella vaccines | Others / unclear | 2 | 3.24e-03 | HLA-B, HLA-C | UP | FFA | PharmGKB |
| methazolamide | Others / unclear | 2 | 4.84e-03 | HLA-B, HLA-C | UP | FFA | PharmGKB |
| ticlopidine | Others / unclear | 2 | 1.11e-02 | HLA-B, HLA-C | UP | FFA | PharmGKB |
| clavulanate | Others / unclear | 2 | 1.11e-02 | HLA-B, HLA-C | UP | FFA | PharmGKB |
| sulfamethoxazole / trimethoprim | Antibiotics / antimicrobials | 2 | 1.11e-02 | HLA-B, HLA-C | UP | FFA | PharmGKB |
| flucloxacillin | Antibiotics / antimicrobials | 2 | 1.27e-02 | HLA-B, HLA-C | UP | FFA | PharmGKB |
| glatiramer acetate | Anti-inflammatory / immunomodulators | 2 | 1.57e-02 | CCR5, IL7R | UP | FFA | PharmGKB |
| amoxicillin | Antibiotics / antimicrobials | 2 | 1.57e-02 | HLA-B, HLA-C | UP | FFA | PharmGKB |
| lapatinib | Others / unclear | 2 | 2.71e-02 | HLA-B, HLA-C | UP | FFA | PharmGKB |
| **peginterferon alfa-2a** | Anti-inflammatory / immunomodulators | 2 | 3.64e-02 | HLA-B, CXCL10 | UP | FFA | PharmGKB |
| nevirapine | Antibiotics / antimicrobials | 2 | 4.29e-02 | HLA-B, HLA-C | UP | FFA | PharmGKB |
| **ribavirin** | Anti-inflammatory / immunomodulators | 2 | 4.42e-02 | HLA-B, HLA-C | UP | FFA | PharmGKB |
| lamotrigine | Antiepileptics / CNS | 2 | 4.56e-02 | HLA-B, HLA-C | UP | FFA | PharmGKB |
| allopurinol | Others / unclear | 2 | 4.74e-02 | HLA-B, HLA-C | UP | FFA | PharmGKB |
| **ciprofibrate** | Fibrates / lipid regulators | 1 | 3.83e-02 | LPL | UP | Shared | LINCS |
| oxybenzone | Others / unclear | 1 | 3.83e-02 | LIPE | UP | Shared | LINCS |
| **clofibrate** | Fibrates / lipid regulators | 1 | 3.83e-02 | LPL | UP | Shared | LINCS |
| **niacin** | Antidiabetics / metabolic | 1 | 4.73e-02 | NNMT | UP | Shared | LINCS |
| mepacrine | Others / unclear | 1 | 4.73e-02 | PLA2G2A | UP | Shared | LINCS |
| RHC-80267 | Others / unclear | 1 | 4.73e-02 | LIPE | UP | Shared | LINCS |
| orphenadrine | Others / unclear | 1 | 4.73e-02 | GRIN3B | UP | Shared | LINCS |
| atomoxetine | Others / unclear | 1 | 4.73e-02 | GRIN3B | UP | Shared | LINCS |
| **bezafibrate** | Fibrates / lipid regulators | 1 | 4.73e-02 | LPL | UP | Shared | LINCS |
| milnacipran | Others / unclear | 1 | 4.73e-02 | GRIN3B | UP | Shared | LINCS |
| **indometacin** | NSAIDs | 1 | 4.73e-02 | PLA2G2A | UP | Shared | LINCS |
| **gemfibrozil** | Fibrates / lipid regulators | 1 | 4.73e-02 | LPL | UP | Shared | LINCS |
| BIIB021 | Others / unclear | 1 | 3.26e-02 | AOX1 | UP | LPP | LINCS |
| **parecoxib** | NSAIDs | 1 | 3.26e-02 | LTF | UP | LPP | LINCS |
| **nimesulide** | NSAIDs | 1 | 3.26e-02 | LTF | UP | LPP | LINCS |
| brimonidine | Antihypertensives / vasodilators | 1 | 3.26e-02 | AOX1 | UP | LPP | LINCS |
| targinine | Others / unclear | 1 | 3.26e-02 | NOS3 | UP | LPP | LINCS |
| 7-nitroindazole | Others / unclear | 1 | 3.26e-02 | NOS3 | UP | LPP | LINCS |
| **methotrexate** | Anti-inflammatory / immunomodulators | 1 | 3.26e-02 | AOX1 | UP | LPP | LINCS |
| PD-166793 | Anti-inflammatory / immunomodulators | 1 | 3.26e-02 | MMP3 | UP | LPP | LINCS |
| **mefloquine** | Others / unclear | 1 | 3.26e-02 | HBA1 | UP | LPP | LINCS |
| UK-356618 | Others / unclear | 1 | 3.32e-02 | MMP3 | UP | LPP | LINCS |
| **tetrahydrobiopterin** | Antidiabetics / metabolic | 1 | 3.32e-02 | NOS3 | UP | LPP | LINCS |
| **nicotinamide** | Antioxidants / vitamins | 1 | 3.65e-02 | AOX1 | UP | LPP | LINCS |
| proadifen | Others / unclear | 1 | 2.83e-02 | NOS1 | DOWN | LPP | LINCS |
| cimetidine | Others / unclear | 1 | 2.83e-02 | SLC47A1 | DOWN | LPP | LINCS |
| diethylstilbestrol | Hormonal / nuclear receptor modulators | 1 | 2.83e-02 | ESRRG | DOWN | LPP | LINCS |
| 7-nitroindazole | Others / unclear | 1 | 2.83e-02 | NOS1 | DOWN | LPP | LINCS |
| bisphenol-a | Hormonal / nuclear receptor modulators | 1 | 2.83e-02 | ESRRG | DOWN | LPP | LINCS |
| DY-131 | Hormonal / nuclear receptor modulators | 1 | 2.83e-02 | ESRRG | DOWN | LPP | LINCS |
| **azathioprine** | Anti-inflammatory / immunomodulators | 1 | 2.83e-02 | HPRT1 | DOWN | LPP | LINCS |
| daidzein | Hormonal / nuclear receptor modulators | 1 | 2.83e-02 | ESRRG | DOWN | LPP | LINCS |
| pancuronium | Others / unclear | 1 | 2.83e-02 | CHRNA1 | DOWN | LPP | LINCS |
| mercaptopurine | Others / unclear | 1 | 2.83e-02 | HPRT1 | DOWN | LPP | LINCS |
| AR-C133057XX | Others / unclear | 1 | 2.83e-02 | NOS1 | DOWN | LPP | LINCS |
| pyrimethamine | Others / unclear | 1 | 3.24e-02 | SLC47A1 | DOWN | LPP | LINCS |
| **JAK3-inhibitor-VI** | JAK inhibitors | 1 | 4.49e-02 | JAK3 | UP | FFA | LINCS |
| vicriviroc | Others / unclear | 1 | 4.49e-02 | CCR5 | UP | FFA | LINCS |
| aminogenistein | JAK inhibitors | 1 | 4.49e-02 | LCK | UP | FFA | LINCS |
| **JAK3-inhibitor-I** | JAK inhibitors | 1 | 4.49e-02 | JAK3 | UP | FFA | LINCS |
| KIN001-127 | JAK inhibitors | 1 | 4.49e-02 | ITK | UP | FFA | LINCS |
| JW-7-24-1 | JAK inhibitors | 1 | 4.49e-02 | LCK | UP | FFA | LINCS |
| **JAK3-inhibitor-V** | JAK inhibitors | 1 | 4.49e-02 | JAK3 | UP | FFA | LINCS |
| zidovudine / lamivudine / abacavir | Antiepileptics / CNS | 1 | 2.94e-02 | HLA-B | UP | FFA | PharmGKB |
| dolutegravir / abacavir / lamivudine | Antiepileptics / CNS | 1 | 2.94e-02 | HLA-B | UP | FFA | PharmGKB |
| acetazolamide | Antiepileptics / CNS | 1 | 2.94e-02 | HLA-B | UP | FFA | PharmGKB |
| trichloroethylene | Others / unclear | 1 | 2.94e-02 | HLA-B | UP | FFA | PharmGKB |
| penicillin g | Antibiotics / antimicrobials | 1 | 2.94e-02 | HLA-B | UP | FFA | PharmGKB |
| lamivudine / abacavir | Antiepileptics / CNS | 1 | 2.94e-02 | HLA-B | UP | FFA | PharmGKB |
| avelumab | Others / unclear | 1 | 2.94e-02 | CD274 | UP | FFA | PharmGKB |
| carbimazole | Antiepileptics / CNS | 1 | 4.29e-02 | HLA-B | UP | FFA | PharmGKB |
| maraviroc | Others / unclear | 1 | 4.29e-02 | CCR5 | UP | FFA | PharmGKB |
| cabotegravir / rilpivirine | Others / unclear | 1 | 4.29e-02 | HLA-B | UP | FFA | PharmGKB |
| penicillin v | Antibiotics / antimicrobials | 1 | 4.29e-02 | HLA-B | UP | FFA | PharmGKB |
| **belimumab** | Anti-inflammatory / immunomodulators | 1 | 4.29e-02 | TNFSF13B | UP | FFA | PharmGKB |
| minocycline | Antibiotics / antimicrobials | 1 | 4.29e-02 | HLA-B | UP | FFA | PharmGKB |
| fosphenytoin | Antiepileptics / CNS | 1 | 4.74e-02 | HLA-B | UP | FFA | PharmGKB |
| clindamycin | Antibiotics / antimicrobials | 1 | 4.74e-02 | HLA-B | UP | FFA | PharmGKB |
| propylthiouracil | Others / unclear | 1 | 4.74e-02 | HLA-B | UP | FFA | PharmGKB |
| methimazole | Others / unclear | 1 | 4.74e-02 | HLA-B | UP | FFA | PharmGKB |
| tremelimumab | Anti-inflammatory / immunomodulators | 1 | 4.74e-02 | CD274 | UP | FFA | PharmGKB |
| nivolumab / relatlimab | Anti-inflammatory / immunomodulators | 1 | 4.74e-02 | CD274 | UP | FFA | PharmGKB |
| **Antihypertensives And Diuretics In Combination** | Antihypertensives / vasodilators | 1 | 4.40e-02 | NOS3 | UP | LPP | PharmGKB |
| XK469 | Others / unclear | 1 | 4.40e-02 | AOX1 | UP | LPP | PharmGKB |
| **l-arginine** | Antidiabetics / metabolic | 1 | 4.40e-02 | NOS3 | UP | LPP | PharmGKB |

**S9. Assessment of Riks of Bias and Conflicts of Interest across studies**

**We systematically evaluated the methodological quality and risk of bias of all included datasets using a ROBINS-I framework adapted for transcriptomic studies. Each study was assessed across six domains: sample selection, platform consistency, metadata transparency, control comparability, conflict of interest (COI) disclosure, and peer-review status. The full scoring and rationale for each dataset are presented in Supplementary Table S5.**

Most datasets exhibited **low risk of bias** in terms of **platform consistency**, with all studies using internally consistent and technically validated platforms—either RNA-seq (GSE186075, GSE125733) or Affymetrix microarrays (GSE113052, GSE179054, GSE59131, GSE58934). An exception was **GSE11905**, which used an outdated low-coverage Operon v2 21k array, leading to a **high risk** rating for platform consistency.

In terms of **sample selection**, five datasets (GSE186075, GSE125733, GSE113052, GSE179054) demonstrated **low risk**, reflecting well-defined inclusion criteria and appropriate disease classification. **GSE11905**, **GSE59131**, and **GSE58934** were rated as **moderate risk** due to limited or absent information regarding participant recruitment.

**Metadata transparency** varied across studies. Only **GSE179054** achieved a **low risk** rating, offering detailed sample-level metadata including clinical severity categorization. All other datasets provided minimal to moderate sample annotation. Notably, **GSE59131** and **GSE58934** lacked critical clinical variables such as age, sex, or disease duration, and were rated **moderate risk** accordingly.

**Control comparability** emerged as the most variable and concerning domain. Studies using **individual healthy controls** (e.g., GSE186075, GSE125733) were rated as **low risk**. In contrast, **GSE113052** and **GSE179054** employed intra-individual comparisons without external healthy controls, yielding **high risk** for this domain. **GSE59131** and **GSE58934** used pooled controls (n=10), a design that obscures interindividual variability and introduces potential bias, also rated as **high risk**.

Regarding **COI disclosure** and **peer-review status**, all published datasets (n=5) included appropriate funding and conflict of interest declarations. However, **GSE59131** and **GSE58934** lacked associated peer-reviewed publications or public disclosures and were thus flagged as **"No"** for both domains.

In summary, while most datasets presented acceptable methodological quality for integration into a meta-analytic framework, two studies (**GSE59131**, **GSE58934**) exhibited multiple domains with elevated or unclear risk, including the use of pooled controls and absence of peer-reviewed reporting. These limitations were explicitly addressed in sensitivity analyses and flagged in interpretation of results.

**Supplementary Table S14. Risk of Bias Assessment of Included Transcriptomic Datasets (Adapted ROBINS-I Framework)**

This table presents the study-level risk of bias assessment for each transcriptomic dataset included in the meta-analysis. An adapted version of the ROBINS-I tool was applied across six domains: **sample selection**, **platform consistency**, **metadata transparency**, **control comparability**, **conflict of interest (COI) disclosure**, and **peer-reviewed publication status**. Risk levels were rated as low, moderate, or high for each domain.

Definitions of risk levels:

- **Sample Selection**: Appropriateness of case/control definitions and sample representativeness.
- **Platform Consistency**: Use of validated and uniform gene expression profiling technologies.
- **Metadata Transparency**: Availability of essential sample-level metadata (e.g., age, sex, phenotype).
- **Control Comparability**: Appropriateness and consistency of control groups.
- **COI Disclosure**: Whether conflicts of interest and funding sources were reported.
- **Peer-reviewed**: Whether the dataset is linked to a peer-reviewed publication.

Evaluations were performed independently by two reviewers, with discrepancies resolved by consensus.

| **Study** | **Sample Selection** | **Platform Consistency** | **Metadata Transparency** | **Control Comparability** | **COI Disclosure** | **Peer-reviewed** |
| --- | --- | --- | --- | --- | --- | --- |
| GSE186075 | Low risk – Well-defined inclusion of LPP, FFA, and CCCA samples with clearly labeled individual controls. | Low risk – RNA-seq (HiSeq 3000) was used uniformly across samples. | Moderate – Basic metadata (group, sample type) were available; clinical variables such as age or disease duration were lacking. | Low risk – Individual healthy control samples matched by tissue type. | Yes – Conflicts of interest and funding sources declared in the associated publication. | Yes |
| GSE125733 | Low risk – Included well-characterized FFA cases and individual controls. | Low risk – Uniform use of RNA-seq (HiSeq 2000). | Moderate risk – Sample identifiers and groupings were available, but limited clinical detail. | Low risk – Controls were individual scalp samples and appropriately assigned. | Yes – Conflicts of interest and funding sources declared in the associated publication. | Yes |
| GSE113052 | Low risk – Robust within-subject design comparing lesional and non-lesional samples from the same CCCA patients. | Low risk – All samples processed using Affymetrix Clariom S arrays. | Moderate risk – Groupings were clear but clinical descriptors were limited. | High risk – No external healthy controls; comparisons restricted to intra-individual analysis. | Yes – Conflicts of interest and funding sources declared in the associated publication. | Yes |
| GSE179054 | Low risk – Clear classification of CCCA patients into severity categories (focal, limited, extensive). | Low risk – Samples processed using Affymetrix Clariom S platform. | Low risk - Included sample-level metadata and severity categorization. | High risk – No external healthy controls; comparisons restricted to intra-individual analysis. | Yes – Conflicts of interest and funding sources declared in the associated publication. | Yes |
| GSE11905 | Moderate risk – Included a mix of scarring alopecia subtypes (LPP, PsPB) without consistent recruitment criteria. | High risk – Used an outdated and low-coverage microarray platform (Operon v2 21k). | Moderate risk – Minimal metadata available; only basic group assignment. | Moderate risk – Included healthy controls but with unclear matching. | Yes – Conflicts of interest and funding sources declared in the associated publication. | Yes |
| GSE59131 | Moderate risk – Included CCCA and LPP samples, but unclear recruitment methodology. | Low risk – All samples were processed using the same standardized microarray platform (Affymetrix U133 Plus 2.0). | Moderate risk – Groupings were clear but clinical descriptors were limited. | High risk – Used pooled controls (mix of 10 individuals), which may reduce variance representation. | No | No |
| GSE58934 | Moderate risk – Included FFA samples and pooled controls; recruitment details not provided. | Low risk – All samples were processed using the same standardized microarray platform (Affymetrix U133 Plus 2.0). | *Moderate risk* - Groupings were clear but clinical descriptors were limited. | High risk – Used pooled controls (mix of 10 individuals), which may reduce variance representation. | No | No |

### **S10. Meta-analysis of GSVA Functional Pathways Using a Leave-One-Study-Out Framework**

To evaluate the robustness of functional enrichment differences across primary scarring alopecia (PLSAs) subtypes, we conducted a leave-one-study-out (LOSO) sensitivity meta-analysis using GSVA-derived scores for 33 immune and epithelial-related pathways. Meta-analytic results were computed separately for each disease comparison—central centrifugal cicatricial alopecia (CCCA), frontal fibrosing alopecia (FFA), and lichen planopilaris (LPP)—against controls, applying a random-effects model across datasets.

**Supplementary Table S14** summarizes, for each functional pathway and comparison, the number of studies included (n), the mean log2-fold change in enrichment scores, and the corresponding 95% confidence interval (CI). Pathways with statistically significant differences (i.e., 95% CI not overlapping zero) are highlighted in bold. The results revealed both shared and disease-specific functional signatures.

#### *Shared Immune Activation in PLSAs Subtypes*

Several pathways demonstrated consistent enrichment across all alopecia subtypes, supporting common immune mechanisms. These included:

- **B Cells**, **Cytotoxicity**, **Dendritic Cells**, **Macrophages**, **Memory T Cells**, **Mast Cells**, **Monocytes**, and **NK Cells**, all of which were significantly enriched in at least two comparisons and showed strong robustness under LOSO analysis.
- **Type I Interferon Response**, **T Cell Exhaustion**, and **Fibroblasts** also exhibited significant and stable enrichment in all subtypes, aligning with previously reported immune-inflammatory mechanisms in PSA pathophysiology.

#### *Subtype-Specific Signatures*

- **FFA** was characterized by the strongest upregulation of immune pathways, with marked enrichment in **Th1**, **Type I IFN**, **Dendritic Cells**, and **Cytotoxicity** pathways (e.g., Th1: logFC = 0.629 [0.425, 0.833], p < 0.001).
- **LPP** showed enrichment in **EMT**, **JAK/STAT**, and **Hair Follicle Immune Privilege** (HFIP), with significant meta-analytic logFC values and minimal sensitivity to individual study removal.
- **CCCA** showed lower effect sizes across most pathways, though significant upregulation was detected for **Fibroblasts**, **Mast Cells**, **Monocytes**, **Tregs**, and **Th1**, as well as downregulation of **Senescence** and **Follicular Keratins**. These results suggest a distinct fibroinflammatory and immunoregulatory profile.

#### *Robustness of Findings*

To assess the influence of individual studies, **Supplementary Table S15** (detailed LOSO results) reports logFC and p-values for the meta-analysis and for each dataset excluded. Pathways with stable direction and statistical significance upon exclusion of any single dataset included:

- **Cytotoxicity**, **Fibroblasts**, **Th1**, **NK Cells**, and **Type I IFN Response** in FFA and LPP.
- **Mast Cells**, **Dendritic Cells**, **Monocytes**, and **Tregs** in CCCA.

These patterns highlight robust and reproducible transcriptomic alterations underpinning both shared and divergent mechanisms in PSA subtypes.

**Supplementary Table S15. Meta-Analysis of GSVA Pathway Enrichment in Scarring Alopecia Subtypes Compared to Healthy Controls. Each cell reports the meta-analytic results for GSVA enrichment scores corresponding to one functional pathway and disease comparison. The format is: n;meanlogFC(95%CI)**, where: ***n** = number of studies included in the meta-analysis for that comparison, ****mean logFC** = mean log2-fold change of GSVA scores between the disease group and controls, and *****95% CI** = confidence interval of the mean effect size. Bold entries denote statistically significant results (i.e., 95% CI excludes zero). The analysis was performed using random-effects meta-analysis across datasets, applying a Leave-One-Study-Out (LOSO) framework.

| **Functional Pathway** | **CCCA vs Control (n=5)** | **FFA vs Control (n=6)** | **LPP vs Control (n=5)** |
| --- | --- | --- | --- |
| Apoptosis | 0.111 (-0.176, 0.398) | 0.024 (-0.275, 0.323) | 0.155 (-0.152, 0.462) |
| B_Cells | 0.357 (-0.008, 0.723) | 0.13 (-0.142, 0.403) | **0.458 (0.117, 0.799)** |
| Cellular_Stress | -0.04 (-0.401, 0.322) | 0.048 (-0.256, 0.352) | -0.042 (-0.382, 0.298) |
| Cornified_Envelope | 0.22 (-0.294, 0.733) | 0.153 (-0.386, 0.691) | -0.01 (-0.482, 0.462) |
| Cytotoxicity | 0.441 (-0.082, 0.964) | 0.253 (-0.151, 0.658) | 0.647 (0.206, 1.087) |
| DNA_Damage | 0.107 (-0.22, 0.434) | -0.122 (-0.429, 0.185) | 0.178 (-0.145, 0.501) |
| Dendritic_Cells | 0.337 (-0.074, 0.748) | 0.207 (-0.127, 0.541) | **0.471 (0.085, 0.857)** |
| EMT | 0.204 (-0.162, 0.57) | 0.107(-0.225, 0.44) | 0.209 (-0.129, 0.546) |
| Epidermal_Keratins | -0.09 (-0.458, 0.278) | 0.044(-0.38, 0.469) | -0.117 (-0.509, 0.275) |
| Fibroblasts | **0.468 (0.077, 0.86)** | **0.385 (0.033, 0.738)** | 0.32 (-0.05, 0.69) |
| Fibrosis_TGFB_Signaling | 0.35 (-0.037, 0.737) | 0.296(-0.073, 0.666) | 0.207 (-0.145, 0.558) |
| Follicle_Structure | -0.141 (-0.632, 0.35) | -0.238(-0.612, 0.136) | -0.104 (-0.574, 0.366) |
| Follicular_Keratins | -0.009 (-0.656, 0.638) | -0.225(-0.737,0.287) | 0.005 (-0.606, 0.617) |
| HF_Immune_Privilege | 0.238 (-0.127, 0.603) | 0.056(-0.239,0.351) | **0.411 (0.045, 0.776)** |
| JAK_STAT_Pathway | 0.253 (-0.178, 0.684) | 0.058(-0.316,0.432) | 0.315 (-0.081, 0.711) |
| Keratinocytes | 0.025 (-0.312, 0.361) | -0.031(-0.38,0.318) | -0.046 (-0.369, 0.276) |
| MAIT_Cells | 0.331 (-0.073, 0.734) | 0.139(-0.164,0.442) | **0.479 (0.12, 0.838)** |
| Macrophages | 0.306 (-0.028, 0.64) | 0.146(-0.116,0.408) | **0.403 (0.094, 0.711)** |
| Mast_Cells | **0.412 (0.071, 0.753)** | **0.344(0.05,0.638)** | **0.412 (0.082, 0.743)** |
| Memory_T_Cells | 0.346 (0.0, 0.692) | 0.223(-0.051,0.497) | **0.494 (0.16, 0.828)** |
| Monocytes | **0.392 (0.022, 0.762)** | 0.184(-0.124,0.493) | **0.47 (0.113, 0.828)** |
| NK_Cells | **0.44 (0.046, 0.834)** | 0.266(-0.067,0.6) | **0.54 (0.178, 0.902)** |
| Neutrophils | **0.393 (0.112, 0.675)** | 0.258(-0.007,0.524) | **0.357 (0.074, 0.641)** |
| Senescence | -0.072 (-0.344, 0.201) | -0.182(-0.413,0.048) | -0.112 (-0.383, 0.16) |
| Skin_Immune_Cells | 0.293 (-0.154, 0.74) | 0.086(-0.273,0.444) | **0.516 (0.093, 0.938)** |
| T_cell_exhaustion | 0.276 (-0.138, 0.69) | 0.218(-0.091,0.526) | **0.405 (0.02, 0.79)** |
| Th1 | 0.425 (-0.097, 0.946) | 0.217(-0.211,0.644) | **0.598 (0.13, 1.066)** |
| Th17 | 0.103 (-0.291, 0.496) | 0.061(-0.293,0.414) | 0.137 (-0.225, 0.498) |
| Th2 | -0.097 (-0.41, 0.216) | -0.098(-0.441,0.245) | 0.078 (-0.232, 0.387) |
| Th22 | -0.342 (-0.716, 0.033) | -0.307(-0.742,0.129) | -0.135 (-0.511, 0.242) |
| Th9 | 0.021 (-0.396, 0.439) | 0.143(-0.232,0.518) | 0.142 (-0.263, 0.547) |
| Tregs | 0.197 (-0.205, 0.598) | 0.207(-0.106,0.52) | **0.397 (0.012, 0.783)** |
| Type_I_IFN_Response | 0.401 (-0.055, 0.857) | 0.192(-0.222,0.607) | **0.534 (0.089, 0.979)** |
| Wnt_Signaling | 0.015 (-0.25, 0.28) | -0.001(-0.241,0.239) | 0.004 (-0.259, 0.267) |
| gd_T_Cells | 0.259 (-0.23, 0.748) | 0.091(-0.28,0.461) | **0.491 (0.061, 0.921)** |

**Supplementary Table S16. Impact of Study Exclusion on Functional Enrichment Patterns in Scarring Alopecias: LOSO Meta-Analysis of GSVA Scores**

**This table summarises the results of LOSO random-effects meta-analysis of GSVA enrichment scores for curated functional pathways across three primary lymphocytic scarring alopecia (PLSA) subtypes—CCCA, FFA, and LPP—compared to healthy controls. Each cell reports the number of contributing studies (**n**) and the mean log₂ fold-change (logFC) in GSVA scores with the corresponding 95% confidence interval (CI), in the format:** n; mean logFC (95% CI)**. Bold values indicate statistically significant results (i.e., 95% CI excludes zero).**

| **Comparison** | **Functional Pathway** | **Excluded Study** | **logFC (95% CI); p-value** |
| --- | --- | --- | --- |
| CCCA_vs_Control | **Apoptosis** | Meta-analysis | 0.027 (-0.095, 0.148); p = 0.668 |
|  |  | GSE113052 | 0.055 (-0.242, 0.351); p = 0.718 |
|  |  | GSE125733 | 0.071 (-0.204, 0.345); p = 0.614 |
|  |  | GSE179054 | 0.037 (-0.261, 0.334); p = 0.809 |
|  |  | GSE186075 | 0.013 (-0.314, 0.340); p = 0.939 |
|  |  | GSE58934 | 0.007 (-0.278, 0.291); p = 0.964 |
|  |  | GSE59131 | -0.037 (-0.352, 0.278); p = 0.818 |
| CCCA_vs_Control | **B_Cells** | **Meta-analysis** | **0.136 (0.027, 0.246); p = 0.015** |
|  |  | GSE113052 | 0.127 (-0.134, 0.389); p = 0.340 |
|  |  | GSE125733 | 0.197 (-0.029, 0.422); p = 0.087 |
|  |  | GSE179054 | 0.139 (-0.161, 0.439); p = 0.365 |
|  |  | GSE186075 | 0.096 (-0.206, 0.398); p = 0.532 |
|  |  | GSE58934 | 0.124 (-0.142, 0.389); p = 0.360 |
|  |  | GSE59131 | 0.099 (-0.182, 0.381); p = 0.489 |
| CCCA_vs_Control | **Cellular_Stress** | Meta-analysis | 0.052 (-0.071, 0.175); p = 0.407 |
|  |  | GSE113052 | 0.078 (-0.226, 0.381); p = 0.617 |
|  |  | GSE125733 | 0.040 (-0.249, 0.329); p = 0.786 |
|  |  | GSE179054 | 0.038 (-0.306, 0.383); p = 0.827 |
|  |  | GSE186075 | -0.037 (-0.355, 0.282); p = 0.821 |
|  |  | GSE58934 | 0.068 (-0.229, 0.365); p = 0.652 |
|  |  | GSE59131 | 0.102 (-0.170, 0.374); p = 0.463 |
| CCCA_vs_Control | **Cornified_Envelope** | Meta-analysis | 0.162 (-0.056, 0.381); p = 0.145 |
|  |  | GSE113052 | 0.158 (-0.405, 0.721); p = 0.582 |
|  |  | GSE125733 | 0.185 (-0.342, 0.711); p = 0.492 |
|  |  | GSE179054 | 0.226 (-0.261, 0.713); p = 0.362 |
|  |  | GSE186075 | -0.028 (-0.628, 0.573); p = 0.928 |
|  |  | GSE58934 | 0.219 (-0.294, 0.731); p = 0.403 |
|  |  | GSE59131 | 0.156 (-0.386, 0.699); p = 0.573 |
| CCCA_vs_Control | **Cytotoxicity** | **Meta-analysis** | **0.262 (0.098, 0.425); p = 0.002** |
|  |  | GSE113052 | 0.307 (-0.097, 0.711); p = 0.136 |
|  |  | **GSE125733** | **0.347 (0.004, 0.690); p = 0.048** |
|  |  | GSE179054 | 0.256 (-0.179, 0.691); p = 0.249 |
|  |  | GSE186075 | 0.157 (-0.289, 0.603); p = 0.489 |
|  |  | GSE58934 | 0.230 (-0.162, 0.621); p = 0.250 |
|  |  | GSE59131 | 0.222 (-0.186, 0.630); p = 0.285 |
| CCCA_vs_Control | **DNA_Damage** | **Meta-analysis** | **-0.127 (-0.252, -0.002); p = 0.047** |
|  |  | GSE113052 | -0.148 (-0.454, 0.158); p = 0.344 |
|  |  | GSE125733 | -0.141 (-0.436, 0.155); p = 0.351 |
|  |  | GSE179054 | -0.155 (-0.475, 0.165); p = 0.342 |
|  |  | GSE186075 | 0.049 (-0.276, 0.374); p = 0.769 |
|  |  | GSE58934 | -0.136 (-0.438, 0.165); p = 0.376 |
|  |  | GSE59131 | -0.202 (-0.494, 0.090); p = 0.175 |
| CCCA_vs_Control | **Dendritic_Cells** | **Meta-analysis** | **0.215 (0.080, 0.350); p = 0.002** |
|  |  | GSE113052 | 0.227 (-0.106, 0.561); p = 0.182 |
|  |  | **GSE125733** | **0.286 (0.007, 0.565); p = 0.045** |
|  |  | GSE179054 | 0.226 (-0.130, 0.582); p = 0.214 |
|  |  | GSE186075 | 0.111 (-0.250, 0.472); p = 0.548 |
|  |  | GSE58934 | 0.203 (-0.123, 0.530); p = 0.222 |
|  |  | GSE59131 | 0.189 (-0.161, 0.540); p = 0.289 |
| CCCA_vs_Control | **EMT** | Meta-analysis | 0.112 (-0.023, 0.247); p = 0.104 |
|  |  | GSE113052 | 0.087 (-0.246, 0.421); p = 0.607 |
|  |  | GSE125733 | 0.143 (-0.158, 0.444); p = 0.351 |
|  |  | GSE179054 | 0.135 (-0.192, 0.462); p = 0.419 |
|  |  | GSE186075 | 0.020 (-0.349, 0.390); p = 0.914 |
|  |  | GSE58934 | 0.145 (-0.172, 0.462); p = 0.370 |
|  |  | GSE59131 | 0.113 (-0.235, 0.460); p = 0.526 |
| CCCA_vs_Control | **Epidermal_Keratins** | Meta-analysis | 0.052 (-0.121, 0.225); p = 0.556 |
|  |  | GSE113052 | 0.049 (-0.397, 0.495); p = 0.829 |
|  |  | GSE125733 | 0.062 (-0.351, 0.476); p = 0.767 |
|  |  | GSE179054 | 0.098 (-0.306, 0.503); p = 0.635 |
|  |  | GSE186075 | -0.167 (-0.628, 0.294); p = 0.477 |
|  |  | GSE58934 | 0.097 (-0.307, 0.502); p = 0.638 |
|  |  | GSE59131 | 0.126 (-0.291, 0.543); p = 0.555 |
| CCCA_vs_Control | **Fibroblasts** | **Meta-analysis** | **0.394 (0.252, 0.536); p = 0.000** |
|  |  | **GSE113052** | **0.407 (0.058, 0.757); p = 0.022** |
|  |  | **GSE125733** | **0.449 (0.153, 0.746); p = 0.003** |
|  |  | **GSE179054** | **0.433 (0.066, 0.801); p = 0.021** |
|  |  | GSE186075 | 0.216 (-0.173, 0.604); p = 0.276 |
|  |  | **GSE58934** | **0.395 (0.049, 0.741); p = 0.025** |
|  |  | **GSE59131** | **0.413 (0.044, 0.782); p = 0.028** |
| CCCA_vs_Control | **Fibrosis_TGFB_Signaling** | **Meta-analysis** | **0.302 (0.152, 0.451); p = 0.000** |
|  |  | GSE113052 | 0.289 (-0.072, 0.650); p = 0.117 |
|  |  | **GSE125733** | **0.345 (0.019, 0.670); p = 0.038** |
|  |  | *GSE179054* | *0.358 (-0.020, 0.735); p = 0.063* |
|  |  | GSE186075 | 0.176 (-0.238, 0.590); p = 0.405 |
|  |  | GSE58934 | 0.298 (-0.064, 0.659); p = 0.107 |
|  |  | GSE59131 | 0.311 (-0.066, 0.688); p = 0.106 |
| CCCA_vs_Control | **Follicle_Structure** | **Meta-analysis** | **-0.238 (-0.390, -0.086); p = 0.002** |
|  |  | GSE113052 | -0.272 (-0.640, 0.096); p = 0.147 |
|  |  | GSE125733 | -0.284 (-0.633, 0.065); p = 0.111 |
|  |  | GSE179054 | -0.244 (-0.656, 0.169); p = 0.247 |
|  |  | GSE186075 | -0.212 (-0.603, 0.178); p = 0.287 |
|  |  | GSE58934 | -0.187 (-0.534, 0.160); p = 0.290 |
|  |  | GSE59131 | -0.228 (-0.605, 0.148); p = 0.234 |
| CCCA_vs_Control | **Follicular_Keratins** | **Meta-analysis** | **-0.224 (-0.431, -0.017); p = 0.034** |
|  |  | GSE59131 | -0.218 (-0.702, 0.266); p = 0.377 |
|  |  | GSE113052 | -0.254 (-0.750, 0.242); p = 0.315 |
|  |  | GSE125733 | -0.265 (-0.741, 0.212); p = 0.276 |
|  |  | GSE179054 | -0.236 (-0.809, 0.337); p = 0.419 |
|  |  | GSE186075 | -0.220 (-0.786, 0.346); p = 0.446 |
|  |  | GSE58934 | -0.155 (-0.631, 0.321); p = 0.524 |
| CCCA_vs_Control | **HF_Immune_Privilege** | Meta-analysis | 0.068 (-0.050, 0.186); p = 0.258 |
|  |  | GSE113052 | 0.099 (-0.187, 0.385); p = 0.498 |
|  |  | GSE125733 | 0.139 (-0.093, 0.371); p = 0.240 |
|  |  | GSE179054 | 0.076 (-0.245, 0.397); p = 0.641 |
|  |  | GSE186075 | -0.057 (-0.381, 0.268); p = 0.732 |
|  |  | GSE58934 | 0.051 (-0.237, 0.338); p = 0.729 |
|  |  | GSE59131 | 0.028 (-0.290, 0.346); p = 0.863 |
| CCCA_vs_Control | **JAK_STAT_Pathway** | Meta-analysis | 0.061 (-0.091, 0.213); p = 0.428 |
|  |  | GSE113052 | 0.022 (-0.354, 0.399); p = 0.908 |
|  |  | GSE125733 | 0.129 (-0.203, 0.461); p = 0.446 |
|  |  | GSE179054 | 0.090 (-0.303, 0.483); p = 0.654 |
|  |  | GSE186075 | 0.004 (-0.387, 0.395); p = 0.982 |
|  |  | GSE58934 | 0.050 (-0.316, 0.415); p = 0.789 |
|  |  | GSE59131 | 0.052 (-0.335, 0.438); p = 0.793 |
| CCCA_vs_Control | **Keratinocytes** | Meta-analysis | -0.028 (-0.169, 0.114); p = 0.702 |
|  |  | GSE113052 | -0.046 (-0.397, 0.306); p = 0.799 |
|  |  | GSE125733 | -0.024 (-0.364, 0.316); p = 0.890 |
|  |  | GSE179054 | -0.006 (-0.335, 0.323); p = 0.971 |
|  |  | GSE186075 | -0.081 (-0.480, 0.317); p = 0.689 |
|  |  | GSE58934 | 0.012 (-0.317, 0.342); p = 0.943 |
|  |  | GSE59131 | -0.041 (-0.384, 0.303); p = 0.817 |
| CCCA_vs_Control | **MAIT_Cells** | **Meta-analysis** | **0.146 (0.024, 0.268); p = 0.019** |
|  |  | GSE113052 | 0.149 (-0.149, 0.448); p = 0.327 |
|  |  | GSE125733 | 0.212 (-0.037, 0.461); p = 0.095 |
|  |  | GSE179054 | 0.151 (-0.178, 0.481); p = 0.368 |
|  |  | GSE186075 | 0.108 (-0.227, 0.442); p = 0.528 |
|  |  | GSE58934 | 0.120 (-0.173, 0.412); p = 0.423 |
|  |  | GSE59131 | 0.094 (-0.221, 0.409); p = 0.558 |
| CCCA_vs_Control | **Macrophages** | **Meta-analysis** | **0.151 (0.046, 0.257); p = 0.005** |
|  |  | GSE113052 | 0.154 (-0.107, 0.415); p = 0.247 |
|  |  | GSE125733 | *0.206 (-0.015, 0.428); p = 0.068* |
|  |  | GSE179054 | 0.150 (-0.119, 0.419); p = 0.274 |
|  |  | GSE186075 | 0.102 (-0.190, 0.394); p = 0.493 |
|  |  | GSE58934 | 0.132 (-0.120, 0.385); p = 0.305 |
|  |  | GSE59131 | 0.131 (-0.145, 0.407); p = 0.351 |
| CCCA_vs_Control | **Mast_Cells** | **Meta-analysis** | **0.350 (0.231, 0.469); p = 0.000** |
|  |  | **GSE113052** | **0.405 (0.119, 0.691); p = 0.006** |
|  |  | **GSE125733** | **0.400 (0.145, 0.654); p = 0.002** |
|  |  | **GSE179054** | **0.338 (0.014, 0.663); p = 0.041** |
|  |  | GSE186075 | 0.185 (-0.122, 0.492); p = 0.237 |
|  |  | **GSE58934** | **0.355 (0.065, 0.645); p = 0.016** |
|  |  | **GSE59131** | **0.383 (0.081, 0.685); p = 0.013** |
| CCCA_vs_Control | **Memory_T_Cells** | **Meta-analysis** | **0.228 (0.118, 0.339); p = 0.000** |
|  |  | GSE113052 | 0.240 (-0.030, 0.510); p = 0.081 |
|  |  | **GSE125733** | **0.280 (0.050, 0.510); p = 0.017** |
|  |  | GSE179054 | 0.243 (-0.047, 0.533); p = 0.101 |
|  |  | GSE186075 | 0.170 (-0.127, 0.468); p = 0.262 |
|  |  | GSE58934 | 0.211 (-0.055, 0.477); p = 0.120 |
|  |  | GSE59131 | 0.193 (-0.100, 0.485); p = 0.196 |
| CCCA_vs_Control | **Monocytes** | **Meta-analysis** | **0.192 (0.067, 0.317); p = 0.003** |
|  |  | GSE113052 | 0.226 (-0.075, 0.527); p = 0.142 |
|  |  | GSE125733 | *0.251 (-0.016, 0.518); p = 0.066* |
|  |  | GSE179054 | 0.212 (-0.104, 0.527); p = 0.189 |
|  |  | GSE186075 | 0.086 (-0.255, 0.427); p = 0.622 |
|  |  | GSE58934 | 0.190 (-0.111, 0.491); p = 0.215 |
|  |  | GSE59131 | 0.141 (-0.186, 0.468); p = 0.399 |
| CCCA_vs_Control | **NK_Cells** | **Meta-analysis** | **0.272 (0.137, 0.407); p = 0.000** |
|  |  | **GSE113052** | **0.331 (0.007, 0.654); p = 0.045** |
|  |  | **GSE125733** | **0.325 (0.030, 0.620); p = 0.031** |
|  |  | GSE179054 | 0.278 (-0.079, 0.635); p = 0.127 |
|  |  | GSE186075 | 0.163 (-0.204, 0.530); p = 0.384 |
|  |  | GSE58934 | 0.250 (-0.074, 0.574); p = 0.130 |
|  |  | GSE59131 | 0.252 (-0.082, 0.586); p = 0.139 |
| CCCA_vs_Control | **Neutrophils** | **Meta-analysis** | **0.262 (0.154, 0.370); p = 0.000** |
|  |  | **GSE113052** | **0.309 (0.063, 0.555); p = 0.014** |
|  |  | **GSE125733** | **0.296 (0.047, 0.545); p = 0.020** |
|  |  | GSE179054 | *0.260 (-0.015, 0.535); p = 0.064* |
|  |  | GSE186075 | 0.214 (-0.087, 0.515); p = 0.164 |
|  |  | GSE58934 | *0.247 (-0.010, 0.505); p = 0.060* |
|  |  | GSE59131 | 0.225 (-0.040, 0.490); p = 0.097 |
| CCCA_vs_Control | **Senescence** | **Meta-analysis** | **-0.185 (-0.279, -0.092); p = 0.000** |
|  |  | GSE113052 | -0.187 (-0.415, 0.041); p = 0.109 |
|  |  | GSE125733 | *-0.207 (-0.419, 0.005); p = 0.056* |
|  |  | GSE179054 | -0.200 (-0.423, 0.023); p = 0.079 |
|  |  | GSE186075 | -0.100 (-0.350, 0.150); p = 0.434 |
|  |  | GSE58934 | -0.199 (-0.423, 0.025); p = 0.081 |
|  |  | GSE59131 | -0.201 (-0.445, 0.043); p = 0.107 |
| CCCA_vs_Control | **Skin_Immune_Cells** | Meta-analysis | 0.094 (-0.051, 0.239); p = 0.204 |
|  |  | GSE113052 | 0.106 (-0.238, 0.450); p = 0.546 |
|  |  | GSE125733 | 0.167 (-0.137, 0.471); p = 0.282 |
|  |  | GSE179054 | 0.105 (-0.281, 0.491); p = 0.594 |
|  |  | GSE186075 | 0.008 (-0.386, 0.403); p = 0.968 |
|  |  | GSE58934 | 0.093 (-0.252, 0.438); p = 0.596 |
|  |  | GSE59131 | 0.034 (-0.346, 0.415); p = 0.860 |
| CCCA_vs_Control | **T_cell_exhaustion** | **Meta-analysis** | **0.222 (0.097, 0.346); p = 0.000** |
|  |  | GSE113052 | 0.219 (-0.082, 0.521); p = 0.154 |
|  |  | **GSE125733** | **0.284 (0.023, 0.545); p = 0.033** |
|  |  | GSE179054 | 0.237 (-0.098, 0.572); p = 0.165 |
|  |  | GSE186075 | 0.200 (-0.156, 0.555); p = 0.271 |
|  |  | GSE58934 | 0.182 (-0.107, 0.471); p = 0.218 |
|  |  | GSE59131 | 0.184 (-0.127, 0.495); p = 0.246 |
| CCCA_vs_Control | **Th1** | **Meta-analysis** | **0.224 (0.052, 0.396); p = 0.011** |
|  |  | GSE113052 | 0.242 (-0.185, 0.669); p = 0.267 |
|  |  | GSE125733 | 0.311 (-0.052, 0.675); p = 0.093 |
|  |  | GSE179054 | 0.208 (-0.254, 0.670); p = 0.377 |
|  |  | GSE186075 | 0.169 (-0.321, 0.659); p = 0.499 |
|  |  | GSE58934 | 0.182 (-0.227, 0.591); p = 0.382 |
|  |  | GSE59131 | 0.189 (-0.225, 0.603); p = 0.371 |
| CCCA_vs_Control | **Th17** | Meta-analysis | 0.062 (-0.081, 0.205); p = 0.397 |
|  |  | GSE113052 | 0.050 (-0.313, 0.412); p = 0.788 |
|  |  | GSE125733 | 0.125 (-0.194, 0.443); p = 0.443 |
|  |  | GSE179054 | 0.065 (-0.298, 0.428); p = 0.726 |
|  |  | GSE186075 | 0.075 (-0.321, 0.471); p = 0.710 |
|  |  | GSE58934 | 0.041 (-0.297, 0.379); p = 0.812 |
|  |  | GSE59131 | 0.009 (-0.334, 0.352); p = 0.959 |
| CCCA_vs_Control | **Th2** | Meta-analysis | -0.098 (-0.238, 0.041); p = 0.168 |
|  |  | GSE113052 | -0.160 (-0.487, 0.166); p = 0.335 |
|  |  | GSE125733 | -0.086 (-0.423, 0.250); p = 0.615 |
|  |  | GSE179054 | -0.064 (-0.395, 0.268); p = 0.706 |
|  |  | GSE186075 | -0.036 (-0.400, 0.328); p = 0.847 |
|  |  | GSE58934 | -0.084 (-0.417, 0.248); p = 0.620 |
|  |  | GSE59131 | -0.156 (-0.524, 0.211); p = 0.404 |
| CCCA_vs_Control | **Th22** | **Meta-analysis** | **-0.309 (-0.486, -0.131); p = 0.001** |
|  |  | **GSE113052** | **-0.433 (-0.853, -0.014); p = 0.043** |
|  |  | GSE125733 | -0.278 (-0.699, 0.143); p = 0.196 |
|  |  | GSE179054 | -0.260 (-0.687, 0.168); p = 0.234 |
|  |  | GSE186075 | -0.197 (-0.659, 0.266); p = 0.404 |
|  |  | GSE58934 | -0.292 (-0.718, 0.133); p = 0.178 |
|  |  | GSE59131 | -0.381 (-0.838, 0.076); p = 0.102 |
| CCCA_vs_Control | **Th9** | Meta-analysis | *0.145 (-0.008, 0.297); p = 0.063* |
|  |  | GSE113052 | 0.174 (-0.180, 0.527); p = 0.337 |
|  |  | GSE125733 | 0.196 (-0.156, 0.547); p = 0.275 |
|  |  | GSE179054 | 0.166 (-0.223, 0.554); p = 0.403 |
|  |  | GSE186075 | 0.029 (-0.359, 0.416); p = 0.884 |
|  |  | GSE58934 | 0.136 (-0.232, 0.503); p = 0.470 |
|  |  | GSE59131 | 0.156 (-0.246, 0.559); p = 0.447 |
| CCCA_vs_Control | **Tregs** | **Meta-analysis** | **0.212 (0.086, 0.338); p = 0.001** |
|  |  | GSE113052 | 0.249 (-0.070, 0.567); p = 0.126 |
|  |  | **GSE125733** | **0.276 (0.011, 0.542); p = 0.041** |
|  |  | GSE179054 | 0.202 (-0.147, 0.551); p = 0.257 |
|  |  | GSE186075 | 0.163 (-0.192, 0.517); p = 0.369 |
|  |  | GSE58934 | 0.172 (-0.122, 0.467); p = 0.252 |
|  |  | GSE59131 | 0.180 (-0.117, 0.477); p = 0.236 |
| CCCA_vs_Control | **Type_I_IFN_Response** | **Meta-analysis** | **0.200 (0.032, 0.368); p = 0.020** |
|  |  | GSE113052 | 0.243 (-0.172, 0.659); p = 0.251 |
|  |  | GSE125733 | 0.266 (-0.102, 0.634); p = 0.156 |
|  |  | GSE179054 | 0.217 (-0.184, 0.619); p = 0.289 |
|  |  | GSE186075 | 0.040 (-0.415, 0.495); p = 0.863 |
|  |  | GSE58934 | 0.195 (-0.212, 0.601); p = 0.348 |
|  |  | GSE59131 | 0.192 (-0.247, 0.631); p = 0.392 |
| CCCA_vs_Control | **Wnt_Signaling** | Meta-analysis | -0.002 (-0.099, 0.096); p = 0.975 |
|  |  | GSE113052 | 0.014 (-0.226, 0.255); p = 0.907 |
|  |  | GSE125733 | -0.023 (-0.251, 0.204); p = 0.842 |
|  |  | GSE179054 | 0.018 (-0.232, 0.269); p = 0.887 |
|  |  | GSE186075 | -0.028 (-0.271, 0.214); p = 0.818 |
|  |  | GSE58934 | 0.004 (-0.230, 0.238); p = 0.972 |
|  |  | GSE59131 | 0.009 (-0.235, 0.253); p = 0.941 |
| CCCA_vs_Control | **gd_T_Cells** | Meta-analysis | 0.094 (-0.055, 0.244); p = 0.217 |
|  |  | GSE113052 | 0.126 (-0.245, 0.497); p = 0.505 |
|  |  | GSE125733 | 0.166 (-0.155, 0.487); p = 0.311 |
|  |  | GSE179054 | 0.105 (-0.303, 0.513); p = 0.614 |
|  |  | GSE186075 | 0.049 (-0.362, 0.459); p = 0.816 |
|  |  | GSE58934 | 0.061 (-0.294, 0.416); p = 0.736 |
|  |  | GSE59131 | 0.036 (-0.319, 0.392); p = 0.841 |
|  |  |  |  |
|  |  |  |  |
| FFA_vs_Control | **Apoptosis** | **Meta-analysis** | **0.169 (0.033, 0.305); p = 0.015** |
|  |  | GSE113052 | 0.205 (-0.099, 0.509); p = 0.185 |
|  |  | GSE125733 | *0.270 (-0.010, 0.550); p = 0.059* |
|  |  | GSE186075 | -0.044 (-0.388, 0.299); p = 0.801 |
|  |  | GSE58934 | 0.200 (-0.091, 0.491); p = 0.179 |
|  |  | GSE59131 | 0.144 (-0.172, 0.459); p = 0.371 |
| FFA_vs_Control | **B_Cells** | **Meta-analysis** | **0.469 (0.318, 0.620); p = 0.000** |
|  |  | **GSE113052** | **0.469 (0.135, 0.803); p = 0.006** |
|  |  | **GSE125733** | **0.572 (0.273, 0.871); p = 0.000** |
|  |  | GSE186075 | *0.375 (-0.014, 0.764); p = 0.059* |
|  |  | **GSE58934** | **0.455 (0.121, 0.789); p = 0.008** |
|  |  | **GSE59131** | **0.420 (0.071, 0.769); p = 0.018** |
| FFA_vs_Control | **Cellular_Stress** | Meta-analysis | -0.042 (-0.193, 0.109); p = 0.587 |
|  |  | GSE113052 | -0.044 (-0.367, 0.280); p = 0.791 |
|  |  | GSE125733 | -0.052 (-0.379, 0.276); p = 0.757 |
|  |  | GSE186075 | -0.035 (-0.428, 0.359); p = 0.863 |
|  |  | GSE58934 | -0.042 (-0.370, 0.286); p = 0.802 |
|  |  | GSE59131 | -0.035 (-0.363, 0.293); p = 0.836 |
| FFA_vs_Control | **Cornified_Envelope** | Meta-analysis | -0.005 (-0.212, 0.201); p = 0.961 |
|  |  | GSE113052 | -0.030 (-0.479, 0.419); p = 0.897 |
|  |  | GSE125733 | 0.073 (-0.381, 0.527); p = 0.753 |
|  |  | GSE186075 | -0.062 (-0.652, 0.529); p = 0.838 |
|  |  | GSE58934 | -0.003 (-0.413, 0.408); p = 0.990 |
|  |  | GSE59131 | -0.028 (-0.482, 0.426); p = 0.904 |
| FFA_vs_Control | **Cytotoxicity** | **Meta-analysis** | **0.667 (0.473, 0.861); p = 0.000** |
|  |  | **GSE113052** | **0.695 (0.270, 1.120); p = 0.001** |
|  |  | **GSE125733** | **0.800 (0.412, 1.189); p = 0.000** |
|  |  | GSE186075 | 0.453 (-0.074, 0.979); p = 0.092 |
|  |  | **GSE58934** | **0.649 (0.221, 1.077); p = 0.003** |
|  |  | **GSE59131** | **0.637 (0.203, 1.071); p = 0.004** |
| FFA_vs_Control | **DNA_Damage** | **Meta-analysis** | **0.177 (0.033, 0.321); p = 0.016** |
|  |  | GSE113052 | 0.160 (-0.158, 0.478); p = 0.323 |
|  |  | GSE125733 | 0.124 (-0.197, 0.444); p = 0.449 |
|  |  | GSE186075 | 0.290 (-0.038, 0.618); p = 0.083 |
|  |  | GSE58934 | 0.177 (-0.143, 0.496); p = 0.279 |
|  |  | GSE59131 | 0.139 (-0.188, 0.467); p = 0.404 |
| FFA_vs_Control | **Dendritic_Cells** | **Meta-analysis** | **0.493 (0.322, 0.663); p = 0.000** |
|  |  | **GSE113052** | **0.509 (0.131, 0.887); p = 0.008** |
|  |  | **GSE125733** | **0.637 (0.307, 0.968); p = 0.000** |
|  |  | GSE186075 | 0.261 (-0.182, 0.704); p = 0.248 |
|  |  | **GSE58934** | **0.488 (0.106, 0.869); p = 0.012** |
|  |  | **GSE59131** | **0.458 (0.061, 0.855); p = 0.024** |
| FFA_vs_Control | **EMT** | **Meta-analysis** | **0.223 (0.075, 0.372); p = 0.003** |
|  |  | GSE113052 | 0.207 (-0.115, 0.530); p = 0.207 |
|  |  | **GSE125733** | **0.315 (0.016, 0.615); p = 0.039** |
|  |  | GSE186075 | 0.077 (-0.331, 0.484); p = 0.712 |
|  |  | GSE58934 | 0.240 (-0.076, 0.556); p = 0.136 |
|  |  | GSE59131 | 0.204 (-0.136, 0.545); p = 0.240 |
| FFA_vs_Control | **Epidermal_Keratins** | Meta-analysis | -0.117 (-0.289, 0.054); p = 0.181 |
|  |  | GSE113052 | -0.147 (-0.523, 0.228); p = 0.443 |
|  |  | GSE125733 | -0.089 (-0.466, 0.289); p = 0.645 |
|  |  | GSE186075 | -0.125 (-0.612, 0.362); p = 0.616 |
|  |  | GSE58934 | -0.134 (-0.471, 0.203); p = 0.435 |
|  |  | GSE59131 | -0.089 (-0.472, 0.294); p = 0.649 |
| FFA_vs_Control | **Fibroblasts** | **Meta-analysis** | **0.354 (0.192, 0.516); p = 0.000** |
|  |  | **GSE113052** | **0.370 (0.007, 0.733); p = 0.046** |
|  |  | **GSE125733** | **0.506 (0.206, 0.807); p = 0.001** |
|  |  | GSE186075 | 0.003 (-0.428, 0.434); p = 0.989 |
|  |  | GSE58934 | *0.363 (-0.005, 0.732); p = 0.053* |
|  |  | GSE59131 | *0.357 (-0.029, 0.742); p = 0.070* |
| FFA_vs_Control | **Fibrosis_TGFB_Signaling** | **Meta-analysis** | **0.230 (0.075, 0.385); p = 0.004** |
|  |  | GSE113052 | 0.246 (-0.096, 0.589); p = 0.158 |
|  |  | **GSE125733** | **0.358 (0.057, 0.659); p = 0.020** |
|  |  | GSE186075 | -0.047 (-0.451, 0.357); p = 0.819 |
|  |  | GSE58934 | 0.240 (-0.109, 0.590); p = 0.178 |
|  |  | GSE59131 | 0.237 (-0.123, 0.598); p = 0.197 |
| FFA_vs_Control | **Follicle_Structure** | Meta-analysis | -0.118 (-0.327, 0.091); p = 0.269 |
|  |  | GSE113052 | -0.184 (-0.632, 0.264); p = 0.421 |
|  |  | GSE125733 | -0.220 (-0.682, 0.242); p = 0.351 |
|  |  | GSE186075 | 0.169 (-0.356, 0.695); p = 0.527 |
|  |  | GSE58934 | -0.141 (-0.580, 0.298); p = 0.530 |
|  |  | GSE59131 | -0.144 (-0.617, 0.329); p = 0.552 |
| FFA_vs_Control | **Follicular_Keratins** | Meta-analysis | -0.022 (-0.290, 0.246); p = 0.871 |
|  |  | GSE113052 | -0.081 (-0.646, 0.484); p = 0.779 |
|  |  | GSE125733 | -0.114 (-0.689, 0.461); p = 0.697 |
|  |  | GSE186075 | 0.288 (-0.479, 1.055); p = 0.462 |
|  |  | GSE58934 | -0.022 (-0.585, 0.540); p = 0.938 |
|  |  | GSE59131 | -0.044 (-0.631, 0.543); p = 0.883 |
| FFA_vs_Control | **HF_Immune_Privilege** | **Meta-analysis** | **0.434 (0.275, 0.593); p = 0.000** |
|  |  | GSE113052 | 0.448 (0.099, 0.797); p = 0.012 |
|  |  | **GSE125733** | **0.547 (0.251, 0.843); p = 0.000** |
|  |  | GSE186075 | 0.272 (-0.177, 0.722); p = 0.235 |
|  |  | **GSE58934** | **0.408 (0.054, 0.763); p = 0.024** |
|  |  | **GSE59131** | **0.379 (0.001, 0.757); p = 0.049** |
| FFA_vs_Control | **JAK_STAT_Pathway** | **Meta-analysis** | **0.343 (0.170, 0.516); p = 0.000** |
|  |  | GSE113052 | *0.321 (-0.053, 0.696); p = 0.093* |
|  |  | **GSE125733** | **0.478 (0.144, 0.813); p = 0.005** |
|  |  | GSE186075 | 0.103 (-0.389, 0.596); p = 0.681 |
|  |  | GSE58934 | *0.352 (-0.028, 0.732); p = 0.070* |
|  |  | GSE59131 | 0.323 (-0.075, 0.721); p = 0.112 |
| FFA_vs_Control | **Keratinocytes** | Meta-analysis | -0.060 (-0.201, 0.080); p = 0.399 |
|  |  | GSE113052 | -0.092 (-0.399, 0.215); p = 0.556 |
|  |  | GSE125733 | -0.057 (-0.369, 0.256); p = 0.722 |
|  |  | GSE186075 | 0.090 (-0.321, 0.500); p = 0.669 |
|  |  | GSE58934 | -0.077 (-0.347, 0.192); p = 0.575 |
|  |  | GSE59131 | -0.095 (-0.408, 0.217); p = 0.551 |
| FFA_vs_Control | **MAIT_Cells** | **Meta-analysis** | **0.494 (0.335, 0.653); p = 0.000** |
|  |  | **GSE113052** | **0.510 (0.163, 0.857); p = 0.004** |
|  |  | **GSE125733** | **0.597 (0.281, 0.912); p = 0.000** |
|  |  | GSE186075 | 0.342 (-0.082, 0.766); p = 0.114 |
|  |  | **GSE58934** | **0.489 (0.142, 0.836); p = 0.006** |
|  |  | **GSE59131** | **0.457 (0.094, 0.821); p = 0.014** |
| FFA_vs_Control | **Macrophages** | **Meta-analysis** | **0.413 (0.276, 0.550); p = 0.000** |
|  |  | **GSE113052** | **0.422 (0.118, 0.727); p = 0.007** |
|  |  | **GSE125733** | **0.516 (0.240, 0.792); p = 0.000** |
|  |  | GSE186075 | 0.276 (-0.062, 0.614); p = 0.110 |
|  |  | **GSE58934** | **0.406 (0.102, 0.710); p = 0.009** |
|  |  | **GSE59131** | **0.393 (0.074, 0.711); p = 0.016** |
| FFA_vs_Control | **Mast_Cells** | **Meta-analysis** | **0.423 (0.276, 0.570); p = 0.000** |
|  |  | **GSE113052** | **0.469 (0.144, 0.794); p = 0.005** |
|  |  | **GSE125733** | **0.550 (0.251, 0.848); p = 0.000** |
|  |  | GSE186075 | 0.138 (-0.210, 0.486); p = 0.436 |
|  |  | **GSE58934** | **0.456 (0.122, 0.790); p = 0.007** |
|  |  | **GSE59131** | **0.449 (0.103, 0.795); p = 0.011** |
| FFA_vs_Control | **Memory_T_Cells** | **Meta-analysis** | **0.502 (0.353, 0.650); p = 0.000** |
|  |  | **GSE113052** | **0.519 (0.188, 0.851); p = 0.002** |
|  |  | **GSE125733** | **0.593 (0.291, 0.895); p = 0.000** |
|  |  | **GSE186075** | **0.392 (0.032, 0.752); p = 0.033** |
|  |  | **GSE58934** | **0.497 (0.171, 0.823); p = 0.003** |
|  |  | **GSE59131** | **0.469 (0.120, 0.817); p = 0.008** |
| FFA_vs_Control | **Monocytes** | **Meta-analysis** | **0.485 (0.328, 0.643); p = 0.000** |
|  |  | **GSE113052** | **0.511 (0.167, 0.855); p = 0.004** |
|  |  | **GSE125733** | **0.601 (0.287, 0.915); p = 0.000** |
|  |  | GSE186075 | 0.344 (-0.074, 0.761); p = 0.107 |
|  |  | **GSE58934** | **0.473 (0.126, 0.819); p = 0.007** |
|  |  | **GSE59131** | **0.423 (0.057, 0.789); p = 0.024** |
| FFA_vs_Control | **NK_Cells** | **Meta-analysis** | **0.559 (0.399, 0.719); p = 0.000** |
|  |  | **GSE113052** | **0.604 (0.260, 0.949); p = 0.001** |
|  |  | **GSE125733** | **0.660 (0.335, 0.986); p = 0.000** |
|  |  | GSE186075 | 0.315 (-0.113, 0.742); p = 0.149 |
|  |  | **GSE58934** | **0.564 (0.212, 0.916); p = 0.002** |
|  |  | **GSE59131** | **0.555 (0.194, 0.916); p = 0.003** |
| FFA_vs_Control | **Neutrophils** | **Meta-analysis** | **0.364 (0.239, 0.488); p = 0.000** |
|  |  | **GSE113052** | **0.390 (0.126, 0.654); p = 0.004** |
|  |  | **GSE125733** | **0.427 (0.170, 0.683); p = 0.001** |
|  |  | GSE186075 | 0.304 (-0.053, 0.661); p = 0.095 |
|  |  | **GSE58934** | **0.340 (0.070, 0.610); p = 0.014** |
|  |  | **GSE59131** | **0.325 (0.054, 0.595); p = 0.019** |
| FFA_vs_Control | **Senescence** | Meta-analysis | *-0.116 (-0.237, 0.005); p = 0.060* |
|  |  | GSE113052 | -0.114 (-0.376, 0.147); p = 0.392 |
|  |  | GSE125733 | -0.167 (-0.417, 0.083); p = 0.191 |
|  |  | GSE186075 | -0.041 (-0.338, 0.255); p = 0.784 |
|  |  | GSE58934 | -0.121 (-0.387, 0.145); p = 0.372 |
|  |  | GSE59131 | -0.115 (-0.399, 0.168); p = 0.425 |
| FFA_vs_Control | **Skin_Immune_Cells** | **Meta-analysis** | **0.535 (0.350, 0.721); p = 0.000** |
|  |  | **GSE113052** | **0.546 (0.143, 0.948); p = 0.008** |
|  |  | **GSE125733** | **0.668 (0.304, 1.033); p = 0.000** |
|  |  | GSE186075 | 0.370 (-0.139, 0.879); p = 0.154 |
|  |  | **GSE58934** | **0.524 (0.118, 0.930); p = 0.012** |
|  |  | **GSE59131** | **0.470 (0.041, 0.898); p = 0.032** |
| FFA_vs_Control | **T_cell_exhaustion** | **Meta-analysis** | **0.425 (0.255, 0.595); p = 0.000** |
|  |  | **GSE113052** | **0.452 (0.074, 0.830); p = 0.019** |
|  |  | **GSE125733** | **0.528 (0.180, 0.875); p = 0.003** |
|  |  | GSE186075 | 0.187 (-0.268, 0.643); p = 0.421 |
|  |  | **GSE58934** | **0.449 (0.096, 0.802); p = 0.013** |
|  |  | **GSE59131** | **0.410 (0.019, 0.801); p = 0.040** |
| FFA_vs_Control | **Th1** | **Meta-analysis** | **0.629 (0.425, 0.833); p = 0.000** |
|  |  | **GSE113052** | **0.645 (0.195, 1.094); p = 0.005** |
|  |  | **GSE125733** | **0.763 (0.359, 1.167); p = 0.000** |
|  |  | GSE186075 | 0.345 (-0.254, 0.944); p = 0.259 |
|  |  | **GSE58934** | **0.616 (0.180, 1.052); p = 0.006** |
|  |  | **GSE59131** | **0.620 (0.170, 1.071); p = 0.007** |
| FFA_vs_Control | **Th17** | Meta-analysis | *0.152 (-0.004, 0.308); p = 0.057* |
|  |  | GSE113052 | 0.163 (-0.179, 0.506); p = 0.350 |
|  |  | GSE125733 | 0.254 (-0.053, 0.560); p = 0.105 |
|  |  | GSE186075 | 0.045 (-0.444, 0.533); p = 0.858 |
|  |  | GSE58934 | 0.145 (-0.191, 0.480); p = 0.398 |
|  |  | GSE59131 | 0.076 (-0.261, 0.412); p = 0.659 |
| FFA_vs_Control | **Th2** | Meta-analysis | 0.071 (-0.065, 0.207); p = 0.307 |
|  |  | GSE113052 | 0.033 (-0.255, 0.321); p = 0.822 |
|  |  | GSE125733 | 0.098 (-0.188, 0.383); p = 0.502 |
|  |  | GSE186075 | 0.170 (-0.203, 0.543); p = 0.371 |
|  |  | GSE58934 | 0.058 (-0.229, 0.345); p = 0.692 |
|  |  | GSE59131 | 0.029 (-0.285, 0.342); p = 0.856 |
| FFA_vs_Control | **Th22** | Meta-analysis | *-0.155 (-0.318, 0.009); p = 0.064* |
|  |  | GSE113052 | -0.207 (-0.546, 0.133); p = 0.233 |
|  |  | GSE125733 | -0.099 (-0.442, 0.244); p = 0.570 |
|  |  | GSE186075 | 0.053 (-0.442, 0.548); p = 0.834 |
|  |  | GSE58934 | -0.169 (-0.512, 0.174); p = 0.333 |
|  |  | GSE59131 | -0.251 (-0.613, 0.110); p = 0.173 |
| FFA_vs_Control | **Th9** | Meta-analysis | *0.162 (-0.017, 0.341); p = 0.076* |
|  |  | GSE113052 | 0.200 (-0.173, 0.573); p = 0.293 |
|  |  | GSE125733 | 0.265 (-0.103, 0.632); p = 0.158 |
|  |  | GSE186075 | -0.102 (-0.577, 0.374); p = 0.675 |
|  |  | GSE58934 | 0.168 (-0.228, 0.565); p = 0.406 |
|  |  | GSE59131 | 0.177 (-0.235, 0.589); p = 0.401 |
| FFA_vs_Control | **Tregs** | **Meta-analysis** | **0.418 (0.248, 0.588); p = 0.000** |
|  |  | **GSE113052** | **0.453 (0.078, 0.829); p = 0.018** |
|  |  | **GSE125733** | **0.528 (0.185, 0.871); p = 0.003** |
|  |  | GSE186075 | 0.175 (-0.287, 0.636); p = 0.459 |
|  |  | **GSE58934** | **0.416 (0.054, 0.778); p = 0.024** |
|  |  | **GSE59131** | **0.415 (0.030, 0.799); p = 0.035** |
| FFA_vs_Control | **Type_I_IFN_Response** | **Meta-analysis** | **0.550 (0.353, 0.748); p = 0.000** |
|  |  | **GSE113052** | **0.588 (0.155, 1.022); p = 0.008** |
|  |  | **GSE125733** | **0.692 (0.294, 1.090); p = 0.001** |
|  |  | GSE186075 | 0.326 (-0.168, 0.821); p = 0.196 |
|  |  | **GSE58934** | **0.541 (0.101, 0.982); p = 0.016** |
|  |  | **GSE59131** | **0.523 (0.062, 0.984); p = 0.026** |
| FFA_vs_Control | **Wnt_Signaling** | Meta-analysis | 0.001 (-0.116, 0.118); p = 0.985 |
|  |  | GSE113052 | 0.005 (-0.249, 0.258); p = 0.972 |
|  |  | GSE125733 | -0.041 (-0.290, 0.209); p = 0.750 |
|  |  | GSE186075 | 0.069 (-0.219, 0.356); p = 0.638 |
|  |  | GSE58934 | -0.009 (-0.269, 0.250); p = 0.943 |
|  |  | GSE59131 | -0.002 (-0.266, 0.263); p = 0.989 |
| FFA_vs_Control | **gd_T_Cells** | **Meta-analysis** | **0.511 (0.323, 0.698); p = 0.000** |
|  |  | **GSE113052** | **0.541 (0.135, 0.946); p = 0.009** |
|  |  | **GSE125733** | **0.618 (0.240, 0.997); p = 0.001** |
|  |  | GSE186075 | 0.331 (-0.220, 0.882); p = 0.239 |
|  |  | **GSE58934** | **0.502 (0.098, 0.906); p = 0.015** |
|  |  | **GSE59131** | **0.463 (0.054, 0.872); p = 0.027** |
|  |  |  |  |
|  |  |  |  |
| LPP_vs_Control | **Apoptosis** | Meta-analysis | 0.086 (-0.040, 0.211); p = 0.183 |
|  |  | GSE113052 | 0.080 (-0.203, 0.362); p = 0.581 |
|  |  | GSE125733 | 0.113 (-0.146, 0.371); p = 0.394 |
|  |  | GSE186075 | 0.373 (0.015, 0.731); p = 0.041 |
|  |  | GSE58934 | 0.049 (-0.220, 0.318); p = 0.723 |
|  |  | GSE59131 | -0.061 (-0.328, 0.206); p = 0.653 |
| LPP_vs_Control | **B_Cells** | **Meta-analysis** | **0.351 (0.189, 0.512); p = 0.000** |
|  |  | GSE113052 | *0.326 (-0.031, 0.683); p = 0.073* |
|  |  | **GSE125733** | **0.395 (0.074, 0.716); p = 0.016** |
|  |  | **GSE186075** | **0.518 (0.088, 0.948); p = 0.018** |
|  |  | GSE58934 | *0.322 (-0.027, 0.672); p = 0.071* |
|  |  | GSE59131 | 0.226 (-0.143, 0.596); p = 0.230 |
| LPP_vs_Control | **Cellular_Stress** | Meta-analysis | -0.065 (-0.224, 0.093); p = 0.419 |
|  |  | GSE113052 | -0.086 (-0.434, 0.262); p = 0.628 |
|  |  | GSE125733 | -0.100 (-0.440, 0.240); p = 0.566 |
|  |  | GSE186075 | 0.220 (-0.227, 0.668); p = 0.334 |
|  |  | GSE58934 | -0.071 (-0.419, 0.276); p = 0.687 |
|  |  | GSE59131 | -0.162 (-0.488, 0.163); p = 0.328 |
| LPP_vs_Control | **Cornified_Envelope** | Meta-analysis | *0.218 (-0.010, 0.445); p = 0.061* |
|  |  | GSE113052 | 0.180 (-0.316, 0.677); p = 0.477 |
|  |  | GSE125733 | 0.229 (-0.257, 0.716); p = 0.355 |
|  |  | GSE186075 | 0.288 (-0.312, 0.888); p = 0.347 |
|  |  | GSE58934 | 0.264 (-0.208, 0.736); p = 0.273 |
|  |  | GSE59131 | 0.138 (-0.375, 0.651); p = 0.598 |
| LPP_vs_Control | **Cytotoxicity** | **Meta-analysis** | **0.431 (0.200, 0.662); p = 0.000** |
|  |  | GSE113052 | 0.419 (-0.091, 0.929); p = 0.107 |
|  |  | **GSE125733** | **0.488 (0.024, 0.951); p = 0.039** |
|  |  | **GSE186075** | **0.647 (0.033, 1.261); p = 0.039** |
|  |  | GSE58934 | 0.370 (-0.129, 0.869); p = 0.146 |
|  |  | GSE59131 | 0.279 (-0.250, 0.809); p = 0.301 |
| LPP_vs_Control | **DNA_Damage** | Meta-analysis | 0.106 (-0.039, 0.252); p = 0.153 |
|  |  | GSE113052 | 0.106 (-0.214, 0.425); p = 0.517 |
|  |  | GSE125733 | 0.099 (-0.212, 0.411); p = 0.532 |
|  |  | GSE186075 | 0.121 (-0.220, 0.462); p = 0.486 |
|  |  | GSE58934 | 0.104 (-0.214, 0.421); p = 0.522 |
|  |  | GSE59131 | 0.104 (-0.241, 0.448); p = 0.556 |
| LPP_vs_Control | **Dendritic_Cells** | **Meta-analysis** | **0.335 (0.154, 0.516); p = 0.000** |
|  |  | GSE113052 | 0.320 (-0.078, 0.719); p = 0.115 |
|  |  | **GSE125733** | **0.390 (0.036, 0.744); p = 0.031** |
|  |  | GSE186075 | *0.442 (-0.044, 0.928); p = 0.075* |
|  |  | GSE58934 | 0.308 (-0.084, 0.699); p = 0.123 |
|  |  | GSE59131 | 0.224 (-0.200, 0.649); p = 0.301 |
| LPP_vs_Control | **EMT** | **Meta-analysis** | **0.199 (0.038, 0.361); p = 0.016** |
|  |  | GSE113052 | 0.159 (-0.193, 0.512); p = 0.375 |
|  |  | GSE125733 | 0.214 (-0.114, 0.542); p = 0.201 |
|  |  | GSE186075 | 0.320 (-0.107, 0.748); p = 0.141 |
|  |  | GSE58934 | 0.216 (-0.127, 0.558); p = 0.217 |
|  |  | GSE59131 | 0.110 (-0.272, 0.491); p = 0.573 |
| LPP_vs_Control | **Epidermal_Keratins** | Meta-analysis | -0.084 (-0.247, 0.079); p = 0.312 |
|  |  | GSE113052 | -0.106 (-0.461, 0.249); p = 0.559 |
|  |  | GSE125733 | -0.075 (-0.421, 0.271); p = 0.672 |
|  |  | GSE186075 | -0.174 (-0.600, 0.252); p = 0.424 |
|  |  | GSE58934 | -0.040 (-0.376, 0.295); p = 0.815 |
|  |  | GSE59131 | -0.055 (-0.434, 0.323); p = 0.775 |
| LPP_vs_Control | **Fibroblasts** | **Meta-analysis** | **0.468 (0.297, 0.639); p = 0.000** |
|  |  | **GSE113052** | **0.453 (0.078, 0.828); p = 0.018** |
|  |  | **GSE125733** | **0.510 (0.184, 0.836); p = 0.002** |
|  |  | **GSE186075** | **0.522 (0.036, 1.008); p = 0.035** |
|  |  | **GSE58934** | **0.456 (0.086, 0.826); p = 0.016** |
|  |  | *GSE59131* | *0.400 (-0.002, 0.802); p = 0.051* |
| LPP_vs_Control | **Fibrosis_TGFB_Signaling** | **Meta-analysis** | **0.343 (0.173, 0.514); p = 0.000** |
|  |  | GSE113052 | 0.320 (-0.054, 0.693); p = 0.093 |
|  |  | **GSE125733** | **0.367 (0.031, 0.703); p = 0.032** |
|  |  | **GSE186075** | **0.495 (0.036, 0.954); p = 0.034** |
|  |  | GSE58934 | 0.320 (-0.051, 0.690); p = 0.091 |
|  |  | GSE59131 | 0.249 (-0.148, 0.647); p = 0.219 |
| LPP_vs_Control | **Follicle_Structure** | Meta-analysis | -0.124 (-0.342, 0.093); p = 0.263 |
|  |  | GSE113052 | -0.122 (-0.588, 0.345); p = 0.610 |
|  |  | GSE125733 | -0.132 (-0.594, 0.330); p = 0.575 |
|  |  | GSE186075 | -0.432 (-0.999, 0.135); p = 0.135 |
|  |  | GSE58934 | -0.035 (-0.496, 0.425); p = 0.880 |
|  |  | GSE59131 | 0.016 (-0.482, 0.514); p = 0.950 |
| LPP_vs_Control | **Follicular_Keratins** | Meta-analysis | 0.022 (-0.261, 0.305); p = 0.878 |
|  |  | GSE113052 | 0.012 (-0.586, 0.609); p = 0.969 |
|  |  | GSE125733 | 0.008 (-0.586, 0.602); p = 0.979 |
|  |  | GSE186075 | -0.333 (-1.155, 0.490); p = 0.428 |
|  |  | GSE58934 | 0.118 (-0.476, 0.712); p = 0.697 |
|  |  | GSE59131 | 0.150 (-0.479, 0.779); p = 0.640 |
| LPP_vs_Control | **HF_Immune_Privilege** | **Meta-analysis** | **0.240 (0.081, 0.400); p = 0.003** |
|  |  | GSE113052 | 0.237 (-0.110, 0.585); p = 0.181 |
|  |  | GSE125733 | *0.298 (-0.004, 0.600); p = 0.053* |
|  |  | GSE186075 | 0.320 (-0.122, 0.762); p = 0.155 |
|  |  | GSE58934 | 0.210 (-0.136, 0.556); p = 0.234 |
|  |  | GSE59131 | 0.127 (-0.261, 0.514); p = 0.521 |
| LPP_vs_Control | **JAK_STAT_Pathway** | **Meta-analysis** | **0.235 (0.045, 0.426); p = 0.015** |
|  |  | GSE113052 | 0.169 (-0.245, 0.584); p = 0.423 |
|  |  | GSE125733 | 0.272 (-0.114, 0.659); p = 0.167 |
|  |  | **GSE186075** | **0.554 (0.045, 1.062); p = 0.033** |
|  |  | GSE58934 | 0.193 (-0.222, 0.608); p = 0.362 |
|  |  | GSE59131 | 0.078 (-0.352, 0.509); p = 0.722 |
| LPP_vs_Control | **Keratinocytes** | Meta-analysis | 0.020 (-0.128, 0.168); p = 0.792 |
|  |  | GSE113052 | -0.009 (-0.331, 0.313); p = 0.957 |
|  |  | GSE125733 | 0.018 (-0.299, 0.335); p = 0.912 |
|  |  | GSE186075 | 0.098 (-0.311, 0.508); p = 0.637 |
|  |  | GSE58934 | 0.054 (-0.252, 0.360); p = 0.729 |
|  |  | GSE59131 | -0.037 (-0.365, 0.290); p = 0.823 |
| LPP_vs_Control | **MAIT_Cells** | **Meta-analysis** | **0.318 (0.141, 0.495); p = 0.000** |
|  |  | GSE113052 | 0.303 (-0.087, 0.692); p = 0.128 |
|  |  | **GSE125733** | **0.365 (0.014, 0.717); p = 0.041** |
|  |  | **GSE186075** | **0.531 (0.039, 1.023); p = 0.034** |
|  |  | GSE58934 | 0.273 (-0.109, 0.655); p = 0.162 |
|  |  | GSE59131 | 0.181 (-0.220, 0.581); p = 0.376 |
| LPP_vs_Control | **Macrophages** | **Meta-analysis** | **0.306 (0.158, 0.454); p = 0.000** |
|  |  | GSE113052 | 0.285 (-0.043, 0.614); p = 0.089 |
|  |  | GSE125733 | **0.345 (0.049, 0.642); p = 0.023** |
|  |  | GSE186075 | **0.412 (0.042, 0.781); p = 0.029** |
|  |  | GSE58934 | 0.271 (-0.049, 0.590); p = 0.097 |
|  |  | GSE59131 | 0.218 (-0.138, 0.574); p = 0.229 |
| LPP_vs_Control | **Mast_Cells** | **Meta-analysis** | **0.421 (0.270, 0.571); p = 0.000** |
|  |  | **GSE113052** | **0.439 (0.114, 0.765); p = 0.008** |
|  |  | **GSE125733** | **0.469 (0.171, 0.766); p = 0.002** |
|  |  | GSE186075 | 0.302 (-0.087, 0.690); p = 0.128 |
|  |  | **GSE58934** | **0.424 (0.097, 0.751); p = 0.011** |
|  |  | **GSE59131** | **0.426 (0.059, 0.792); p = 0.023** |
| LPP_vs_Control | **Memory_T_Cells** | **Meta-analysis** | **0.342 (0.189, 0.496); p = 0.000** |
|  |  | GSE113052 | *0.327 (-0.015, 0.669); p = 0.061* |
|  |  | **GSE125733** | **0.375 (0.070, 0.680); p = 0.016** |
|  |  | **GSE186075** | **0.496 (0.107, 0.885); p = 0.013** |
|  |  | GSE58934 | *0.306 (-0.027, 0.638); p = 0.071* |
|  |  | GSE59131 | 0.226 (-0.135, 0.587); p = 0.221 |
| LPP_vs_Control | **Monocytes** | **Meta-analysis** | **0.385 (0.224, 0.546); p = 0.000** |
|  |  | **GSE113052** | **0.393 (0.045, 0.742); p = 0.027** |
|  |  | **GSE125733** | **0.435 (0.117, 0.754); p = 0.007** |
|  |  | **GSE186075** | **0.496 (0.021, 0.971); p = 0.041** |
|  |  | **GSE58934** | **0.374 (0.028, 0.721); p = 0.034** |
|  |  | GSE59131 | 0.262 (-0.097, 0.622); p = 0.153 |
| LPP_vs_Control | **NK_Cells** | **Meta-analysis** | **0.441 (0.267, 0.614); p = 0.000** |
|  |  | **GSE113052** | **0.457 (0.084, 0.831); p = 0.016** |
|  |  | **GSE125733** | **0.480 (0.130, 0.830); p = 0.007** |
|  |  | **GSE186075** | **0.487 (0.024, 0.951); p = 0.039** |
|  |  | **GSE58934** | **0.404 (0.030, 0.779); p = 0.034** |
|  |  | *GSE59131* | *0.373 (-0.038, 0.783); p = 0.075* |
| LPP_vs_Control | **Neutrophils** | **Meta-analysis** | **0.384 (0.262, 0.506); p = 0.000** |
|  |  | **GSE113052** | **0.403 (0.144, 0.661); p = 0.002** |
|  |  | **GSE125733** | **0.414 (0.160, 0.667); p = 0.001** |
|  |  | **GSE186075** | **0.475 (0.101, 0.849); p = 0.013** |
|  |  | **GSE58934** | **0.365 (0.104, 0.626); p = 0.006** |
|  |  | **GSE59131** | **0.309 (0.048, 0.570); p = 0.020** |
| LPP_vs_Control | **Senescence** | Meta-analysis | -0.075 (-0.196, 0.046); p = 0.224 |
|  |  | GSE113052 | -0.074 (-0.334, 0.187); p = 0.580 |
|  |  | GSE125733 | -0.102 (-0.352, 0.149); p = 0.427 |
|  |  | GSE186075 | -0.006 (-0.305, 0.292); p = 0.967 |
|  |  | GSE58934 | -0.094 (-0.354, 0.167); p = 0.480 |
|  |  | GSE59131 | -0.083 (-0.376, 0.209); p = 0.577 |
| LPP_vs_Control | **Skin_Immune_Cells** | **Meta-analysis** | **0.290 (0.093, 0.487); p = 0.004** |
|  |  | GSE113052 | 0.275 (-0.152, 0.702); p = 0.207 |
|  |  | GSE125733 | *0.345 (-0.045, 0.736); p = 0.083* |
|  |  | GSE186075 | 0.425 (-0.105, 0.955); p = 0.116 |
|  |  | GSE58934 | 0.272 (-0.150, 0.694); p = 0.207 |
|  |  | GSE59131 | 0.147 (-0.320, 0.615); p = 0.537 |
| LPP_vs_Control | **T_cell_exhaustion** | **Meta-analysis** | **0.272 (0.089, 0.455); p = 0.004** |
|  |  | GSE113052 | 0.261 (-0.146, 0.668); p = 0.210 |
|  |  | GSE125733 | 0.319 (-0.050, 0.688); p = 0.090 |
|  |  | GSE186075 | 0.409 (-0.065, 0.883); p = 0.091 |
|  |  | GSE58934 | 0.217 (-0.172, 0.605); p = 0.275 |
|  |  | GSE59131 | 0.175 (-0.257, 0.607); p = 0.428 |
| LPP_vs_Control | **Th1** | **Meta-analysis** | **0.425 (0.198, 0.652); p = 0.000** |
|  |  | GSE113052 | 0.420 (-0.080, 0.920); p = 0.100 |
|  |  | **GSE125733** | **0.502 (0.055, 0.948); p = 0.028** |
|  |  | GSE186075 | 0.463 (-0.205, 1.131); p = 0.174 |
|  |  | GSE58934 | 0.373 (-0.111, 0.856); p = 0.131 |
|  |  | GSE59131 | 0.365 (-0.147, 0.877); p = 0.162 |
| LPP_vs_Control | **Th17** | Meta-analysis | 0.078 (-0.095, 0.251); p = 0.378 |
|  |  | GSE113052 | 0.060 (-0.324, 0.444); p = 0.760 |
|  |  | GSE125733 | 0.119 (-0.235, 0.472); p = 0.510 |
|  |  | GSE186075 | 0.409 (-0.072, 0.890); p = 0.096 |
|  |  | GSE58934 | 0.035 (-0.336, 0.406); p = 0.852 |
|  |  | GSE59131 | -0.110 (-0.488, 0.267); p = 0.566 |
| LPP_vs_Control | **Th2** | Meta-analysis | -0.094 (-0.233, 0.045); p = 0.186 |
|  |  | GSE113052 | -0.112 (-0.408, 0.183); p = 0.456 |
|  |  | GSE125733 | -0.063 (-0.364, 0.239); p = 0.684 |
|  |  | GSE186075 | -0.170 (-0.517, 0.177); p = 0.337 |
|  |  | GSE58934 | -0.060 (-0.358, 0.237); p = 0.690 |
|  |  | GSE59131 | -0.081 (-0.406, 0.243); p = 0.624 |
| LPP_vs_Control | **Th22** | **Meta-analysis** | **-0.359 (-0.525, -0.193); p = 0.000** |
|  |  | **GSE113052** | **-0.436 (-0.783, -0.090); p = 0.014** |
|  |  | *GSE125733* | *-0.347 (-0.700, 0.007); p = 0.054* |
|  |  | GSE186075 | -0.020 (-0.451, 0.411); p = 0.926 |
|  |  | **GSE58934** | **-0.361 (-0.719, -0.003); p = 0.048** |
|  |  | **GSE59131** | **-0.545 (-0.929, -0.161); p = 0.005** |
| LPP_vs_Control | **Th9** | Meta-analysis | 0.030 (-0.153, 0.214); p = 0.746 |
|  |  | GSE113052 | 0.054 (-0.324, 0.433); p = 0.778 |
|  |  | GSE125733 | 0.078 (-0.303, 0.459); p = 0.688 |
|  |  | GSE186075 | -0.078 (-0.575, 0.418); p = 0.757 |
|  |  | GSE58934 | 0.018 (-0.378, 0.413); p = 0.929 |
|  |  | GSE59131 | 0.035 (-0.401, 0.471); p = 0.875 |
| LPP_vs_Control | **Tregs** | **Meta-analysis** | **0.200 (0.024, 0.376); p = 0.026** |
|  |  | GSE113052 | 0.213 (-0.174, 0.600); p = 0.280 |
|  |  | GSE125733 | 0.259 (-0.090, 0.607); p = 0.146 |
|  |  | GSE186075 | 0.195 (-0.296, 0.685); p = 0.437 |
|  |  | GSE58934 | 0.155 (-0.216, 0.526); p = 0.414 |
|  |  | GSE59131 | 0.161 (-0.248, 0.571); p = 0.440 |
| LPP_vs_Control | **Type_I_IFN_Response** | **Meta-analysis** | **0.406 (0.208, 0.605); p = 0.000** |
|  |  | *GSE113052* | *0.422 (-0.007, 0.851); p = 0.054* |
|  |  | **GSE125733** | **0.466 (0.074, 0.857); p = 0.020** |
|  |  | GSE186075 | 0.380 (-0.207, 0.967); p = 0.204 |
|  |  | *GSE58934* | *0.394 (-0.033, 0.821); p = 0.071* |
|  |  | GSE59131 | 0.341 (-0.104, 0.786); p = 0.133 |
| LPP_vs_Control | **Wnt_Signaling** | Meta-analysis | 0.014 (-0.104, 0.132); p = 0.811 |
|  |  | GSE113052 | 0.026 (-0.229, 0.282); p = 0.841 |
|  |  | GSE125733 | -0.008 (-0.257, 0.242); p = 0.952 |
|  |  | GSE186075 | 0.018 (-0.262, 0.298); p = 0.902 |
|  |  | GSE58934 | 0.020 (-0.235, 0.275); p = 0.879 |
|  |  | GSE59131 | 0.018 (-0.267, 0.303); p = 0.900 |
| LPP_vs_Control | **gd_T_Cells** | **Meta-analysis** | **0.245 (0.030, 0.460); p = 0.026** |
|  |  | GSE113052 | 0.242 (-0.228, 0.711); p = 0.313 |
|  |  | GSE125733 | 0.293 (-0.143, 0.730); p = 0.188 |
|  |  | GSE186075 | 0.480 (-0.110, 1.071); p = 0.111 |
|  |  | GSE58934 | 0.189 (-0.273, 0.650); p = 0.423 |
|  |  | GSE59131 | 0.091 (-0.394, 0.576); p = 0.713 |

**S10. Sensitivity of Functional Enrichment Across Studies and Risk of Bias Assessment**

To assess potential small-study effects or asymmetry in functional enrichment across datasets, we applied Egger’s regression test to GSVA-derived pathway scores obtained through LOSO meta-analyses. A total of 105 pathway × contrast combinations met the inclusion criteria (≥5 iterations) for funnel plot analysis and Egger’s testing.

The majority of pathways (n = 87; 82.9%) showed no evidence of asymmetry (p > 0.05), suggesting overall robustness of the enrichment patterns. However, 14 combinations (13.3%) demonstrated possible asymmetry (p < 0.05), and 4 combinations (3.8%) were classified as likely biased (p < 0.01), indicating potential dataset-specific sensitivity.

Among the pathways with potential or likely bias were **Epidermal_Keratins**, **Sebocytes**, and **TGF-β/Fibrosis signaling**, particularly in the **CCCA vs Control** contrast. These findings may reflect either true biological heterogeneity in epithelial and fibrotic responses, or instability introduced by dataset-specific variation in gene coverage or annotation. No single dataset consistently accounted for asymmetry across multiple contrasts, although the influence of smaller or unpublished studies warrants cautious interpretation.

Funnel plots with 95% confidence boundaries and Egger’s regression lines were generated to visualize dispersion and bias (Supplementary Figure S6). A complete table summarizing the number of LOSO iterations, mean log₂ fold change, standard deviation, Egger’s p-value, and bias interpretation for each combination is provided in Supplementary Table S6.

These results indicate that while GSVA-based pathway analyses are generally stable across studies, a subset of pathways—particularly those related to epithelial and fibrotic remodeling—may be more sensitive to dataset composition, and should be interpreted in conjunction with sensitivity and meta-regression analyses.

**S11. Desviation from protocol**

This systematic review and meta-analysis was conducted according to the predefined methodology outlined in the PROSPERO registration (**CRD42024559969**), which was developed following PRISMA 2020, AMSTAR 2, and ROBIS guidelines.

No major deviations were made from the original protocol. All prespecified inclusion criteria, analytic strategies, and outcome definitions were followed. However, the following **minor modifications** were introduced during the conduct of the review to improve methodological rigor and data integration:

1. **Inclusion of RNA-seq datasets lacking normalized matrices**:
   For datasets such as **GSE186075** and **GSE125733**, which only provided FPKM or non-count-level data in GEO, we implemented a predefined secondary strategy included in the protocol: downloading raw SRA files, reprocessing FASTQ reads using **Salmon** in quasi-mapping mode, and summarizing transcript-level abundances to the gene level using **tximport**. This allowed uniform expression quantification and ensured inclusion without compromising comparability.
2. **Harmonization of control group structures**:
   As anticipated in the protocol, we included datasets using **pooled controls** (e.g., GSE58934, GSE59131) when no individual control samples were available, provided that batch correction and sensitivity analyses could be appropriately applied. This choice was retained to maximize inclusion and subtype representation, with caveats explicitly addressed in bias assessment and LOSO analyses.
3. **Adjustment of statistical thresholds**:
   The protocol prespecified a false discovery rate (FDR) threshold of **q < 0.1** for differential expression. However, during data integration, we adopted a **more conservative threshold of FDR < 0.05** and **|log₂FC| > 0.5** to enhance robustness and reduce false positives in the meta-analysis, especially given the high dimensionality of transcriptomic data.
4. **Refinement of GSVA gene sets**:
   While GSVA analysis was included in the original protocol, we extended the scope by incorporating **curated gene sets for sebaceous, epithelial, immune, and fibrotic signatures** derived from specialized databases and literature mining, to capture disease-relevant functional pathways beyond standard GO/Reactome categories.

All protocol changes were methodologically justified and documented prior to data analysis. The consistency between planned and executed methods supports the credibility, reproducibility, and transparency of our findings.

### ****SUPPLEMENTARY FIGURES****

### ****Fig. S1****

| 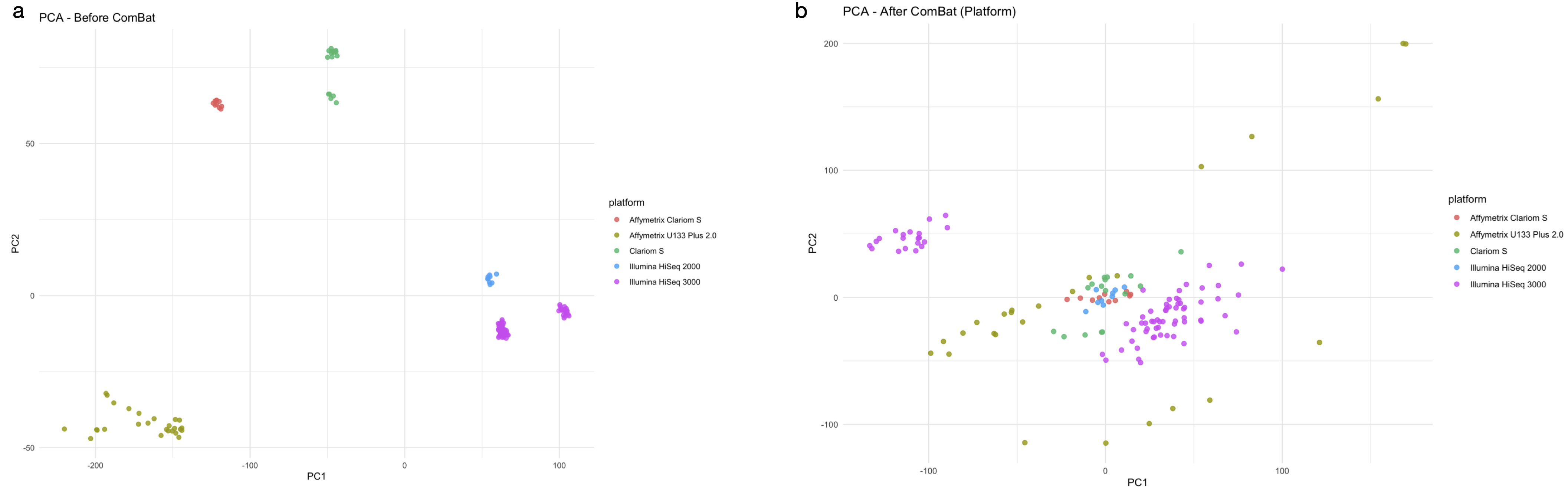 |
| --- |

### ****Fig. S1: Principal component analysis before and after batch correction****

Principal component analysis (PCA) plots of gene expression profiles for all samples included in the meta-analysis, coloured by platform and study.
a) PCA before batch correction shows strong segregation by platform and study, indicating technical confounding effects.
b) PCA after ComBat batch correction shows substantial reduction in batch effects, improving sample comparability across datasets.
This correction was essential to enable integrative analysis across RNA-seq and microarray datasets from different platforms and studies.

**Fig. S2**

|  |
| --- |

****Fig. S2: Hierarchical clustering of transcriptomes before batch correction.**** Heatmap showing hierarchical clustering of all samples based on transcriptome-wide expression prior to batch correction. Rows and columns represent samples, clustered by study and platform. The strong separation of clusters reflects technical variability between datasets. Annotations indicate group, disease severity, and platform. This figure illustrates the need for batch correction before integrative transcriptomic analysis.

**Fig. S3**

| 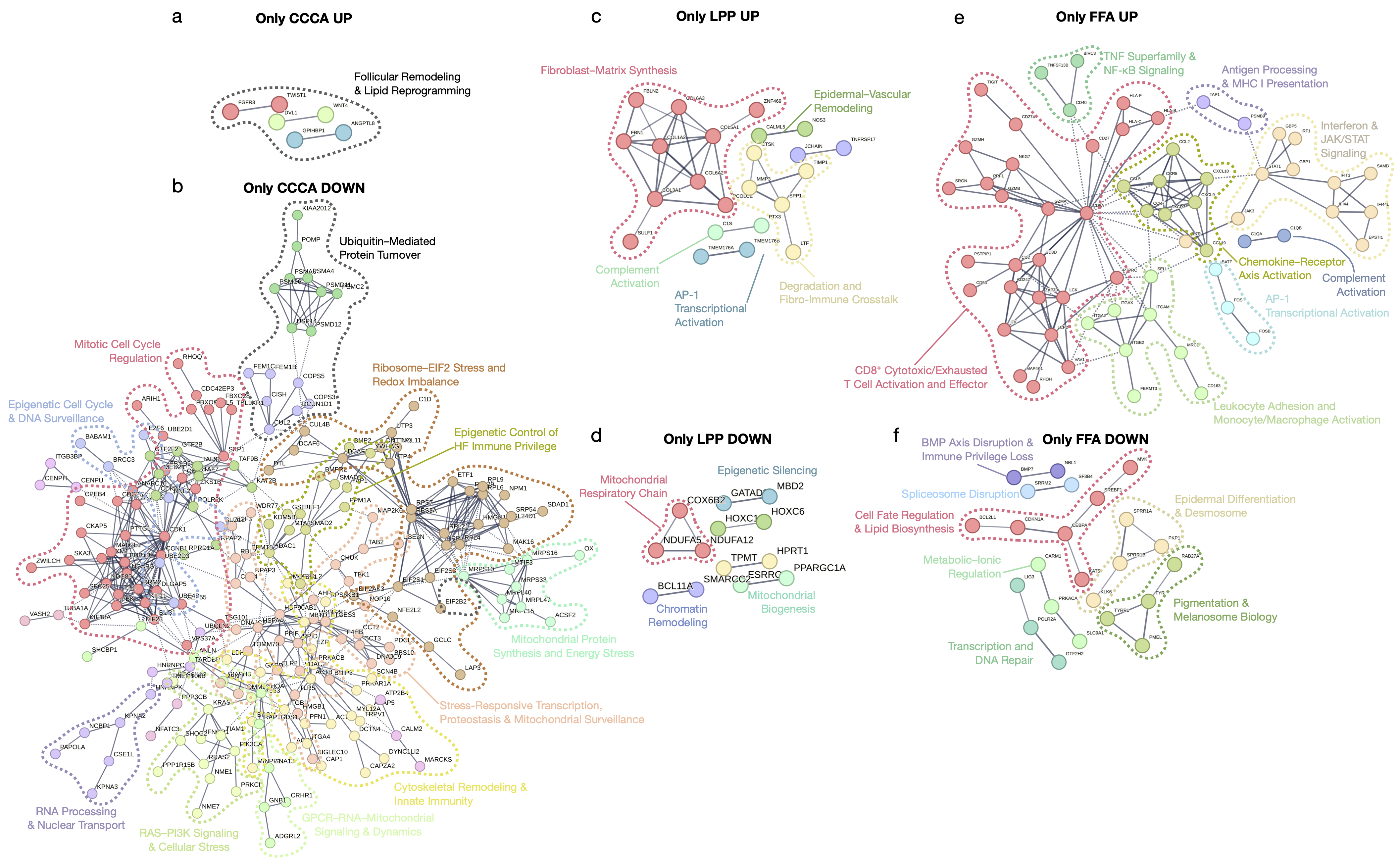 |
| --- |

**Fig. S3: Subtype-specific functional modules in primary lymphocytic scarring alopecias.
STRING-derived protein–protein interaction networks of differentially expressed genes exclusively altered in each subtype (FFA, LPP, CCCA). Modules are annotated by biological function and categorized into immune activation, epithelial structure, metabolism, epigenetics, and stromal remodeling.**

****Fig. S4****

| 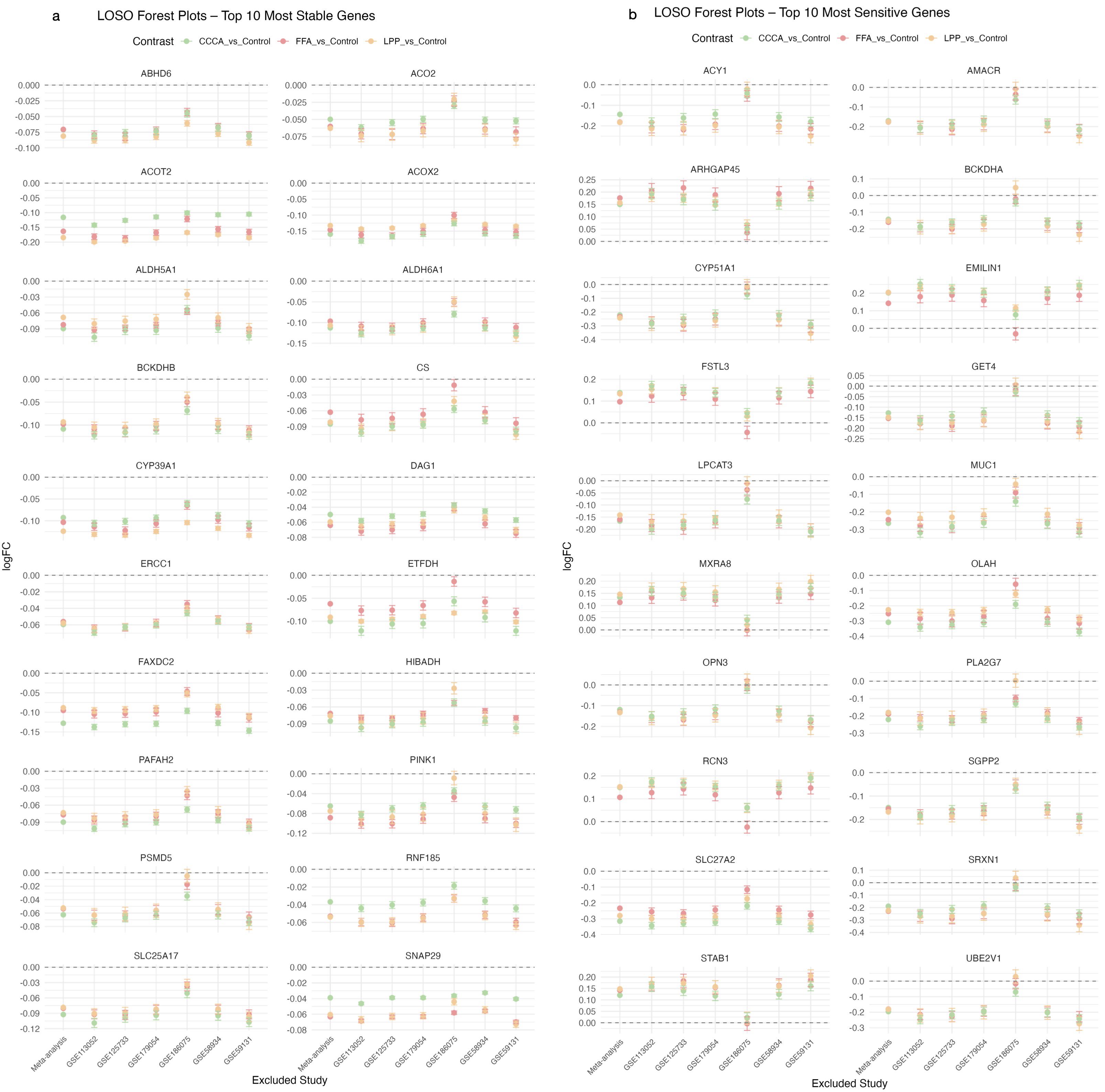 |
| --- |

**Fig. S4: Expression profiles of the most robust and most sensitive genes from LOSO analysis.** Stacked boxplots showing normalized expression levels for (a) the 10 most robust and (b) the 10 most sensitive genes identified by leave-one-study-out (LOSO) sensitivity analysis across scarring alopecia subtypes. Robustness and sensitivity were defined based on the stability of log₂ fold changes across LOSO iterations. Expression is shown for control, FFA, LPP, and CCCA samples after batch correction. Robust genes displayed consistent differential expression regardless of study exclusion, whereas sensitive genes exhibited substantial variability.

### ****Fig. S5.1****

|  |
| --- |

**Fig. S5.1. Leave-one-study-out sensitivity analysis of gene-level meta-analysis estimates (Panel 1). Forest plots illustrating odds ratios (ORs) and 95% confidence intervals (CIs) for selected differentially expressed genes across scarring alopecia subtypes, derived from leave-one-study-out (LOSO) meta-analyses. Each plot presents the pooled OR from the full meta-analysis (black diamond) alongside recalculated ORs after sequential exclusion of individual studies (squares with error bars). This panel displays the first subset of genes examined. Robust genes maintain stable ORs and statistical significance across iterations, whereas sensitive genes demonstrate notable shifts in effect size and significance depending on dataset inclusion.**

**Fig. S5.2**

|  |
| --- |

**Fig. S5.2. Leave-one-study-out sensitivity analysis of gene-level meta-analysis estimates (Panel 2). Second panel of the LOSO sensitivity analysis, extending the results shown in Supplementary Fig. S6.1. Forest plots display the pooled odds ratio (OR) from the complete meta-analysis (black diamond) alongside recalculated ORs after exclusion of individual studies (squares with 95% confidence intervals). This additional set of differentially expressed genes further illustrates the variability in effect estimates and highlights genes whose significance is disproportionately influenced by specific datasets.**
